# Effects of Digital Health Interventions in Chronic Musculoskeletal Pain: Systematic Review and Bayesian Network Meta-Analysis of Randomized Controlled Trials

**DOI:** 10.2196/95903

**Published:** 2026-10-07

**Authors:** Xuxin Wang, Wen Jing, Yunxia Li, Hui Li, Jing Li, Yuanyuan Liu, Su’e Yuan

**Affiliations:** 1 Teaching and Research Section of Clinical Nursing, Xiangya Hospital Central South University, Xiangya Hospital, Central South University, Changsha, Hunan, 410008, China, 86 13873158943, 86 13873158943; 2 School of Nursing, Lanzhou University, Lanzhou, Gansu, China

**Keywords:** chronic musculoskeletal pain, digital health interventions, Bayesian network meta-analysis, randomized controlled trials, virtual reality, telerehabilitation, mobile apps

## Abstract

**Background:**

Chronic musculoskeletal pain is a major public health problem, and access to continuous, long-term care remains challenging. Digital health interventions may extend care beyond conventional settings; however, their comparative effects across delivery modalities and core therapeutic components remain uncertain.

**Objective:**

This study aimed to synthesize evidence and compare the effects of different digital health intervention modalities on pain, functional disability, and health-related quality of life in adults with chronic musculoskeletal pain.

**Methods:**

This systematic review and Bayesian network meta-analysis followed the PRISMA (Preferred Reporting Items for Systematic Reviews and Meta-Analyses) 2020 guidelines. PubMed, the Cochrane Central Register of Controlled Trials, Embase, Web of Science, CINAHL, MEDLINE, and Scopus were systematically searched from database inception to January 29, 2026. Randomized controlled trials comparing digital health interventions with control conditions in adults with chronic musculoskeletal pain were included. Interventions were classified according to digital delivery format and core therapeutic components. Standardized effect sizes (Hedges *g*) were used, and pairwise meta-analyses and Bayesian random-effects network meta-analyses were conducted. Subgroup analyses explored effect modification by core components. Risk of bias was assessed using the Cochrane Risk of Bias 2 tool, and certainty of evidence was evaluated using the Grading of Recommendations Assessment, Development and Evaluation (GRADE) framework adapted for network meta-analysis.

**Results:**

Ninety-two randomized controlled trials involving 12,595 participants were included. Pairwise meta-analyses suggested small to moderate favorable average effects of app-based, virtual reality–based, and telerehabilitation interventions on pain and functional disability, whereas estimates for wearable device and multimodal interventions were imprecise. However, the prediction intervals crossed the null, suggesting that benefits may not be consistent across settings. Component-based analyses suggested larger effects for exercise or motor function training than for interventions centered primarily on education or cognitive behavioral therapy; however, several subgroups included few studies. In the Bayesian network meta-analyses, motor function–oriented virtual reality and exercise-based telerehabilitation showed potentially favorable effects across pain and disability outcomes, while some virtual reality interventions ranked relatively highly for pain. However, these rankings remained uncertain because of heterogeneity, imprecision, and limited direct evidence for some comparisons. Network meta-regression suggested that intervention duration may be associated with functional disability outcomes, whereas the corresponding association for pain was less certain. The certainty of evidence was predominantly low or very low.

**Conclusions:**

Digital health interventions may provide small to moderate average improvements in pain and disability, but their clinical importance and comparative effects remain uncertain. Unlike previous reviews focused on individual technologies or broad delivery categories, this systematic review integrated delivery modality with core therapeutic content. This dual-dimensional framework provides a more clinically interpretable basis for comparing interventions and prioritizing future head-to-head trials. Exercise-based virtual reality and telerehabilitation appear promising but cannot be considered superior. Future research should standardize intervention content and conduct high-quality head-to-head trials to confirm long-term effects.

## Introduction

Chronic musculoskeletal pain (CMP) is defined as noncancer pain originating from muscles, bones, joints, tendons, or related soft tissues that persists or recurs for a period exceeding 3 months [1]. A systematic review and meta-analysis covering 28 countries found that CMP affected approximately 26% of the adult population [2]. CMP is frequently accompanied by varying degrees of functional limitation and reduced health-related quality of life [3]. Low back pain, neck pain, and osteoarthritis-related pain consistently rank among the leading causes of years lived with disability worldwide [4,5]. Given its high prevalence, chronic course, and substantial disability burden, CMP has become a major public health and clinical management challenge [6].

CMP is commonly managed using multimodal strategies, including health education, exercise therapy, behavioral support, and cognitive behavioral therapy [7]. However, conventional interventions are largely dependent on face-to-face delivery and continuous involvement of health care professionals [8]. Their implementation frequency, accessibility, and long-term follow-up capacity are often constrained by health care resource allocation, geographic barriers, and implementation costs [9]. These limitations can impede sustained patient engagement and limit the scalability of standardized interventions in underserved regions or among individuals with limited mobility [10]. Consequently, there is an imperative to investigate intervention strategies that can facilitate long-term management while concurrently ensuring clinical effectiveness, accessibility, and scalability.

Digital health interventions (DHIs) have been increasingly applied in the management of CMP [11], including mobile app–based self-management programs, therapist-led telerehabilitation, wearable-supported monitoring and feedback systems, virtual reality–based interventions, and multicomponent interventions integrating multiple digital technologies and therapeutic elements [12]. The use of DHIs has been demonstrated to facilitate the mitigation of temporal and geographical limitations, thereby fostering long-term self-management capabilities through remote delivery, personalized feedback, behavioral support, and sustained interaction between patients and health care professionals [13]. However, it is important to note that digital technologies primarily function as delivery and interaction platforms and are not equivalent to specific therapeutic mechanisms. For example, mobile apps, telerehabilitation, and virtual reality may each be used to deliver different therapeutic components, including exercise, education, behavioral or psychological support, and perceptual or attentional modulation [14-16]. The effectiveness of DHIs may therefore be jointly influenced by both the digital delivery format and the core therapeutic components. Consequently, classifying DHIs solely according to technological platform may group interventions with different therapeutic purposes and mechanisms within the same node. This may obscure genuine within-node effect heterogeneity, increase clinical heterogeneity, and weaken the transitivity and interpretability of network comparisons.

Previous systematic reviews and traditional pairwise meta-analyses have evaluated the effects of DHIs on CMP [12,17,18]. However, the majority of studies have focused on single technological platforms and have primarily compared DHIs with conventional care, thereby addressing whether a given digital intervention is effective but not allowing simultaneous comparisons of the relative effectiveness of different DHIs. In contrast, extant network meta-analyses facilitate comparisons across multiple DHIs within a unified framework [19,20], but their classifications have generally not adequately accounted for differences in core therapeutic components within the same technological platform. Consequently, the comparative effectiveness of intervention nodes jointly defined by technological platforms and core therapeutic components remains unclear. A systematic comparison of DHIs using a classification framework that considers both dimensions is therefore warranted. Such a bidimensional classification framework may generate more clinically meaningful intervention nodes by distinguishing among different combinations of technological platforms and core therapeutic components. This distinction may help clinicians identify more appropriate and effective combinations and assist policymakers in identifying scalable implementation strategies while considering the clinical resources required to deliver different therapeutic components. Furthermore, functional disability, health-related quality of life, and the certainty of evidence derived from network comparisons have not yet been comprehensively synthesized.

In summary, this systematic review and Bayesian network meta-analysis classified randomized controlled trials using a bidimensional framework integrating digital delivery formats and core therapeutic components. This framework was intended to improve the clinical homogeneity of intervention nodes and the interpretability of network comparisons. This systematic review aimed to compare the relative effects of different combinations of digital delivery formats and core therapeutic components on pain intensity and functional disability in adults with CMP. Direct and indirect evidence was integrated to estimate the relative effects and rank the interventions. Health-related quality of life was descriptively synthesized, and the certainty of evidence from network comparisons was assessed. Together, these analyses were designed to provide more targeted evidence for clinical decision-making and future research on DHIs for CMP.

## Methods

### Study Design and Reporting Standards

This systematic review and network meta-analysis were registered in the International Prospective Register of Systematic Reviews (PROSPERO; CRD420251169866). The design, conduct, and reporting of this systematic review followed the PRISMA (Preferred Reporting Items for Systematic Reviews and Meta-Analyses) 2020 statement, the PRISMA extension for network meta-analyses (PRISMA-NMA), and the PRISMA literature search extension (PRISMA-S) for reporting literature searches in systematic reviews [21,22]. The complete PRISMA 2020, PRISMA-NMA, and PRISMA-S checklists are provided in Checklist 1.

### Information Sources and Search Strategy

A systematic search was conducted across 7 electronic databases: PubMed, Embase, Web of Science Core Collection, the Cochrane Central Register of Controlled Trials, CINAHL, MEDLINE, and Scopus. The search covered each database from inception to July 10, 2025. The search strategy combined controlled vocabulary terms and free-text keywords covering CMP, DHIs, mobile apps, virtual reality, wearable devices, telerehabilitation, and randomized controlled trials. Search strategies were adapted to each database’s indexing terms and syntax. The strategy was developed by the research team and validated against preidentified relevant studies to ensure sensitivity. An updated search was conducted on January 29, 2026, using identical databases, strategy, and eligibility criteria to identify newly published records. Full search strategies and results are provided in Multimedia Appendix 1. Reference lists of included studies and relevant systematic reviews were manually screened, and forward citation tracking was performed where appropriate. Gray literature databases and clinical trial registries were not searched separately.

All records were imported into EndNote 21 (Clarivate) for deduplication. Two reviewers (XW and WJ) independently screened titles, abstracts, and full texts. Disagreements were resolved through discussion or adjudication by a third reviewer (Y Liu). Multiple reports of the same trial were merged using the registration number, study center, recruitment period, sample characteristics, intervention details, and baseline data. The most complete report was used as the primary source.

### Eligibility Criteria

Eligibility criteria were defined according to the PICOS (population, intervention, comparison, outcome, and study design) framework [23].

#### Participants

Adults aged ≥18 years with clinically diagnosed CMP were included. CMP was defined as pain originating from musculoskeletal structures that persisted or recurred for at least 3 months. Conditions included chronic low back pain, neck pain, osteoarthritis-related pain, shoulder pain, fibromyalgia, and related disorders. Studies were excluded if they involved cancer-related pain, primary neuropathic pain, acute injuries, or pain caused by systemic inflammatory or structural disorders. Mixed-population studies were included only when data for eligible participants could be separately extracted.

#### Interventions

For this systematic review, interventions were classified as DHIs if digital technology served as the primary means of delivering therapeutic content. This classification was informed by the World Health Organization classification framework for DHIs. After the eligible interventions were characterized, each study arm was classified using a 2-level hierarchical coding scheme. At the first level, interventions were classified by primary digital delivery modality into 5 categories: mobile apps, virtual reality, wearable devices, telerehabilitation, and multimodal digital interventions. Interventions that integrated 2 or more digital delivery modalities as substantive treatment components were classified as multimodal digital interventions. At the second level, interventions were subclassified by their core therapeutic components or primary therapeutic functions. Each study arm was assigned to a single, mutually exclusive node. Ancillary features, such as reminders, technical assistance, or brief supplementary contact, did not influence node assignment unless they represented substantive therapeutic components. Only active intervention categories represented in at least 3 eligible studies were included as separate nodes in the network meta-analysis, yielding 12 active intervention nodes. Control conditions were coded as active or nonactive for descriptive analyses and network meta-regression but were combined into a single control node for the primary network meta-analysis. Thus, the final network comprised 13 nodes. Detailed operational definitions, classification boundaries, and study arm–to–node assignments are provided in Multimedia Appendix 2.

#### Comparators

Comparators included usual care, waiting list, no treatment, educational materials, placebo-like interventions, and face-to-face care. Controls were categorized as inactive or active. Inactive controls included a waiting list, no treatment, or minimal support. Active controls included structured rehabilitation, exercise, education, or other nondigital interventions [24]. In the primary analysis, all controls were combined into a single node. Control type was further explored as an effect modifier in network meta-regression.

#### Outcomes

The primary outcome was pain intensity. Secondary outcomes included functional disability and health-related quality of life. Pain intensity was measured using visual analogue scale (VAS), numeric rating scale (NRS), or validated instruments. Functional disability was assessed using disease-specific or generic functional scales. Health-related quality of life was measured using validated instruments (Multimedia Appendix 3).

#### Study Design

Only randomized controlled trials (parallel or multiarm) were included.

Observational studies, nonrandomized studies, qualitative studies, reviews, protocols, and conference abstracts were excluded.

### Data Extraction

A standardized extraction form was developed and piloted. Two reviewers independently extracted data. Disagreements were resolved by discussion or third-party adjudication. Extracted data included study characteristics, participant characteristics, intervention and comparator details, outcome measures, and quantitative outcome data. For continuous outcomes, means, SDs, and sample sizes were extracted. Change scores were preferentially used; end point values were used when change scores were unavailable. Missing SDs were calculated from available statistics or imputed using recommended methods [25,26]. If key data could not be obtained from the original articles or supplementary materials or by contacting the authors and could not be reliably converted, the study was excluded from the quantitative synthesis for the corresponding outcome. For studies reporting multiple follow-up time points, data from the final assessment time point were extracted for analysis, and the timing of assessment was considered in assessing transitivity and in network meta-regression.

### Outcome Measures

Outcomes were synthesized using standardized mean differences (SMDs) with Hedges *g* correction. All outcomes were aligned so that negative values indicated improvement in favor of DHIs. Scales with opposite directionality were reversed before analysis. Functional disability was defined solely by patient-reported functional outcomes. Objective performance measures and psychological constructs were not pooled with disability outcomes. Health-related quality of life was not quantitatively pooled due to heterogeneity and was synthesized narratively.

### Risk of Bias Assessment

Risk of bias was assessed using the Cochrane Risk of Bias 2 (RoB 2) tool for randomized trials [27]. Two reviewers independently evaluated each outcome. Domains included randomization, deviations from intended interventions, missing data, outcome measurement, and selective reporting. Each domain was rated as low risk, some concerns, or high risk. Assessments were performed separately by outcome and time point. Disagreements were resolved by consensus or adjudication.

### Statistical Analysis

#### Pairwise Meta-Analysis

Pairwise meta-analyses were conducted using R software (version 4.4.2; R Foundation for Statistical Computing) with the *meta* package. Interventions were grouped by technological platform (mobile apps, virtual reality, wearable devices, telerehabilitation, and multimodal interventions). Subgroup analyses explored core therapeutic components. Random-effects models were applied. Between-study variance (*τ*²) was estimated using restricted maximum likelihood, and the Hartung-Knapp adjustment was used to calculate 95% CIs. *I*² was interpreted as a relative measure of inconsistency rather than as an absolute measure of heterogeneity, and Cochran Q test was used to evaluate evidence against homogeneity. For each modality-specific meta-analysis, 95% prediction intervals were calculated to describe the range of true effects expected in a future comparable study or clinical setting [28,29]. Sensitivity analyses used leave-one-out methods. Small-study effects were assessed using funnel plots and Egger test when ≥10 studies were available.

#### Bayesian Network Meta-Analysis

Network meta-analysis was conducted using the *gemtc* package in R (version 4.4.2). The network included 13 nodes (12 intervention nodes and 1 control node) [30]. Quantitative synthesis was performed only when sufficient clinical comparability and network connectivity were present [31]. Otherwise, results were summarized narratively. Arm-level data were used, and correlations in multiarm trials were modeled appropriately [32]. Node size reflected sample size, and edge thickness reflected the number of direct comparisons. Four Markov chains were run (20,000 burn-in + 50,000 iterations). Convergence was assessed using the Brooks-Gelman-Rubin diagnostic, trace plots, and density plots. All effects were expressed as SMDs with 95% credible intervals (CrIs). Transitivity was assessed based on potential effect modifiers including age, disease type, baseline severity, intervention duration, follow-up time, control type, professional support, and RoB 2. Local inconsistency was assessed using node splitting. Between-study variability was quantified using the posterior distribution of the between-study SD. Treatment ranking was based on rank probabilities, mean ranks, and surface under the cumulative ranking curve (SUCRA). The highest-ranked treatment was defined as rank 1 after harmonization of effect direction.

#### Network Meta-Regression

Univariable random-effects network meta-regression models were used to assess associations between study-level covariates and treatment effects. Covariates included RoB 2, mean age, intervention duration, follow-up time, and control type. Continuous variables were modeled linearly; categorical variables were dummy coded. Models were estimated using MCMC. Model fit was evaluated using deviance information criterion (DIC), residual deviance, heterogeneity, and convergence diagnostics. A DIC reduction >10 suggested improved fit.

### Certainty of Evidence

The certainty of evidence was assessed using the Grading of Recommendations Assessment, Development and Evaluation (GRADE) approach adapted for network meta-analysis [33-35]. For each outcome-specific intervention-vs-control network estimate, the standard GRADE domains of risk of bias, inconsistency, indirectness, imprecision, and publication bias were considered. Evidence from randomized controlled trials was initially rated as high certainty and was rated down by 1 or 2 levels for serious or very serious concerns, respectively. Final certainty was categorized as high, moderate, low, or very low. Two reviewers independently performed the assessments, and disagreements were resolved through consensus or consultation with a third reviewer.

## Results

### Study Selection

The study selection process for this systematic review is presented in the PRISMA flow diagram (Figure 1). A total of 9235 records were identified through systematic database searching, including records retrieved from the initial search and the presubmission update. Of these, 1649 records were retrieved from PubMed, 1390 from Embase, 1415 from Web of Science, 726 from the Cochrane Library, 1852 from CINAHL, 982 from MEDLINE, and 1221 from Scopus. After duplicates were removed in EndNote, 3976 records were excluded, leaving 5259 for title and abstract screening. In the initial screening phase, 4628 records were excluded, including 2594 that did not meet the eligibility criteria for study design and 2034 that did not meet the inclusion criteria for the research topic.

Subsequently, 631 full-text articles were assessed for eligibility. Of these, 49 were excluded because the full text could not be retrieved. Other reasons for exclusion included ineligible population (n=174), ineligible intervention (n=131), irrelevant outcomes (n=83), inappropriate study design (n=51), and review articles (n=51). Ultimately, 92 articles were included in the systematic review.

**Figure 1.**
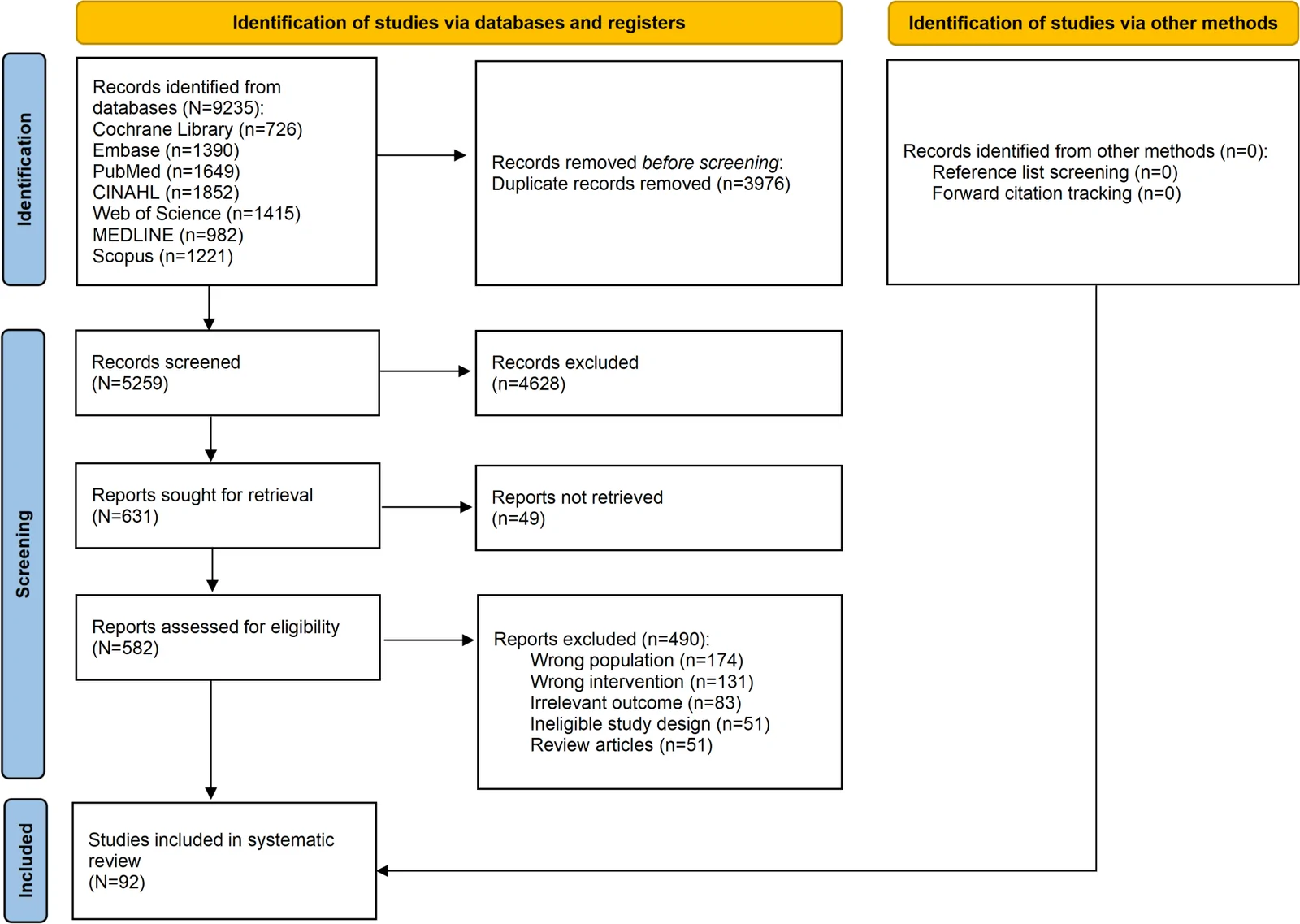
PRISMA (Preferred Reporting Items for Systematic Reviews and Meta-Analyses) flow diagram of study selection.

### Characteristics of Included Studies

A total of 92 randomized controlled trials were included in this systematic review, involving 12,595 participants [36-127]. The overall mean age of participants across studies was 51.33 years. The mean intervention duration was 9.15 (SD 6.10) weeks, ranging from 1 to 36 weeks. The included studies were conducted across 28 countries. Turkey [37,42,43,59,85,92,93,95-97,107,119] and the United States [46,48,49,56,63,69,80,81,101,103,118,125] contributed the highest number of studies (n=12 each), followed by Germany and Spain (n=9 each) [50,51,58,64,74,78,98,102,106,110,114-116,120-124]; Australia (n=8) [39,41,65,66,82,100,108,109]; South Korea (n=5) [71,72,75,76,99]; China and Iran (n=4 each) [36,47,60,67,79,111,113,127]; and the United Kingdom [52,55,112], Thailand [49,92,117], Japan [61,62,104], and Sweden [70,73,94] (n=3 each). In addition, 2 multicountry trials were included, one conducted in Egypt and Saudi Arabia [87] and another in Norway and Denmark [105]. Detailed study characteristics are presented in Multimedia Appendix 4.

All 92 trials reported pain outcomes, and 77 reported functional disability outcomes [36-51,53-56,58-69,71,72,74-77,80-86,88-90,92-103,105-110,112-114,116,117,119,120,122,123,125-127], which were included in the corresponding analyses according to available data. Among the included trials, 51 reported quality of life outcomes [36,38,40-43,48,50,51,53,57-59,62-66,68,70,71,74,76-79,82-86,91,92,94-96,99,104-106,108,110,113,114,117,119-121,123,126,127], of which 46 (90.2%) indicated improvements in quality of life [36,38,40,42,43,48,50,51,53,58,62-65,68,70,71,74,76-79,82-86,91,92,95,96,99,104-106,108,110,113,114,117,119-121,123,126,127], while 5 (9.8%) showed no clear advantage [41,57,59,66,94]. Detailed quality of life results are presented in Multimedia Appendix 5.

### Risk of Bias

Risk of bias was assessed at the outcome level using the RoB 2 tool. Overall, of the 92 studies, 21 (22.8%) were judged to have a low risk of bias, 67 (72.8%) to have some concerns, and 4 (4.3%) to have a high risk of bias. A high risk of bias was primarily attributed to missing outcome data without appropriate analytical handling, deviations from intended interventions, and bias in outcome measurement. In some studies, multiple domains were rated as high risk. An overall summary is presented in Figure 2, and detailed RoB 2 judgments and supporting information are provided in Multimedia Appendix 6.

**Figure 2.**
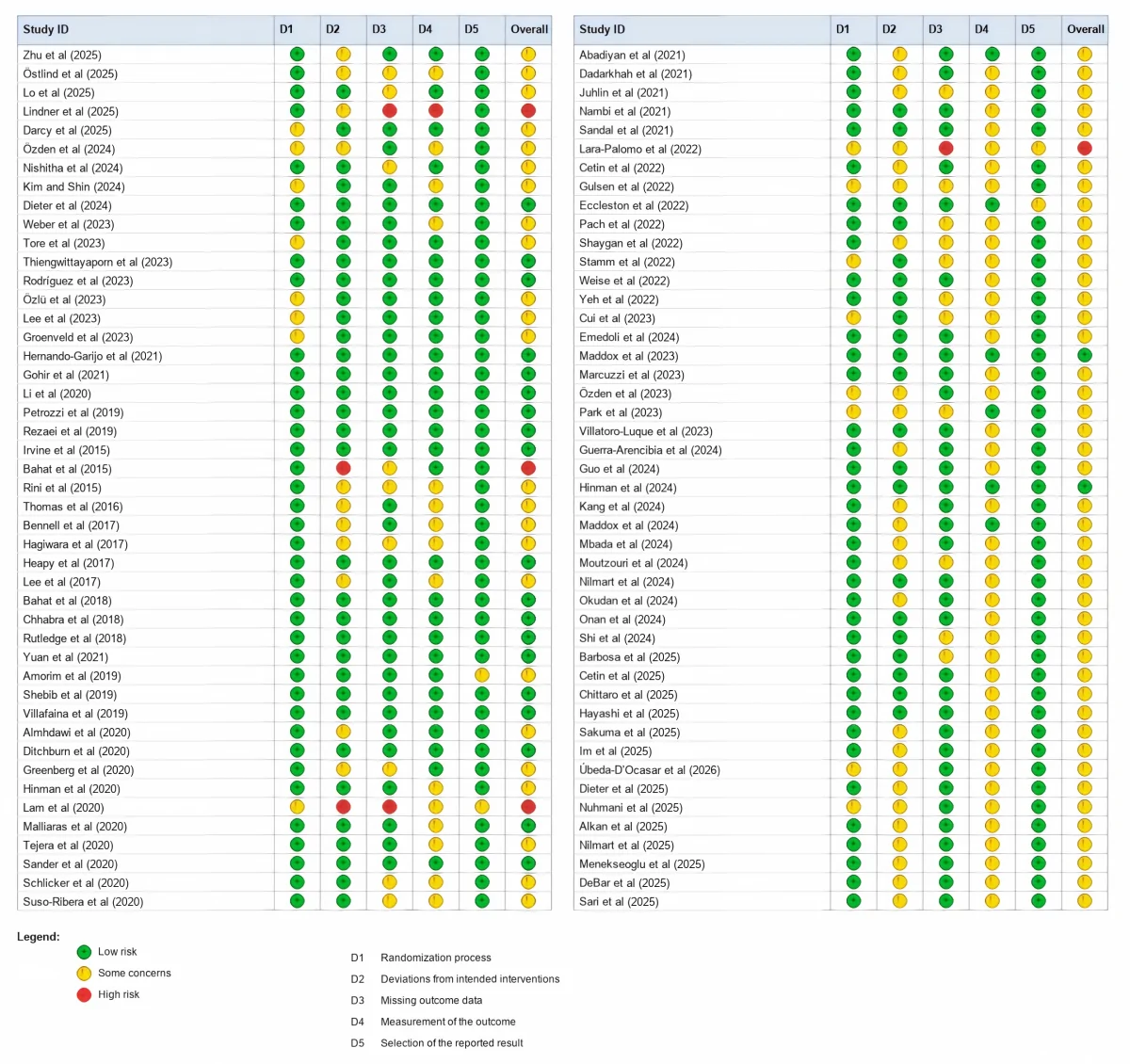
Study-level risk-of-bias judgments using the Cochrane Risk of Bias 2 (RoB 2) tool. The traffic light plot presents domain-specific and overall judgments for each included randomized controlled trial. D1, bias arising from the randomization process; D2, bias due to deviations from intended interventions; D3, bias due to missing outcome data; D4, bias in outcome measurement; and D5, bias in selection of the reported result. Green circles with a plus sign indicate a low risk of bias, yellow circles with an exclamation mark indicate some concerns, and red circles with a minus sign indicate a high risk of bias [36-127].

### Certainty of Evidence

The certainty of evidence for the network estimates of pain intensity and functional disability was assessed using the GRADE approach adapted for network meta-analysis. Overall, the certainty of evidence was low or very low, with no comparisons rated as moderate or high certainty. Rating down was primarily attributable to risk of bias and imprecision, with heterogeneity and potential publication bias contributing to some assessments. Indirectness was not considered serious, and no serious incoherence between direct and indirect evidence was identified, although the ability to detect incoherence was limited for sparsely informed comparisons. Network estimates, judgments across the 5 standard GRADE domains, reasons for rating down, and final certainty ratings are presented in Table 1.

**Table 1.** GRADE^a^ evidence profile for network estimates of digital health interventions vs control for pain intensity and functional disability.

Intervention vs control (node)	Direct evidence, number of studies (number of participants)	Network estimate SMD^b^ (95% CrI)^c^	GRADE domain					Certainty
			Risk of bias	Inconsistency	Indirectness	Imprecision	Publication bias	
Pain intensity								
APP-EX^d^ (A)	13 (941)	−0.23 (−0.42 to −0.04)	Serious	Not serious	Not serious	Serious	Not serious	⨁⨁◯◯ Low
APP-CBT^e^ (B)	5 (2513)	−0.04 (−0.26 to 0.19)	Serious	Serious	Not serious	Serious	Not serious	⨁◯◯◯ Very low
APP-EX+EDU^f^ (C)	11(1355)	−0.28 (−0.46 to −0.1)	Serious	Serious	Not serious	Serious	Not serious	⨁◯◯◯ Very low
APP-EX +CBT+EDU^g^ (D)	3 (458)	−0.01 (−0.38 to 0.36)	Serious	Not serious	Not serious	Very serious	Not serious	⨁◯◯◯ Very low
MF-VR^h^ (E)	14 (641)	−0.4 (−0.6 to −0.2)	Serious	Not serious	Not serious	Not serious	Serious	⨁⨁◯◯ Low
CP-VR^i^ (F)	8 (2467)	−0.24 (−0.46 to −0.03)	Serious	Not serious	Not serious	Serious	Serious	⨁◯◯◯ Very low
DA-VR^j^ (G)	3 (80)	−0.55 (−1.1 to −0.02)	Serious	Serious	Not serious	Serious	Serious	⨁◯◯◯ Very low
WD^k^ (H)	4 (328)	−0.06 (−0.37 to 0.25)	Serious	Not serious	Not serious	Very serious	Not serious	⨁◯◯◯ Very low
TR-EX^l^ (I)	13 (670)	−0.31 (−0.52 to −0.11)	Serious	Serious	Not serious	Serious	Not serious	⨁◯◯◯ Very low
TR-EX+EDU^m^ (J)	8 (830)	−0.17 (−0.4 to 0.05)	Serious	Not serious	Not serious	Serious	Not serious	⨁⨁◯◯ Low
TR-CBT^n^ (K)	3 (1447)	−0.14 (−0.42 to 0.16)	Serious	Serious	Not serious	Serious	Not serious	⨁◯◯◯ Very low
MDI^o^ (L)	4 (416)	−0.08 (−0.39 to 0.22)	Serious	Not serious	Not serious	Very serious	Not serious	⨁◯◯◯ Very low
Functional disability								
APP-EX (A)	10 (588)	−0.33 (−0.59 to −0.08)	Serious	Not serious	Not serious	Serious	Not serious	⨁⨁◯◯ Low
APP-CBT (B)	4 (2293)	−0.1 (−0.42 to 0.23)	Serious	Not serious	Not serious	Very serious	Not serious	⨁◯◯◯ Very low
APP-EX+EDU (C)	11 (1355)	−0.14 (−0.36 to 0.08)	Serious	Not serious	Not serious	Serious	Not serious	⨁⨁◯◯ Low
APP-EX+CBT+EDU (D)	2 (438)	−0.05 (−0.54 to 0.47)	Serious	Serious	Not serious	Very serious	Not serious	⨁◯◯◯ Very low
MF-VR (E)	10 (412)	−0.45 (−0.73 to −0.17)	Serious	Not serious	Not serious	Serious	Serious	⨁◯◯◯ Very low
CP-VR (F)	5 (2309)	−0.28 (−0.61 to 0.03)	Serious	Serious	Not serious	Serious	Serious	⨁◯◯◯ Very low
DA-VR (G)	3 (80)	−0.55 (−1.17 to 0.04)	Serious	Serious	Not serious	Serious	Serious	⨁◯◯◯ Very low
WD (H)	4 (348)	−0.15 (−0.52 to 0.22)	Serious	Not serious	Not serious	Very serious	Not serious	⨁◯◯◯ Very low
TR-EX (I)	12 (638)	−0.38 (−0.64 to −0.14)	Serious	Serious	Not serious	Serious	Not serious	⨁◯◯◯ Very low
TR-EX+EDU (J)	8 (830)	−0.2 (−0.5 to 0.07)	Serious	Serious	Not serious	Serious	Not serious	⨁◯◯◯ Very low
TR-CBT (K)	3 (1447)	−0.03 (−0.4 to 0.35)	Serious	Serious	Not serious	Very serious	Not serious	⨁◯◯◯ Very low
MDI (L)	4 (416)	−0.05 (−0.41 to 0.33)	Serious	Not serious	Not serious	Very serious	Not serious	⨁◯◯◯ Very low

a GRADE: Grading of Recommendations Assessment, Development and Evaluation.

b SMD: standardized mean difference.

c CrI: credible interval.

d APP-EX: app-based exercise.

e APP-CBT: app-based cognitive behavioral therapy.

f APP-EX+EDU: app-based exercise plus education.

g APP-EX+CBT+EDU: app-based intervention combining exercise, cognitive behavioral therapy, and education.

h MF-VR: motor function virtual reality.

i CP-VR: cognitive/perceptual virtual reality.

j DA-VR: distraction virtual reality.

k WD: wearable devices.

l TR-EX: telerehabilitation exercise.

m TR-EX+EDU: telerehabilitation exercise plus education.

n TR-CBT: telerehabilitation cognitive behavioral therapy.

o MDI: multimodal digital intervention.

### Pairwise Meta-Analyses

#### Pain Intensity

The pairwise meta-analysis of pain intensity included 92 studies across 5 categories of digital health intervention delivery formats: app-based interventions, virtual reality, wearable devices, telehealth-based interventions, and multimodal digital interventions [36-127] (Figure 3). App-based, virtual reality–based, and telehealth-based interventions were associated with greater reductions in pain intensity than controls, with pooled Hedges *g* estimates ranging from –0.3 to –0.2 and 95% CIs excluding zero. By contrast, the estimates for wearable device and multimodal digital interventions were imprecise, with 95% CIs that included zero. Nevertheless, the 95% prediction intervals for app-based, virtual reality–based, and telerehabilitation interventions crossed the null, suggesting that the magnitude and direction of their effects may vary across future similar settings. The *I*^2^ and *τ*^2^ estimates, *P* values from Cochran Q tests, and 95% prediction intervals are presented in Figure 3. Subgroup analyses by therapeutic component suggested larger effects for exercise- and motor function–oriented interventions than for education-, CBT-, and distraction-oriented interventions; however, several estimates remained imprecise because they were based on few studies (Multimedia Appendix 7).

Among intervention categories with a sufficient number of studies, Egger regression test indicated potential small-study effects only for virtual reality interventions. Trim-and-fill analyses suggested the presence of potentially missing studies but did not alter the direction of the pooled estimate (Multimedia Appendix 8). Leave-one-out analyses did not materially change the pooled estimates or their interpretation (Multimedia Appendix 9).

**Figure 3.**
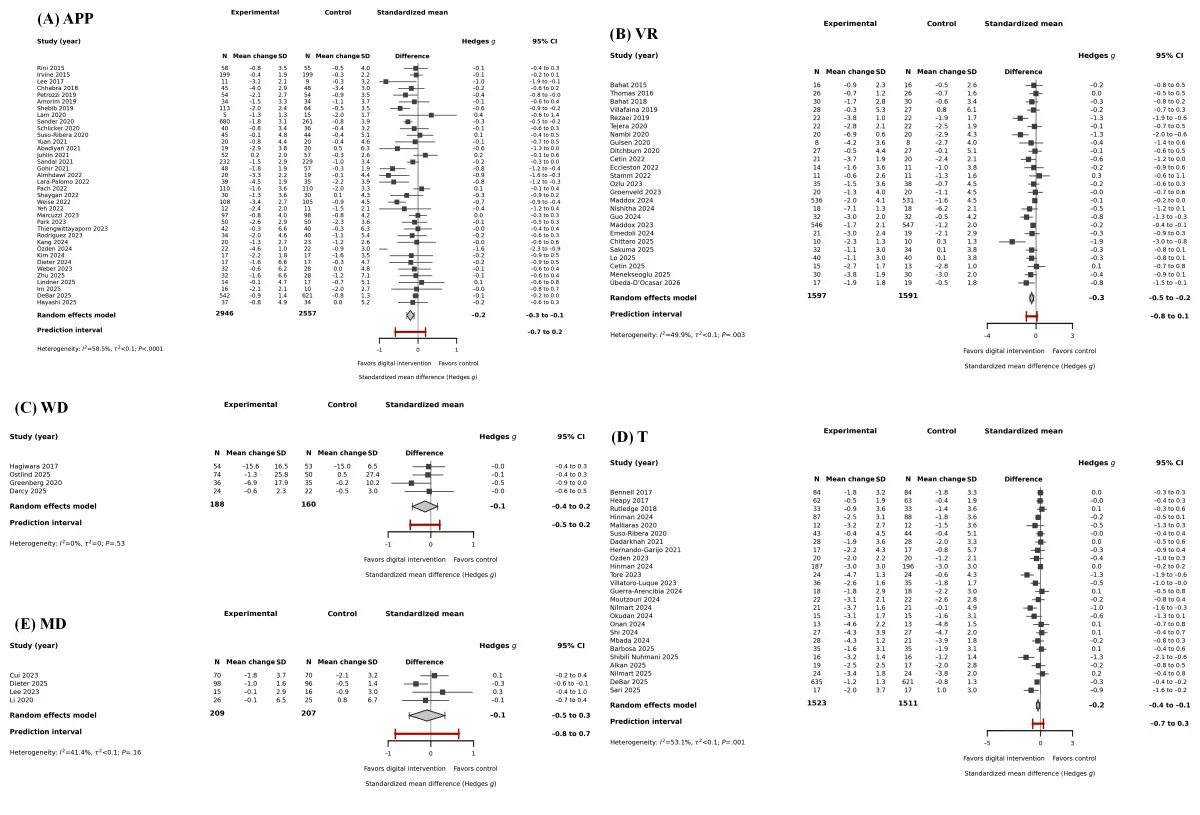
Forest plots of pairwise meta-analyses for pain outcomes across digital health intervention modalities. The plots present pooled effects of 5 categories of digital health interventions on pain outcomes in patients with chronic musculoskeletal pain. Panels A-E correspond to app-based interventions (APP), virtual reality interventions (VR), wearable devices (WD), telehealth-based interventions (T), and multimodal digital interventions (MD). Random-effects models were used, and results are reported as Hedges *g* with 95% CIs. Squares represent individual study estimates (size proportional to weight), horizontal lines represent 95% CIs, diamonds represent pooled estimates, and red lines indicate prediction intervals. Negative values indicate greater pain reduction, favoring digital interventions over the control condition [36-127].

#### Functional Disability

The pairwise meta-analysis of functional disability included 77 studies covering the same 5 categories of DHIs (Figure 4). App-based, virtual reality, and telehealth-based interventions favored the intervention groups, with pooled Hedges *g* estimates ranging from –0.4 to –0.2 and 95% CIs excluding zero. By contrast, the estimates for wearable device and multimodal digital interventions were imprecise, with 95% CIs that included zero. However, the corresponding 95% prediction intervals for app-based, virtual reality, and telerehabilitation interventions crossed zero, suggesting that benefits may not be consistent across future comparable settings. The *I*^2^ and *τ*^2^ estimates, *P* values from Cochran Q tests, and 95% prediction intervals are presented in Figure 4. Subgroup analyses by therapeutic component suggested larger effects for exercise-oriented interventions than for CBT-related and some multimodal interventions; however, estimates for several subgroups remained imprecise and should be considered exploratory (Multimedia Appendix 7).

Egger test indicated potential small-study effects only for virtual reality interventions. Full funnel plots, Egger test results, and trim-and-fill analyses are provided in Multimedia Appendix 8. Leave-one-out analyses did not materially change the pooled estimates or their interpretation (Multimedia Appendix 9).

**Figure 4.**
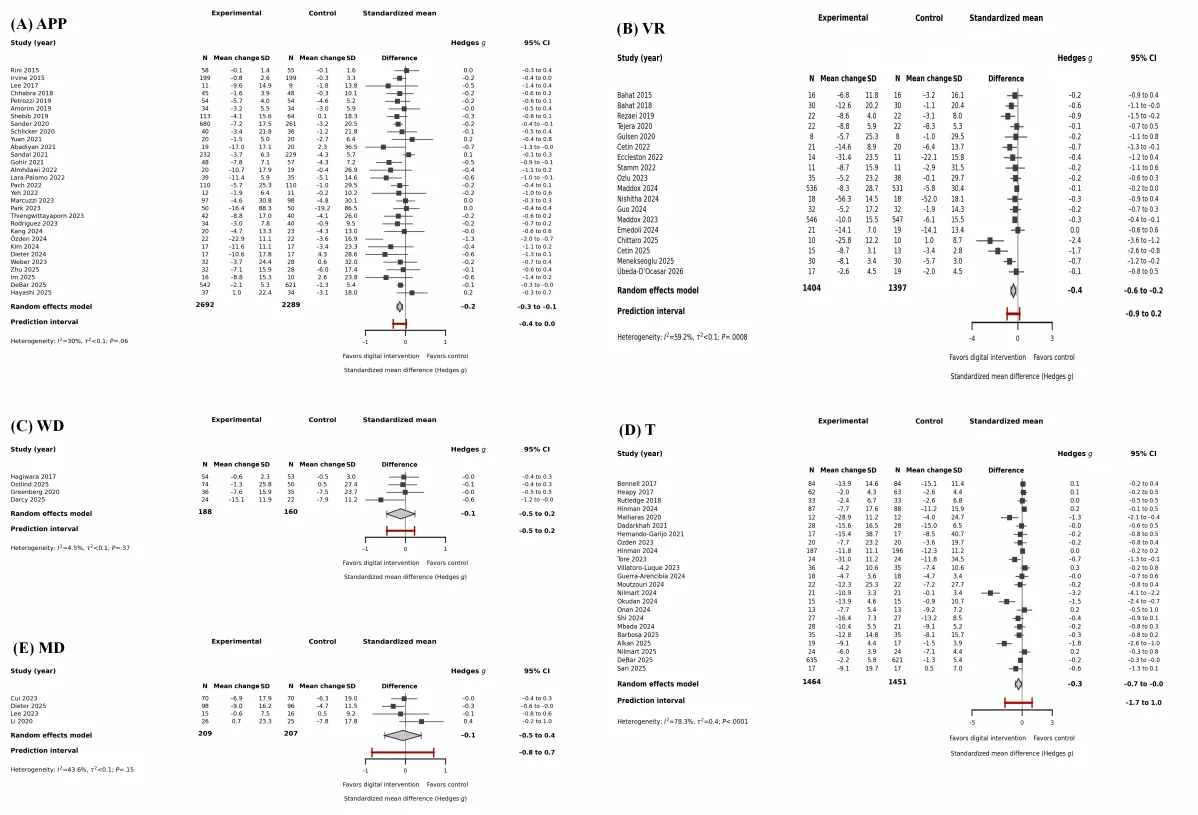
Forest plots of pairwise meta-analyses for functional disability outcomes across digital health intervention modalities. The plots present pooled effects of 5 categories of digital health interventions in patients with chronic musculoskeletal pain. Panels A-E correspond to app-based interventions (APP), virtual reality interventions (VR), wearable devices (WD), telehealth-based interventions (T), and multimodal digital interventions (MD). Random-effects models were used, and results are reported as Hedges *g* with 95% CIs. Squares represent individual study estimates (size proportional to weight), horizontal lines represent 95% CIs, diamonds represent pooled estimates, and red lines indicate prediction intervals. Negative values indicate greater improvement in functional disability, favoring digital interventions over the control condition [36-127].

### Bayesian Network Meta-Analysis

#### Pain Intensity

The network meta-analysis of pain intensity included 88 studies [36-70,72-81,83-94,96-110,112-127], comprising 12 digital health intervention nodes and 1 control node, for a total of 13 nodes, 13 direct comparisons, and 1 three-arm trial (Figure 5A). Model convergence was satisfactory, as indicated by potential scale reduction factors of 1 for all parameters; the DIC for the random-effects consistency model was 141.42 (Multimedia Appendix 10). Comparison-specific *I*² values for pairwise and network estimates are presented in Multimedia Appendix 11.

Compared with the control, 6 intervention nodes across the virtual reality, app-based, and telehealth-based categories yielded SMD estimates ranging from −0.55 to −0.23, with 95% CrIs excluding zero. Among active interventions, cognitive or perceptual virtual reality was associated with greater pain reduction than app-based cognitive behavioral therapy (SMD=−0.36, 95% CrI −0.66 to −0.07). Complete comparison-specific estimates are provided in the league table (Figure 6). Distraction virtual reality and motor function virtual reality had the highest SUCRA values (0.886 and 0.860, respectively). The complete ranking probabilities are shown in Figure 7A. However, these rankings should be interpreted cautiously given the between-study heterogeneity, wide CrIs, and low to very low certainty of evidence across comparisons. Node-splitting analyses did not identify statistically detectable inconsistency between direct and indirect evidence (all *P*>.05; Multimedia Appendix 12).

**Figure 5.**
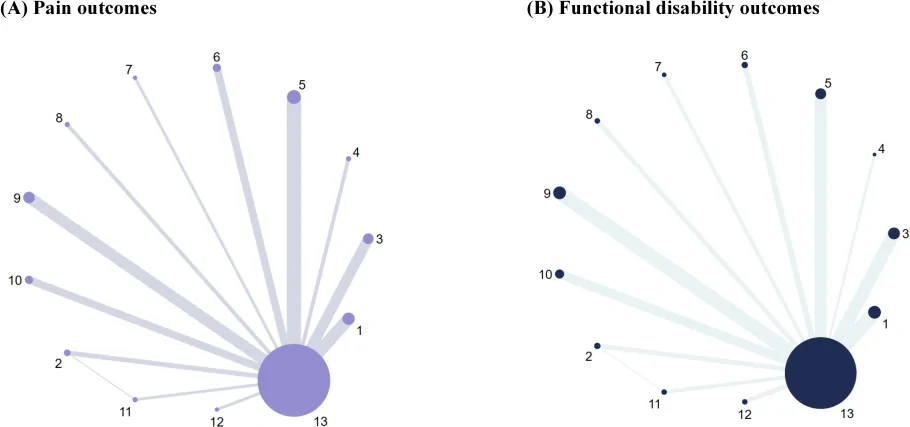
Network structure of digital health interventions for pain and functional disability outcomes. The network plots show the evidence structure of digital health interventions included in the network meta-analyses for pain outcomes and functional disability outcomes. Panel A presents the network for pain outcomes, and Panel B presents the network for functional disability outcomes. Each node represents an intervention category, and each connecting line represents a direct comparison between 2 interventions in the included randomized controlled trials. The size of each node is proportional to the number of participants receiving the corresponding intervention, and the thickness of each line reflects the number of direct comparisons between the 2 interventions. APP-CBT: app-based cognitive behavioral therapy; APP-Ex+CBT+Edu: app-based multimodal intervention combining exercise, cognitive behavioral therapy, and education; APP-Ex+Edu: app-based exercise plus education; APP-Exercise: app-based exercise; Control: control condition; CP-VR: cognitive/perceptual virtual reality; DA-VR: distraction virtual reality; MD: multimodal digital intervention; MF-VR: motor function virtual reality; Telehealth-CBT: telehealth-based cognitive behavioral therapy; Telehealth-E: telehealth-based exercise; Telehealth-EE: telehealth-based exercise plus education; WD: wearable devices.

**Figure 6.**
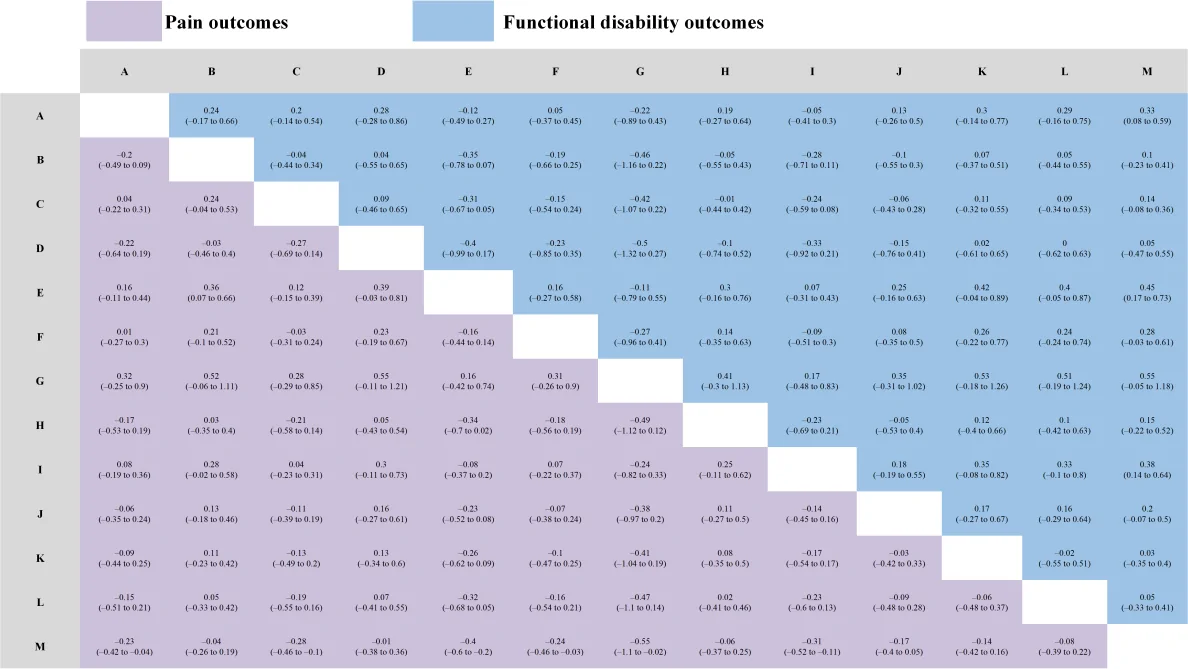
League table of the network meta-analysis for pain and functional disability outcomes, summarizing the comparative effects of digital health interventions in chronic musculoskeletal pain. The lower left triangle presents the results for pain outcomes, whereas the upper right triangle presents the results for functional disability outcomes. Each cell reports the posterior effect estimate and corresponding 95% credible interval for the comparison between 2 interventions. Positive values favor the column-defining intervention, whereas negative values favor the row-defining intervention. Purple shading indicates pain outcomes, and blue shading indicates functional disability outcomes. Darker shading represents larger absolute comparative effects. A-M indicate the intervention categories: A, app-based exercise; B, app-based cognitive behavioral therapy; C, app-based exercise plus education; D, app-based multimodal intervention combining exercise, cognitive behavioral therapy, and education; E, motor function virtual reality; F, cognitive/perceptual virtual reality; G, distraction virtual reality; H, wearable devices; I, telehealth-based exercise; J, telehealth-based exercise plus education; K, telehealth-based cognitive behavioral therapy; L, multimodal digital intervention; M, control condition.

**Figure 7.**
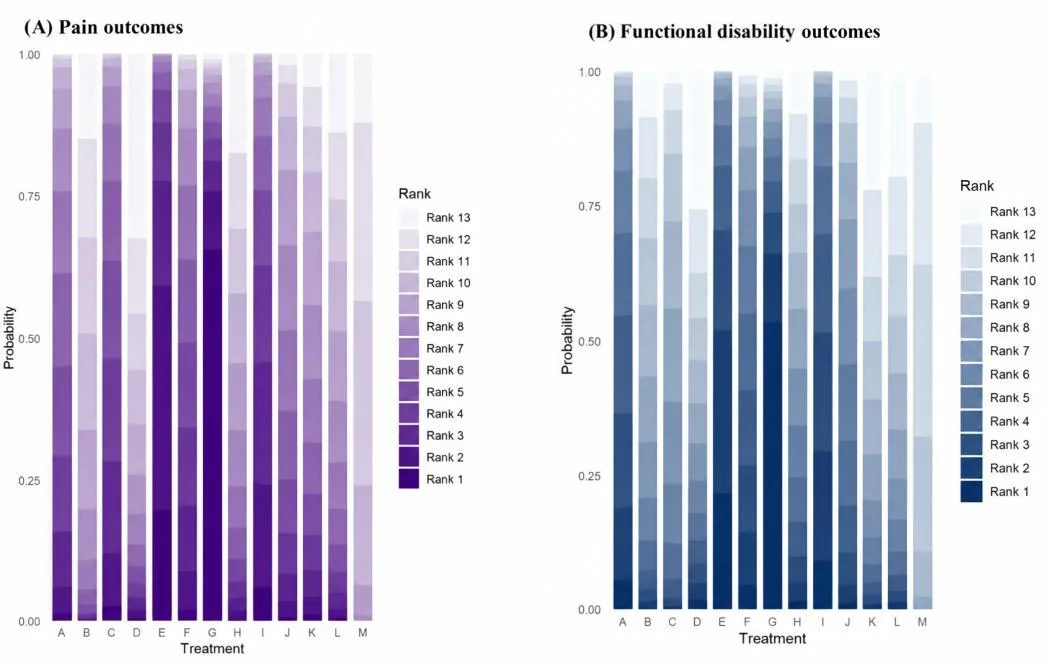
Ranking probabilities of digital health interventions for pain and functional disability outcomes. The stacked bar plots show the Bayesian ranking probabilities of digital health interventions for pain and functional disability outcomes. Panel A presents the ranking probabilities for pain outcomes, and Panel B presents those for functional disability outcomes. Each stacked bar represents 1 intervention and shows the probability of that intervention occupying each possible rank in the network meta-analysis. Darker shading indicates a higher probability of a better rank, with Rank 1 representing the most favorable intervention. After harmonization of outcome directions, better rankings indicate greater improvement in pain intensity or functional disability. A-M indicate the intervention categories: A, app-based exercise; B, app-based cognitive behavioral therapy; C, app-based exercise plus education; D, app-based multimodal intervention combining exercise, cognitive behavioral therapy, and education; E, motor function virtual reality; F, cognitive/perceptual virtual reality; G, distraction virtual reality; H, wearable devices; I, telehealth-based exercise; J, telehealth-based exercise plus education; K, telehealth-based cognitive behavioral therapy; L, multimodal digital intervention; M, control condition.

In the univariable network meta-regression, adding intervention duration improved the model fit (ΔDIC=−16.50), but the coefficient was imprecisely estimated, and its 95% CrI included zero (β=0.122, 95% CrI −0.003 to 0.255). No clear associations were found for risk of bias, participant age, control type, or outcome assessment time point (Table 2).

**Table 2.** Univariable network meta-regression analyses for pain outcomes.

Covariate (univariable model)	Interaction coefficient, B (95% CrI)	ΔDIC (adjusted − unadjusted)	Residual deviance (Dbar)	Between-study SD (95% CrI)
RoB 2 high risk	0.343 (−0.108 to 0.809)	−3.89	96.67	0.21 (0.13 to 0.30)
Intervention duration	0.122 (−0.003 to 0.255)	−16.50	97.09	0.20 (0.13 to 0.29)
Mean participant age	0.129 (−0.034 to 0.299)	−7.22	96.00	0.21 (0.13 to 0.30)
Control type	0.098 (−0.062 to 0.269)	−7.83	94.89	0.22 (0.14 to 0.31)
Outcome assessment time	0.028 (−0.113 to 0.172)	−1.17	96.67	0.22 (0.14 to 0.31)

a CrI: credible interval.

b DIC: deviance information criterion. ΔDIC was calculated as adjusted model DIC minus unadjusted model DIC; negative values indicate better model fit after covariate adjustment.

#### Functional Disability

The network meta-analysis for functional disability included 75 studies [36-51,53-56,58-69,72,74-77,80-86,88-90,92-94,96,97,99-103,105-110,112-114,116,117,119,120,122,123,125-127], forming the same 13-node structure (Figure 5B). Model convergence was satisfactory, with potential scale reduction factors of 1 for all parameters (Multimedia Appendix 10). Comparison-specific *I*^2^ values for pairwise and network estimates are presented in Multimedia Appendix 11.

Compared with control, motor function virtual reality, telehealth-based exercise, and app-based exercise yielded SMD estimates ranging from −0.45 to −0.33, with 95% CrIs excluding zero (Figure 6). Distraction virtual reality and motor function virtual reality had the highest SUCRA values (0.842 and 0.838, respectively), with complete ranking probabilities shown in Figure 7B. However, the estimate for distraction virtual reality was imprecise, and its 95% CrI included zero. Therefore, its high ranking should not be interpreted as definitive evidence of superiority. Node-splitting analyses did not identify statistically detectable inconsistency between direct and indirect evidence (all *P*>.05; Multimedia Appendix 12).

In the univariable network meta-regression, intervention duration was associated with treatment effects (β=0.19, 95% CrI 0.03‐0.38) and improved model fit (ΔDIC=−38.20). This systematic review–level association should nevertheless be considered exploratory. No stable associations were observed for risk of bias, participant age, control type, or outcome assessment time point (Table 3).

**Table 3.** Univariable network meta-regression analyses for functional disability outcomes.

Covariate (univariable model)	Interaction coefficient, B (95% CrI)^a^	ΔDIC^b^ (adjusted − unadjusted)	Residual deviance (Dbar)	Between-study SD (95% CrI)
RoB 2 high risk	0.008 (−0.186 to 0.206)	−0.02	101.05	0.30 (0.17 to 0.43)
Intervention duration	0.197 (0.032 to 0.375)	−38.20	105.76	0.25 (0.13 to 0.39)
Mean participant age	0.171 (−0.043 to 0.394)	−10.47	101.33	0.28 (0.16 to 0.42)
Control type	0.008 (−0.186 to 0.206)	−0.02	101.05	0.3 (0.17 to 0.43)
Outcome assessment time	0.104 (−0.079 to 0.300)	−24.25	99.76	0.30 (0.18 to 0.43)

a CrI: credible interval.

b DIC: deviance information criterion. ΔDIC was calculated as adjusted model DIC minus unadjusted model DIC; negative values indicate better model fit after covariate adjustment.

## Discussion

### Principal Findings

This systematic review and Bayesian network meta-analysis included 92 randomized controlled trials involving 12,595 participants with CMP. Overall, DHIs were associated with small to moderate average improvements in pain and functional disability. App-based, virtual reality–based, and telerehabilitation interventions showed more favorable average effects, whereas the estimates for wearable device and multimodal interventions were less precise. Subgroup analyses suggested that interventions incorporating exercise or motor function training tended to produce larger effect estimates than those centered primarily on education or cognitive behavioral therapy. However, several subgroups contained relatively few studies; therefore, these patterns should be considered exploratory rather than evidence of component-level superiority. In the network meta-analysis, motor function–oriented virtual reality and exercise-based telerehabilitation showed potentially favorable effects across pain and disability outcomes. Some attention- or cognition-oriented interventions ranked relatively highly for pain, but their effects on functional disability were less certain.

In the pairwise meta-analyses, the pooled estimates and their 95% CIs represent average effects across the included studies, whereas the 95% prediction intervals indicate the range of true effects that might be expected in a new, broadly comparable study or clinical setting. App-based, virtual reality–based, and telerehabilitation interventions showed favorable average effects on pain and disability, with 95% CIs excluding the null. However, the corresponding prediction intervals crossed the null, indicating that effects may be smaller, absent, or potentially different across populations, intervention protocols, and health care settings. Prediction intervals therefore describe variation in study-level effects across settings rather than variation in individual patient responses [128].

Variation in effects was observed across intervention modalities and outcomes. Accordingly, *I*^2^ values were interpreted descriptively rather than being used to classify the magnitude of heterogeneity, with greater emphasis placed on *τ*² and prediction intervals. This variation may reflect differences in musculoskeletal conditions, baseline severity, intervention intensity and duration, professional supervision, comparator content, and assessment timing. The included interventions also varied in their structure and implementation. App-based exercise programs differed in training intensity, frequency, and feedback delivery [49,55,68]; virtual reality interventions differed in immersion, therapeutic content, and interaction modality [60,85,116]; and telerehabilitation programs varied in supervision, real-time interaction, and follow-up frequency [65,82]. Although leave-one-out analyses did not materially alter the direction of the pooled estimates, robustness to the exclusion of individual studies does not rule out variation in the magnitude or applicability of effects across settings.

In the Bayesian network meta-analysis, 95% CrIs describe uncertainty around the average relative effects between intervention nodes. Although several nodes had 95% CrIs excluding the null, some estimates remained imprecise and several comparisons were supported by limited direct evidence. Moreover, the absence of statistically detectable inconsistency between direct and indirect evidence does not eliminate heterogeneity or ensure stable treatment rankings. SUCRA values summarize relative ranking probabilities and should therefore be interpreted together with the corresponding effect estimates, 95% CrIs, network structure, and certainty of evidence rather than as a definitive clinical hierarchy [129]. Consequently, the relatively high rankings of some virtual reality– and exercise-based interventions suggest that they may warrant further evaluation but do not establish their superiority over other digital interventions. These findings should also be interpreted in light of risk of bias and certainty of evidence. Overall, 72.8% of the included studies were judged to have some concerns using RoB 2. Pain and functional disability were predominantly participant reported, and participant blinding was generally infeasible for digital behavioral and rehabilitation interventions. Awareness of treatment allocation may therefore have influenced participant expectations, engagement, or outcome reporting [130]. Together with heterogeneity and imprecision, these considerations contributed to the predominantly low or very low certainty ratings under the GRADE approach adapted for network meta-analysis [33,131]. Accordingly, the favorable average effects should be interpreted as suggesting potential benefits rather than as establishing clinically important improvement or comparative superiority [132]. Network meta-regression suggested a potential study-level association between intervention duration and functional disability, whereas no clear association was observed for pain. This finding may be consistent with functional improvement requiring sustained training, but it should not be interpreted as evidence of a causal mechanism because intervention duration may be associated with treatment intensity, adherence, supervision, or other study characteristics. Overall, by jointly considering digital delivery modality and core therapeutic content, this systematic review provides a more clinically interpretable framework for comparing digital interventions. The findings suggest that therapeutic content and delivery format should be considered together when designing interventions and selecting candidates for future head-to-head trials.

### Comparison with Previous Research and Contribution to the Field

Digital health modalities may exert their effects through multiple pathways, including behavioral regulation, cognitive reinforcement, and motor learning [133]. App-based and telerehabilitation interventions may facilitate self-management by delivering structured exercise and supporting goal setting, feedback, and professional contact [134]. The mechanisms of virtual reality may vary according to its therapeutic purpose: distraction-based virtual reality may modulate pain perception through attentional distraction, whereas motor function–oriented virtual reality may promote graded movement and motor relearning [135]. Wearable devices primarily enable behavioral monitoring and feedback [136], whereas multimodal interventions integrate multiple delivery formats or therapeutic components and vary substantially in composition [137]. Taken together, these differences suggest that the effects of digital interventions may be shaped jointly by the delivery modality and therapeutic content. Previous reviews have addressed complementary but more focused research questions. Some reviews synthesized the overall effects of DHIs, whereas others focused on mobile health, virtual reality, remote rehabilitation, or economic outcomes [11,12,14,16]. An earlier network meta-analysis compared electronic delivery modalities but did not differentiate the therapeutic components delivered within each modality [20]. Thus, previous reviews support the potential value of digital care, but comparative evidence across a broad range of delivery modality–therapeutic component combinations remains limited.

This systematic review extends the existing evidence by synthesizing 92 randomized controlled trials within a 2-level framework integrating digital delivery modality and core therapeutic content. Pairwise meta-analyses estimated effects at the broader modality level, whereas the Bayesian network meta-analysis compared 12 active intervention nodes defined by specific delivery modality–therapeutic component combinations. This approach distinguishes interventions delivered through the same technology but incorporating different therapeutic components while enabling comparisons of similar components delivered through different platforms. Rather than isolating the effects of individual components, the framework compares clinically meaningful delivery modality–therapeutic component combinations as implemented in the included trials. It therefore provides a more clinically interpretable classification, may improve the interpretation of within-modality heterogeneity, and identifies combinations that warrant further evaluation in head-to-head trials.

### Implications for Practice and Research

This systematic review suggests that DHIs may have clinical value in the management of CMP, although their effects may vary across populations, intervention protocols, and health care settings. Clinical decisions should therefore consider the therapeutic content delivered as well as the digital platform, rather than relying on effect estimates or treatment rankings alone. App-based and telerehabilitation interventions may offer flexible delivery, but their implementation depends on sustained engagement, digital literacy, access to suitable devices and internet services, professional support, and integration into clinical workflows [138,139]. Virtual reality may be useful for selected patients but may require additional equipment, technical support, training, and implementation resources [140]. Because adherence, costs, and implementation outcomes were not consistently reported across the included trials, the relative feasibility and cost-effectiveness of these approaches remain uncertain. In practice, intervention selection should therefore be guided by patient preferences, accessibility, supervision needs, and locally available resources. Future head-to-head trials should clearly report the digital platform, core therapeutic components, intervention dose, professional involvement, adherence, adverse events, resource use, and implementation outcomes [141]. Standardized outcome measures and prespecified follow-up periods are also needed to improve comparability and determine whether effects are sustained over time [142].

### Strengths and Limitations

This systematic review has several strengths, including the joint classification of DHIs by delivery modality and therapeutic content, the complementary use of pairwise and Bayesian network meta-analyses, and the assessment of risk of bias and certainty of evidence using RoB 2 and the GRADE approach adapted for network meta-analysis, respectively.

Several limitations should nevertheless be acknowledged. First, clinical and methodological variation across studies, including differences in musculoskeletal conditions, intervention protocols, comparators, and assessment timing, may have contributed to heterogeneity and influenced some indirect comparisons [46,77]. Second, pain and disability were predominantly participant reported, and participant blinding was generally infeasible. Potential expectation or reporting effects, together with heterogeneity and imprecision, contributed to the generally low or very low certainty of evidence. Third, the final assessment was selected to evaluate the longest available effect while avoiding correlated estimates; although follow-up duration varied, its potential influence was explored through network meta-regression. Fourth, some nodes and active treatment comparisons were supported by relatively few studies, resulting in imprecise estimates and uncertainty in SUCRA rankings [132]. Rankings should therefore be interpreted alongside the effect estimates, uncertainty intervals, network structure, and GRADE certainty ratings [143]. Fifth, the use of SMDs facilitated the synthesis of different outcome measures but limited direct interpretation against a common threshold for clinical importance. Finally, gray literature and trial registries were not systematically searched; therefore, publication bias cannot be excluded, particularly for comparisons supported by few studies.

### Conclusions

This systematic review and Bayesian network meta-analysis of 92 randomized controlled trials suggests that DHIs may provide small to moderate average improvements in pain and functional disability among people with CMP. Interventions incorporating exercise or motor function training through apps, virtual reality, or telerehabilitation showed potentially favorable patterns across outcomes. However, between-study variability in effect estimates, some risk-of-bias concerns, prediction intervals crossing the null for many comparisons, and predominantly low or very low certainty of evidence limit confidence in the comparative estimates. The SUCRA rankings should therefore be regarded as exploratory and should not be interpreted as establishing the superiority of any intervention. The principal innovation of this systematic review is the joint classification of interventions according to digital delivery modality and core therapeutic content. Unlike previous reviews that primarily evaluated overall effects, individual platforms, or broad delivery modalities, this framework compares clinically meaningful modality-component combinations within a common analytical structure. It provides the field with a more clinically interpretable classification and comparative evidence base for intervention design and future head-to-head research. In real-world practice, the framework may help clinicians and health care planners consider therapeutic content alongside accessibility, digital literacy, adherence, supervision requirements, costs, and available resources when selecting digital interventions. Adequately powered head-to-head trials with standardized outcomes and implementation and economic evaluations are needed to confirm comparative effectiveness and determine whether the observed benefits are sustained across health care settings.

## Supplementary material

10.2196/95903 Multimedia Appendix 1 Database search strategies and search results.

10.2196/95903 Multimedia Appendix 2 Classification, coding scheme, and operational definitions of digital health interventions and control conditions used for subgroup analyses and Bayesian network meta-analysis.

10.2196/95903 Multimedia Appendix 3 Definitions, measurement hierarchy, and effect measures for pain intensity, functional disability, and health-related quality of life outcomes.

10.2196/95903 Multimedia Appendix 4 Characteristics of the included randomized controlled trials.

10.2196/95903 Multimedia Appendix 5 Descriptive synthesis of health-related quality of-life outcomes.

10.2196/95903 Multimedia Appendix 6 Risk of bias assessment for included trials, including individually randomized trials and cluster randomized trials.

10.2196/95903 Multimedia Appendix 7 Subgroup analyses across 3 moderators—application method, treatment method, and virtual reality.

10.2196/95903 Multimedia Appendix 8 Funnel plots assessing publication bias across the included studies.

10.2196/95903 Multimedia Appendix 9 Sensitivity analyses for pain and functional disability outcomes, showing Hedges *g* with 95% CI and prediction intervals.

10.2196/95903 Multimedia Appendix 10 Convergence diagnostics of the Bayesian network meta-analysis model, including trace plots, posterior density plots, and Gelman-Rubin statistics.

10.2196/95903 Multimedia Appendix 11 Forest plots of the Bayesian network meta-analysis, showing standardized mean differences with 95% credible intervals.

10.2196/95903 Multimedia Appendix 12 Node-splitting analyses assessing inconsistency between direct and indirect evidence in the network meta-analysis for pain and functional disability outcomes.

10.2196/95903 Checklist 1 PRISMA (Preferred Reporting Items for Systematic Reviews and Meta-Analyses) checklist.

## Abbreviations

CMP: chronic musculoskeletal pain
CrI: credible interval
DHI: digital health intervention
DIC: deviance information criterion
GRADE: Grading of Recommendations Assessment, Development and Evaluation
ICD-11: International Classification of Diseases, 11th Revision
MCMC: Markov chain Monte Carlo
NMA: network meta-analysis
NRS: numeric rating scale
PICOS: population, intervention, comparison, outcome, and study design
PRISMA: Preferred Reporting Items for Systematic Reviews and Meta-Analyses
PRISMA-NMA: Preferred Reporting Items for Systematic Reviews and Meta-Analyses extension for Network Meta-Analyses
PRISMA-S: Preferred Reporting Items for Systematic reviews and Meta-Analyses literature search extension
SUCRA: surface under the cumulative ranking curve
VAS: visual analogue scale
