# Supplementary material for: Effects of Digital Health Interventions in Chronic Musculoskeletal Pain: Systematic Review and Bayesian Network Meta-Analysis of Randomized Controlled Trials
Source: J Med Internet Res. 2026 Oct 7;28:e95903. doi: 10.2196/95903 (PMC13645338; doi:10.2196/95903)
Supplement: Supplementary file 2 [file jmir-v28-e95903-s001.docx]

**Multimedia Appendix 1. Database search strategies and search results.**

**Search overview**

The initial systematic literature search was conducted across PubMed, CINAHL, Web of Science, MEDLINE, Scopus, Embase, and the Cochrane Library from database inception to July 10, 2025. Before manuscript submission, an updated search was conducted to identify newly published eligible studies. The updated search covered the period from July 11, 2025, to January 29, 2026, and applied the same databases, eligibility criteria, and core search concepts as the initial search. Database-specific syntax and controlled vocabulary were adapted as appropriate for each platform. The complete search strategies and numbers of records retrieved are presented below.

**eTable 1. Search strategies used in PubMed, CINAHL, Web of Science, MEDLINE, Scopus, Embase, and the Cochrane Library from database inception to July 10, 2025**

| **Database** | **Number of Records Retrieved** |
| --- | --- |
| PubMed | 1,543 |
| CINAHL | 1,737 |
| WOS | 1,319 |
| MEDLINE | 982 |
| Scopus | 846 |
| Embase | 1,379 |
| Cochrane | 710 |
| Total | 8,516 |

| **PubMed** | | |
| --- | --- | --- |
| Step | Search Strategy | Number of Records Retrieved |
| #1 | "Musculoskeletal Pain"[Mesh] OR "Chronic Pain"[Mesh] OR "Back Pain"[Mesh] OR "Neck Pain"[Mesh] OR "Shoulder Pain"[Mesh] OR "Myofascial Pain Syndromes"[Mesh] OR "Fibromyalgia"[Mesh] OR "Osteoarthritis"[Mesh] OR "Arthralgia"[Mesh] OR "Pain, Intractable"[Mesh] | 203016 |
| #2 | musculoskeletal pain[Title] OR chronic pain[Title] OR chronic musculoskeletal pain[Title] OR neck pain[Title] OR back pain[Title] OR low back pain[Title] OR shoulder pain[Title] OR knee pain[Title] OR fibromyalgia[Title] OR myofascial pain[Title] OR muscle pain[Title] OR joint pain[Title] OR arthralgia[Title] OR musculoskeletal disorder*[Title] OR lower limb pain[Title] OR upper limb pain[Title] OR persistent pain[Title] OR long-term pain[Title] | 75367 |
| #3 | #1 OR #2 | 226715 |
| #4 | "Mobile Applications"[Mesh] | 14978 |
| #5 | Mobile Applications[Title] OR mhealth[Title] OR m-health[Title] OR mobile health[Title] OR smartphone*[Title] OR app[Title] OR application*[Title] OR mobile app[Title] OR digital health[Title] OR self-management app[Title] OR mobile-based intervention[Title] OR smartphone application[Title] | 414867 |
| #6 | #4 OR #5 | 420609 |
| #7 | "Virtual Reality"[Mesh] OR "Virtual Reality Exposure Therapy"[Mesh] | 9461 |
| #8 | virtual reality[Title] OR Virtual Reality Exposure Therapy[Title] OR VR[Title] OR virtual reality simulator[Title] OR virtual reality system[Title] OR immersive virtual reality[Title] OR VR headset[Title] OR virtual environment[Title] OR head-mounted display[Title] OR 3D simulation[Title] OR interactive virtual system[Title] | 13795 |
| #9 | #7 OR #8 | 17058 |
| #10 | "Telerehabilitation"[Mesh] | 1443 |
| #11 | remote rehabilitation[Title] OR telerehabilitation[Title] OR virtual reality head-mounted display[Title] | 1739 |
| #12 | #10 OR #11 | 2268 |
| #13 | "Wearable Electronic Devices"[Mesh] | 23431 |
| #14 | wearable[Title] OR wearable device[Title] OR wearable sensor[Title] OR body-worn[Title] OR tracker*[Title] OR monitor*[Title] OR wristband[Title] OR smartwatch[Title] OR pedometer*[Title] OR accelerom*[Title] OR actigraph*[Title] OR actiwatch[Title] OR actimeter[Title] OR posture sensor*[Title] OR posture monitor*[Title] OR fitbit*[Title] OR Apple Watch[Title] OR Withings[Title] OR Garmin[Title] OR Pebble[Title] OR Jawbone[Title] OR SenseWear[Title] OR GT3X[Title] OR "body media"[Title] OR Samsung[Title] | 194329 |
| #15 | #13 OR #14 | 209647 |
| #16 | "Cognitive Behavioral Therapy"[MeSH Terms] | 41205 |
| #17 | "iCBT"[Title] OR "i-CBT"[Title] OR "cCBT"[Title] OR "computerized cognitive behavioural therapy"[Title] OR ePsych*[Title] OR "e-Psych*"[Title] | 127 |
| #18 | #16 OR #17 | 41271 |
| #19 | randomized controlled trial[Title/Abstract] OR controlled clinical trial[Title/Abstract] OR randomized[Title/Abstract] OR randomly[Title/Abstract] OR trial[Title/Abstract] OR clinical trial[Title/Abstract] | 1608999 |
| #20 | #6 OR #9 OR #12 OR 15 OR #18 | 680711 |
| #21 | #20 AND #3 | 4515 |
| #22 | #21 AND #19 | 1543 |

| **CINAHL** | | |
| --- | --- | --- |
| Step | Search Strategy | Number of Records Retrieved |
| S1 | [MH musculoskeletal pain OR MH chronic pain OR MH back pain OR MH neck pain OR MH shoulder pain](https://libdb.csu.edu.cn/search/results?db=c8h&expanders=concept&limiters=None&q=MH%20musculoskeletal%20pain%20OR%20MH%20chronic%20pain%20OR%20MH%20back%20pain%20OR%20MH%20neck%20pain%20OR%20MH%20shoulder%20pain&qm=W3sidmFsdWUiOiJtdXNjdWxvc2tlbGV0YWwgcGFpbiIsInR5cGUiOiJmaWVsZCIsImNvZGUiOiJNSCJ9LHsidmFsdWUiOiJPUiIsInR5cGUiOiJsb2dpYyJ9LHsidmFsdWUiOiJjaHJvbmljIHBhaW4iLCJ0eXBlIjoiZmllbGQiLCJjb2RlIjoiTUgifSx7InZhbHVlIjoiT1IiLCJ0eXBlIjoibG9naWMifSx7InZhbHVlIjoiYmFjayBwYWluIiwidHlwZSI6ImZpZWxkIiwiY29kZSI6Ik1IIn0seyJ2YWx1ZSI6Ik9SIiwidHlwZSI6ImxvZ2ljIn0seyJ2YWx1ZSI6Im5lY2sgcGFpbiIsInR5cGUiOiJmaWVsZCIsImNvZGUiOiJNSCJ9LHsidmFsdWUiOiJPUiIsInR5cGUiOiJsb2dpYyJ9LHsidmFsdWUiOiJzaG91bGRlciBwYWluIiwidHlwZSI6ImZpZWxkIiwiY29kZSI6Ik1IIn1d&searchMode=enhanced&sort=relevance&userDirectAction=true&isDashboardExpanded=true) | 49,735 |
| S2 | [MH osteoarthritis OR MH arthralgia OR MH pain intractable OR MH fibromyalgia OR MH myofascial pain syndrome](https://libdb.csu.edu.cn/search/results?db=c8h&expanders=concept&limiters=None&q=MH%20osteoarthritis%20OR%20MH%20arthralgia%20OR%20MH%20pain%20intractable%20OR%20MH%20fibromyalgia%20OR%20MH%20myofascial%20pain%20syndrome&qm=W3sidmFsdWUiOiJvc3Rlb2FydGhyaXRpcyIsInR5cGUiOiJmaWVsZCIsImNvZGUiOiJNSCJ9LHsidmFsdWUiOiJPUiIsInR5cGUiOiJsb2dpYyJ9LHsidmFsdWUiOiJhcnRocmFsZ2lhIiwidHlwZSI6ImZpZWxkIiwiY29kZSI6Ik1IIn0seyJ2YWx1ZSI6Ik9SIiwidHlwZSI6ImxvZ2ljIn0seyJ2YWx1ZSI6InBhaW4gaW50cmFjdGFibGUiLCJ0eXBlIjoiZmllbGQiLCJjb2RlIjoiTUgifSx7InZhbHVlIjoiT1IiLCJ0eXBlIjoibG9naWMifSx7InZhbHVlIjoiZmlicm9teWFsZ2lhIiwidHlwZSI6ImZpZWxkIiwiY29kZSI6Ik1IIn0seyJ2YWx1ZSI6Ik9SIiwidHlwZSI6ImxvZ2ljIn0seyJ2YWx1ZSI6Im15b2Zhc2NpYWwgcGFpbiBzeW5kcm9tZSIsInR5cGUiOiJmaWVsZCIsImNvZGUiOiJNSCJ9XQ==&searchMode=enhanced&sort=relevance&userDirectAction=true&isDashboardExpanded=true) | 26,067 |
| S3 | [S2 OR S1](https://libdb.csu.edu.cn/search/results?combinedSearchQueryId=sq:0bf21218-9b1c-4332-b8da-064bcd0c6e39&db=c8h&expanders=concept&limiters=None&q=S2%20OR%20S1&searchMode=enhanced&sort=relevance&userDirectAction=true&isDashboardExpanded=true) | 73,999 |
| S4 | XB musculoskeletal pain OR chronic pain OR chronic musculoskeletal pain OR neck pain OR back pain OR low back pain OR shoulder pain OR knee pain OR fibromyalgia OR myofascial pain OR muscle pain OR joint pain OR arthralgia OR musculoskeletal disorder* OR lower limb pain OR upper limb pain OR persistent pain OR long-term pain | 91,796 |
| S5 | [S4 OR S3](https://libdb.csu.edu.cn/search/results?combinedSearchQueryId=sq:e2048439-ef69-438f-9d70-7663a718c034&db=c8h&expanders=concept&limiters=None&q=S4%20OR%20S3&searchMode=enhanced&sort=relevance&userDirectAction=true&isDashboardExpanded=true) | 128,535 |
| S6 | MH mobile applications OR XB (Mobile Applications OR mhealth OR m-health OR mobile health OR smartphone* OR app OR application* OR mobile app OR digital health OR self-management app OR mobile-based intervention OR smartphone application) | 208,056 |
| S7 | MH virtual reality OR XB (virtual reality OR Virtual Reality Exposure Therapy OR VR OR virtual reality simulator OR virtual reality system OR immersive virtual reality OR VR headset OR virtual environment OR head-mounted display OR 3D simulation OR interactive virtual system) OR MH virtual reality exposure therapy | 13,602 |
| S8 | [MH telerehabilitation OR XB (remote rehabilitation OR telerehabilitation OR virtual reality head-mounted display)](https://libdb.csu.edu.cn/search/results?db=c8h&expanders=concept&limiters=None&q=MH%20telerehabilitation%20AND%20XB%20(remote%20rehabilitation%20OR%20telerehabilitation%20OR%20virtual%20reality%20head-mounted%20display)&qm=W3sidmFsdWUiOiJ0ZWxlcmVoYWJpbGl0YXRpb24iLCJ0eXBlIjoiZmllbGQiLCJjb2RlIjoiTUgifSx7InZhbHVlIjoiQU5EIiwidHlwZSI6ImxvZ2ljIn0seyJ2YWx1ZSI6InJlbW90ZSByZWhhYmlsaXRhdGlvbiBPUiB0ZWxlcmVoYWJpbGl0YXRpb24gT1IgdmlydHVhbCByZWFsaXR5IGhlYWQtbW91bnRlZCBkaXNwbGF5IiwidHlwZSI6ImZpZWxkIiwiY29kZSI6IlhCIn1d&searchMode=enhanced&sort=relevance&userDirectAction=true&isDashboardExpanded=true) | 1914 |
| S9 | MH wearable electronic devices OR XB (wearable OR "wearable device" OR "wearable sensor" OR body-worn OR tracker* OR monitor* OR wristband OR smartwatch OR pedometer* OR accelerom* OR actigraph* OR actiwatch OR actimeter OR posture sensor* OR posture monitor* OR fitbit* OR "Apple Watch" OR Withings OR Garmin OR Pebble OR Jawbone OR SenseWear OR GT3X OR "body media" OR Samsung) | 208,752 |
| S10 | MH internet-based cognitive behavioural therapy OR XB (iCBT OR "i-CBT" OR cCBT OR "c-CBT" OR "electronic cognitive behavioural therapy" OR "computerized cognitive behavioural therapy" OR "electronic psychotherapy" OR ePsych* OR "e-Psych*") | 593 |
| S11 | [MH Randomized Controlled Trial OR XB (randomized controlled trial OR controlled clinical trial OR randomized OR randomly OR trial OR clinical trial)](https://libdb.csu.edu.cn/search/results?db=c8h&expanders=concept&limiters=None&q=MH%20Randomized%20Controlled%20Trial%20OR%20XB%20(randomized%20controlled%20trial%20OR%20controlled%20clinical%20trial%20OR%20randomized%20OR%20randomly%20OR%20trial%20OR%20clinical%20trial)&qm=W3sidmFsdWUiOiJSYW5kb21pemVkIENvbnRyb2xsZWQgVHJpYWwiLCJ0eXBlIjoiZmllbGQiLCJjb2RlIjoiTUgifSx7InZhbHVlIjoiT1IiLCJ0eXBlIjoibG9naWMifSx7InZhbHVlIjoicmFuZG9taXplZCBjb250cm9sbGVkIHRyaWFsIE9SIGNvbnRyb2xsZWQgY2xpbmljYWwgdHJpYWwgT1IgcmFuZG9taXplZCBPUiByYW5kb21seSBPUiB0cmlhbCBPUiBjbGluaWNhbCB0cmlhbCIsInR5cGUiOiJmaWVsZCIsImNvZGUiOiJYQiJ9XQ==&searchMode=enhanced&sort=relevance&userDirectAction=true&isDashboardExpanded=true) | 588,324 |
| S12 | [S6 OR S7 OR S8 OR S9 OR S1](https://libdb.csu.edu.cn/search/results?combinedSearchQueryId=sq:99be1762-41e0-4319-aed2-1c4f00cd9ac5&db=c8h&expanders=concept&limiters=None&q=S6%20OR%20S7%20OR%20S9%20OR%20S10%20OR%20S11&searchMode=enhanced&sort=relevance&userDirectAction=true&isDashboardExpanded=true)0 | 415,975 |
| S13 | [S11 AND S12 AND S5](https://libdb.csu.edu.cn/search/results?combinedSearchQueryId=sq:1b9bae82-2252-40b5-b296-a0e147e68c6b&db=c8h&expanders=concept&limiters=None&q=S13%20AND%20S12%20AND%20S5&searchMode=enhanced&sort=relevance&userDirectAction=true&isDashboardExpanded=true) | 1737 |

| **Web of Science** | | |
| --- | --- | --- |
| Step | Search Strategy | Number of Records Retrieved |
| #1 | TI=(musculoskeletal pain OR chronic pain OR chronic musculoskeletal pain OR neck pain OR back pain OR low back pain OR shoulder pain OR knee pain OR fibromyalgia OR myofascial pain OR muscle pain OR joint pain OR arthralgia OR musculoskeletal disorder* OR lower limb pain OR upper limb pain OR persistent pain OR long-term pain) and Preprint Citation Index (Exclude – Database) | 158416 |
| #2 | ((((TS=(Mobile Applications OR mhealth OR m-health OR mobile health OR smartphone* OR app OR application* OR mobile app OR digital health OR self-management app OR mobile-based intervention OR smartphone application)) OR TS=(virtual reality OR Virtual Reality Exposure Therapy OR VR OR virtual reality simulator OR virtual reality system OR immersive virtual reality OR VR headset OR virtual environment OR head-mounted display OR 3D simulation OR interactive virtual system)) OR TS=(remote rehabilitation OR telerehabilitation OR "virtual reality head-mounted display")) OR TS=(wearable OR "wearable device" OR "wearable sensor" OR body-worn OR tracker* OR monitor* OR wristband OR smartwatch OR pedometer* OR accelerom* OR actigraph* OR actiwatch OR actimeter OR posture sensor* OR posture monitor* OR fitbit* OR "Apple Watch" OR Withings OR Garmin OR Pebble OR Jawbone OR SenseWear OR GT3X OR "body media" OR Samsung)) OR TS=(“iCBT” OR "i-CBT" OR “cCBT” OR "computerized cognitive behavioural therapy" OR ePsych* OR "e-Psych*") and Preprint Citation Index (Exclude – Database) | 17093383 |
| #3 | TI=(randomized controlled trial OR controlled clinical trial OR randomized OR randomly OR trial OR clinical trial) and Preprint Citation Index (Exclude – Database) | 825380 |
| #4 | #1 AND #2 AND #3 and Preprint Citation Index (Exclude – Database) | 1319 |

| **MEDLINE** | | |
| --- | --- | --- |
| Step | Search Strategy | Number of Records Retrieved |
| #1 | TI=(musculoskeletal pain OR chronic pain OR chronic musculoskeletal pain OR neck pain OR back pain OR low back pain OR shoulder pain OR knee pain OR fibromyalgia OR myofascial pain OR muscle pain OR joint pain OR arthralgia OR musculoskeletal disorder* OR lower limb pain OR upper limb pain OR persistent pain OR long-term pain) | 100171 |
| #2 | ((((TS=(Mobile Applications OR mhealth OR m-health OR mobile health OR smartphone* OR app OR application* OR mobile app OR digital health OR self-management app OR mobile-based intervention OR smartphone application)) OR TS=(virtual reality OR Virtual Reality Exposure Therapy OR VR OR virtual reality simulator OR virtual reality system OR immersive virtual reality OR VR headset OR virtual environment OR head-mounted display OR 3D simulation OR interactive virtual system)) OR TS=(remote rehabilitation OR telerehabilitation OR "virtual reality head-mounted display")) OR TS=(wearable OR "wearable device" OR "wearable sensor" OR body-worn OR tracker* OR monitor* OR wristband OR smartwatch OR pedometer* OR accelerom* OR actigraph* OR actiwatch OR actimeter OR posture sensor* OR posture monitor* OR fitbit* OR "Apple Watch" OR Withings OR Garmin OR Pebble OR Jawbone OR SenseWear OR GT3X OR "body media" OR Samsung)) OR TS=(“iCBT” OR "i-CBT" OR “cCBT” OR "computerized cognitive behavioural therapy" OR ePsych* OR "e-Psych*") | 3329841 |
| #3 | TI=(randomized controlled trial OR controlled clinical trial OR randomized OR randomly OR trial OR clinical trial) | 513566 |
| #4 | #1 AND #2 AND #3 | 982 |

| **SCOPUS** | | |
| --- | --- | --- |
| Step | Search Strategy | Number of Records Retrieved |
| #1 | TITLE-ABS-KEY ( ( "musculoskeletal pain" OR "chronic pain" OR "chronic musculoskeletal pain" OR "neck pain" OR "back pain" OR "low back pain" OR "shoulder pain" OR "knee pain" OR fibromyalgia OR "myofascial pain" OR "muscle pain" OR "joint pain" OR arthralgia OR "musculoskeletal disorder*" OR "lower limb pain" OR "upper limb pain" OR "persistent pain" OR "long-term pain" OR "intractable pain" ) ) | 432,110 |
| #2 | TITLE-ABS-KEY ( ( "mobile applications" OR mhealth OR "mobile health" OR smartphone* OR app OR application* OR "mobile app" OR "digital health" OR "self-management app" OR "mobile-based intervention" OR "smartphone application" OR "virtual reality" OR "virtual reality exposure therapy" OR vr OR "virtual reality simulator" OR "virtual reality system" OR "immersive virtual reality" OR "VR headset" OR "virtual environment" OR "head-mounted display" OR "3D simulation" OR "interactive virtual system" OR "remote rehabilitation" OR telerehabilitation OR "virtual reality head-mounted display" OR wearable OR "wearable device" OR "wearable sensor" OR "body-worn" OR tracker* OR monitor* OR wristband OR smartwatch OR pedometer* OR accelerom* OR actigraph* OR actiwatch OR actimeter OR "posture sensor*" OR "posture monitor*" OR fitbit* OR "apple watch" OR withings OR garmin OR pebble OR jawbone OR sensewear OR gt3x OR "body media" OR samsung OR icbt OR i-cbt OR epsych* OR e-psych* OR ccbt OR c-cbt OR "electronic cognitive behavioural therapy" OR "computerized cognitive behavioural therapy" OR "electronic psychology" OR "electronic psychotherapy" ) ) | 12,622,127 |
| #3 | TITLE ( ( "randomized controlled trial" OR "controlled clinical trial" OR randomized OR randomly OR trial OR placebo OR "clinical trial" ) ) | 631,662 |
| #4 | #1 AND #2 AND #3 | 846 |

| **Embase** | | |
| --- | --- | --- |
| Step | Search Strategy | Number of Records Retrieved |
| #1 | ('musculoskeletal pain'/exp OR 'musculoskeletal pain') AND [embase]/lim | 197048 |
| #2 | ('chronic pain'/exp OR 'chronic pain') AND [embase]/lim | 104965 |
| #3 | ('backache'/exp OR 'backache') AND [embase]/lim | 139458 |
| #4 | ('neck pain'/exp OR 'neck pain') AND [embase]/lim | 35631 |
| #5 | ('shoulder pain'/exp OR 'shoulder pain') AND [embase]/lim | 24341 |
| #6 | ('myofascial pain'/exp OR 'myofascial pain') AND [embase]/lim | 5613 |
| #7 | ('fibromyalgia'/exp OR 'fibromyalgia') AND [embase]/lim | 27760 |
| #8 | ('osteoarthritis'/exp OR 'osteoarthritis') AND [embase]/lim | 192847 |
| #9 | ('arthralgia'/exp OR 'arthralgia') AND [embase]/lim | 89029 |
| #10 | ('intractable pain'/exp OR 'intractable pain') AND [embase]/lim | 5969 |
| #11 | #1 OR #2 OR #3 OR #4 OR #5 OR #6 OR #7 OR #8 OR #9 OR #10 | 557622 |
| #12 | 'musculoskeletal pain':ab,ti OR 'chronic pain':ab,ti OR 'chronic musculoskeletal pain':ab,ti OR 'neck pain':ab,ti OR 'back pain':ab,ti OR 'low back pain':ab,ti OR 'shoulder pain':ab,ti OR 'knee pain':ab,ti OR fibromyalgia:ab,ti OR 'myofascial pain':ab,ti OR 'muscle pain':ab,ti OR 'joint pain':ab,ti OR arthralgia:ab,ti OR 'musculoskeletal disorder*':ab,ti OR 'lower limb pain':ab,ti OR 'upper limb pain':ab,ti OR 'persistent pain':ab,ti OR 'long-term pain':ab,ti | 310866 |
| #13 | #11 OR #12 | 649189 |
| #14 | ('mobile application'/exp OR 'mobile application') AND [embase]/lim | 26383 |
| #15 | ('virtual reality'/exp OR 'virtual reality') AND [embase]/lim | 33128 |
| #16 | ('virtual reality exposure therapy'/exp OR 'virtual reality exposure therapy') AND [embase]/lim | 857 |
| #17 | ('telerehabilitation'/exp OR 'telerehabilitation') AND [embase]/lim | 3588 |
| #18 | ('wearable computer'/exp OR 'wearable computer') AND [embase]/lim | 10522 |
| #19 | ('internet based cognitive behavioral therapy'/exp OR 'internet based cognitive behavioral therapy') AND [embase]/lim | 369 |
| #20 | ('randomized controlled trial'/exp OR 'randomized controlled trial') AND [embase]/lim | 1025136 |
| #21 | 'randomized controlled trial':ti OR 'controlled clinical trial':ti OR randomized:ti OR randomly:ti OR trial:ti OR 'clinical trial':ti | 730978 |
| #22 | #20 OR #21 | 1420165 |
| #23 | 'musculoskeletal pain':ti OR 'chronic pain':ti OR 'chronic musculoskeletal pain':ti OR 'neck pain':ti OR 'back pain':ti OR 'low back pain':ti OR 'shoulder pain':ti OR 'knee pain':ti OR fibromyalgia:ti OR 'myofascial pain':ti OR 'muscle pain':ti OR 'joint pain':ti OR arthralgia:ti OR 'musculoskeletal disorder*':ti OR 'lower limb pain':ti OR 'upper limb pain':ti OR 'persistent pain':ti OR 'long-term pain':ti | 105447 |
| #24 | #11 OR #23 | 587121 |
| #25 | 'mobile applications':ti OR mhealth:ti OR 'm health':ti OR 'mobile health':ti OR smartphone*:ti OR app:ti OR application*:ti OR 'mobile app':ti OR 'digital health':ti OR 'self-management app':ti OR 'mobile-based intervention':ti OR 'smartphone application':ti | 482705 |
| #26 | #14 OR #25 | 498405 |
| #27 | 'virtual reality':ti OR 'virtual reality exposure therapy':ti OR vr:ti OR 'virtual reality simulator':ti OR 'virtual reality system':ti OR 'immersive virtual reality':ti OR 'vr headset':ti OR 'virtual environment':ti OR 'head-mounted display':ti OR '3d simulation':ti OR 'interactive virtual system':ti | 18375 |
| #28 | #15 OR #16 OR #27 | 41073 |
| #29 | 'remote rehabilitation':ti OR telerehabilitation:ti OR 'virtual reality head-mounted display':ti | 2444 |
| #30 | #17 OR #29 | 4724 |
| #31 | wearable:ti OR 'wearable device':ti OR 'wearable sensor':ti OR 'body worn':ti OR tracker*:ti OR monitor*:ti OR wristband:ti OR smartwatch:ti OR pedometer*:ti OR accelerom*:ti OR actigraph*:ti OR actiwatch:ti OR actimeter:ti OR 'posture sensor*':ti OR 'posture monitor*':ti OR fitbit*:ti OR 'apple watch':ti OR withings:ti OR garmin:ti OR pebble:ti OR jawbone:ti OR sensewear:ti OR gt3x:ti OR 'body media':ti OR samsung:ti | 258837 |
| #32 | #18 OR #31 | 266783 |
| #33 | icbt:ti OR 'i-cbt':ti OR ccbt:ti OR 'c-cbt':ti OR 'electronic cognitive behavioural therapy':ti OR 'computerized cognitive behavioural therapy':ti OR 'electronic psychotherapy':ti OR epsych*:ti OR 'e-psych*':ti | 202 |
| #34 | #19 OR #33 | 554 |
| #35 | #26 OR #28 OR #30 OR #32 OR #34 | 796421 |
| #36 | #22 AND #24 AND #35 | 1379 |

| **Cochrane** | | |
| --- | --- | --- |
| Step | Search Strategy | Number of Records Retrieved |
| #1 | MeSH descriptor: [Musculoskeletal Pain] explode all trees | 1753 |
| #2 | MeSH descriptor: [Chronic Pain] explode all trees | 4868 |
| #3 | MeSH descriptor: [Back Pain] explode all trees | 7854 |
| #4 | MeSH descriptor: [Neck Pain] explode all trees | 2253 |
| #5 | MeSH descriptor: [Shoulder Pain] explode all trees | 1556 |
| #6 | MeSH descriptor: [Myofascial Pain Syndromes] explode all trees | 1777 |
| #7 | MeSH descriptor: [Fibromyalgia] explode all trees | 2075 |
| #8 | MeSH descriptor: [Osteoarthritis] explode all trees | 11284 |
| #9 | MeSH descriptor: [Pain, Intractable] explode all trees | 345 |
| #10 | MeSH descriptor: [Arthralgia] explode all trees | 2854 |
| #11 | #1 OR #2 OR #3 OR #4 OR #5 OR #6 OR #7 OR #8 OR #9 OR #10 | 30698 |
| #12 | (musculoskeletal pain OR chronic pain OR chronic musculoskeletal pain OR neck pain OR back pain OR low back pain OR shoulder pain OR knee pain OR fibromyalgia OR myofascial pain OR muscle pain OR joint pain OR arthralgia OR musculoskeletal disorder* OR lower limb pain OR upper limb pain OR persistent pain OR long-term pain):ti | 34622 |
| #13 | #11 OR #12 | 51744 |
| #14 | MeSH descriptor: [Mobile Applications] explode all trees | 2427 |
| #15 | (Mobile Applications OR mhealth OR m-health OR mobile health OR smartphone* OR app OR application* OR mobile app OR digital health OR self-management app OR mobile-based intervention OR smartphone application):ti | 30271 |
| #16 | #14 OR #15 | 30828 |
| #17 | MeSH descriptor: [Virtual Reality] explode all trees | 1448 |
| #18 | MeSH descriptor: [Virtual Reality Exposure Therapy] explode all trees | 385 |
| #19 | (virtual reality OR Virtual Reality Exposure Therapy OR VR OR virtual reality simulator OR virtual reality system OR immersive virtual reality OR VR headset OR virtual environment OR head-mounted display OR 3D simulation OR interactive virtual system):ti | 6566 |
| #20 | MeSH descriptor: [Telerehabilitation] explode all trees | 418 |
| #21 | (remote rehabilitation OR telerehabilitation OR "virtual reality head-mounted display"):ti,ab,kw | 2967 |
| #22 | #20 OR #21 | 2967 |
| #23 | #17 OR #18 OR #19 | 6862 |
| #24 | MeSH descriptor: [Wearable Electronic Devices] explode all trees | 1111 |
| #25 | (iCBT OR "i-CBT" OR cCBT OR "c-CBT" OR "electronic cognitive behavioural therapy" OR "computerized cognitive behavioural therapy" OR "electronic psychotherapy" OR ePsych* OR "e-Psych*"):ti | 231 |
| #26 | MeSH descriptor: [Randomized Controlled Trial] explode all trees | 34 |
| #27 | (randomized controlled trial OR controlled clinical trial OR randomized OR randomly OR trial OR clinical trial):ti | 635419 |
| #28 | #26 OR #27 | 635431 |
| #29 | (wearable OR "wearable device" OR "wearable sensor" OR body-worn OR tracker* OR monitor* OR wristband OR smartwatch OR pedometer* OR accelerom* OR actigraph* OR actiwatch OR actimeter OR posture sensor* OR posture monitor* OR fitbit* OR "Apple Watch" OR Withings OR Garmin OR Pebble OR Jawbone OR SenseWear OR GT3X OR "body media" OR Samsung):kw | 50515 |
| #30 | #24 OR #29 | 50975 |
| #31 | #16 OR #22 OR #23 OR #25 OR #30 | 89694 |
| #32 | #28 AND #31 AND #13 | 710 |

**eTable 2. Results of the updated systematic literature search conducted prior to submission (January 29, 2026)**

To ensure that the most recent and relevant evidence was captured, an updated literature search was conducted prior to manuscript submission. The updated search applied the same search strategies, databases, and eligibility criteria as the initial search, with the publication date restricted to July 10, 2025 to January 29, 2026. Details of the updated search strategies and the number of records retrieved from each database are provided in eTable 3

| **Database** | **Number of Records Retrieved** |
| --- | --- |
| PubMed | 106 |
| CINAHL | 115 |
| WOS | 96 |
| Embase & MEDLINE | 11 |
| Scopus | 375 |
| Cochrane | 16 |
| Total | 701 |

| **PubMed（07/10/2025 - 02/02/2026）** | | |
| --- | --- | --- |
| Step | Search Strategy | Number of Records Retrieved |
| #1 | "Musculoskeletal Pain"[Mesh] OR "Chronic Pain"[Mesh] OR "Back Pain"[Mesh] OR "Neck Pain"[Mesh] OR "Shoulder Pain"[Mesh] OR "Myofascial Pain Syndromes"[Mesh] OR "Fibromyalgia"[Mesh] OR "Osteoarthritis"[Mesh] OR "Arthralgia"[Mesh] OR "Pain, Intractable"[Mesh] OR musculoskeletal pain[Title] OR chronic pain[Title] OR chronic musculoskeletal pain[Title] OR neck pain[Title] OR back pain[Title] OR low back pain[Title] OR shoulder pain[Title] OR knee pain[Title] OR fibromyalgia[Title] OR myofascial pain[Title] OR muscle pain[Title] OR joint pain[Title] OR arthralgia[Title] OR musculoskeletal disorder*[Title] OR lower limb pain[Title] OR upper limb pain[Title] OR persistent pain[Title] OR long-term pain[Title] | 234, 402 |
| #2 | "Mobile Applications"[Mesh] OR "Virtual Reality"[Mesh] OR "Virtual Reality Exposure Therapy"[Mesh] OR "Telerehabilitation"[Mesh] OR "Wearable Electronic Devices"[Mesh] OR "Cognitive Behavioral Therapy"[Mesh] OR mobile applications[Title] OR mhealth[Title] OR mobile health[Title] OR smartphone*[Title] OR app[Title] OR digital health[Title] OR virtual reality[Title] OR VR[Title] OR telerehabilitation[Title] OR wearable[Title] OR wearable device[Title] OR smartwatch[Title] OR tracker*[Title] OR iCBT[Title] OR cCBT[Title] | 137,793 |
| #3 | randomized controlled trial[Title/Abstract] OR controlled clinical trial[Title/Abstract] OR randomized[Title/Abstract] OR randomly[Title/Abstract] OR trial[Title/Abstract] | 1,686,779 |
| #4 | "2025/07/11"[Date - Publication] : "2026/01/29"[Date - Publication] | 1,033,388 |
| #5 | #1 AND #2 AND #3 AND #4 | 106 |

| **Cochrane（07/10/2025 - 29/01/2026）** | | |
| --- | --- | --- |
| Step | Search Strategy | Number of Records Retrieved |
| #1 | MeSH descriptor: [Musculoskeletal Pain] explode all trees | 1833 |
| #2 | MeSH descriptor: [Chronic Pain] explode all trees | 5204 |
| #3 | MeSH descriptor: [Back Pain] explode all trees | 8082 |
| #4 | MeSH descriptor: [Neck Pain] explode all trees | 2353 |
| #5 | MeSH descriptor: [Shoulder Pain] explode all trees | 1615 |
| #6 | MeSH descriptor: [Myofascial Pain Syndromes] explode all trees | 1800 |
| #7 | MeSH descriptor: [Fibromyalgia] explode all trees | 2153 |
| #8 | MeSH descriptor: [Osteoarthritis] explode all trees | 17 |
| #9 | MeSH descriptor: [Pain, Intractable] explode all trees | 346 |
| #10 | MeSH descriptor: [Arthralgia] explode all trees | 2933 |
| #11 | #1 OR #2 OR #3 OR #4 OR #5 OR #6 OR #7 OR #8 OR #9 OR #10 | 20985 |
| #12 | (musculoskeletal pain OR chronic pain OR chronic musculoskeletal pain OR neck pain OR back pain OR low back pain OR shoulder pain OR knee pain OR fibromyalgia OR myofascial pain OR muscle pain OR joint pain OR arthralgia OR musculoskeletal disorder* OR lower limb pain OR upper limb pain OR persistent pain OR long-term pain) | 34622 |
| #13 | #11 OR #12 | 51744 |
| #14 | MeSH descriptor: [Mobile Applications] explode all trees | 2427 |
| #15 | (Mobile Applications OR mhealth OR m-health OR mobile health OR smartphone* OR app OR application* OR mobile app OR digital health OR self-management app OR mobile-based intervention OR smartphone application) | 30271 |
| #16 | #14 OR #15 | 33433 |
| #17 | MeSH descriptor: [Virtual Reality] explode all trees | 1448 |
| #18 | MeSH descriptor: [Virtual Reality Exposure Therapy] explode all trees | 385 |
| #19 | (virtual reality OR Virtual Reality Exposure Therapy OR VR OR virtual reality simulator OR virtual reality system OR immersive virtual reality OR VR headset OR virtual environment OR head-mounted display OR 3D simulation OR interactive virtual system) | 6566 |
| #20 | MeSH descriptor: [Telerehabilitation] explode all trees | 418 |
| #21 | (remote rehabilitation OR telerehabilitation OR "virtual reality head-mounted display") | 2967 |
| #22 | #20 OR #21 | 2967 |
| #23 | #17 OR #18 OR #19 | 7821 |
| #24 | MeSH descriptor: [Wearable Electronic Devices] explode all trees | 1111 |
| #25 | (iCBT OR i-CBT OR cCBT OR c-CBT OR electronic cognitive behavioural therapy OR computerized cognitive behavioural therapy OR electronic psychotherapy OR ePsych* OR e-Psych*) | 258 |
| #26 | MeSH descriptor: [Randomized Controlled Trial] explode all trees | 34 |
| #27 | (randomized controlled trial OR controlled clinical trial OR randomized OR randomly OR trial OR clinical trial) | 635419 |
| #28 | #26 OR #27 | 635431 |
| #29 | (wearable OR wearable device OR wearable sensor OR body-worn OR tracker* OR monitor* OR wristband OR smartwatch OR pedometer* OR accelerom* OR actigraph* OR actiwatch OR actimeter OR posture sensor* OR posture monitor* OR fitbit* OR Apple Watch OR Withings OR Garmin OR Pebble OR Jawbone OR SenseWear OR GT3X OR body media OR Samsung) | 734445 |
| #30 | #24 OR #29 | 735018 |
| #31 | #16 OR #22 OR #23 OR #25 OR #30 | 764566 |
| #32 | #28 AND #31 AND #13 | 16 |

| **CINAHL（07/10/2025 - 02/02/2026）** | | |
| --- | --- | --- |
| Step | Search Strategy | Number of Records Retrieved |
| S1 | XB musculoskeletal pain OR chronic pain OR chronic musculoskeletal pain OR neck pain OR back pain OR low back pain OR shoulder pain OR knee pain OR fibromyalgia OR myofascial pain OR muscle pain OR joint pain OR arthralgia OR musculoskeletal disorder* OR lower limb pain OR upper limb pain OR persistent pain OR long-term pain | 4428 |
| S2 | [(MH musculoskeletal pain OR MH chronic pain OR MH back pain OR MH neck pain OR MH shoulder pain) OR (MH osteoarthritis OR MH arthralgia OR MH pain intractable OR MH fibromyalgia OR MH myofascial pain syndrome)](https://libdb.csu.edu.cn/search/results?db=c8h&expanders=concept&limiters=DT1:2025-07-01/2026-02-02&searchMode=boolean&sort=relevance&sqId=sq:8a04bd6e-3a19-4023-9424-9f480cbaf5b1&userDirectAction=ue) | 1864 |
| S3 | S2 OR S1 | 4466 |
| S4 | (MH mobile applications OR XB (Mobile Applications OR mhealth OR m-health OR mobile health OR smartphone* OR app OR application* OR mobile app OR digital health OR self-management app OR mobile-based intervention OR smartphone application)) OR (MH virtual reality OR XB (virtual reality OR Virtual Reality Exposure Therapy OR VR OR virtual reality simulator OR virtual reality system OR immersive virtual reality OR VR headset OR virtual environment OR head-mounted display OR 3D simulation OR interactive virtual system) OR MH virtual reality exposure therapy) OR (MH telerehabilitation OR XB (remote rehabilitation OR telerehabilitation OR virtual reality head-mounted display)) OR (MH wearable electronic devices OR XB (wearable OR "wearable device" OR "wearable sensor" OR body-worn OR tracker* OR monitor* OR wristband OR smartwatch OR pedometer* OR accelerom* OR actigraph* OR actiwatch OR actimeter OR posture sensor* OR posture monitor* OR fitbit* OR "Apple Watch" OR Withings OR Garmin OR Pebble OR Jawbone OR SenseWear OR GT3X OR "body media" OR Samsung)) OR (MH internet-based cognitive behavioural therapy OR XB (iCBT OR "i-CBT" OR cCBT OR "c-CBT" OR "electronic cognitive behavioural therapy" OR "computerized cognitive behavioural therapy" OR "electronic psychotherapy" OR ePsych* OR "e-Psych*")) | 15905 |
| S5 | MH Randomized Controlled Trial OR XB (randomized controlled trial OR controlled clinical trial OR randomized OR randomly OR trial OR clinical trial) | 18114 |
| S6 | S5 AND S4 AND S3 | 115 |

| **Web of Science（07/10/2025 - 02/02/2026）** | | |
| --- | --- | --- |
| Step | Search Strategy | Number of Records Retrieved |
| #1 | TI=(musculoskeletal pain OR chronic pain OR chronic musculoskeletal pain OR neck pain OR back pain OR low back pain OR shoulder pain OR knee pain OR fibromyalgia OR myofascial pain OR muscle pain OR joint pain OR arthralgia OR musculoskeletal disorder* OR lower limb pain OR upper limb pain OR persistent pain OR long-term pain) and Preprint Citation Index (Exclude – Database) | 186877 |
| #2 | ((((TS=(Mobile Applications OR mhealth OR m-health OR mobile health OR smartphone* OR app OR application* OR mobile app OR digital health OR self-management app OR mobile-based intervention OR smartphone application)) OR TS=(virtual reality OR Virtual Reality Exposure Therapy OR VR OR virtual reality simulator OR virtual reality system OR immersive virtual reality OR VR headset OR virtual environment OR head-mounted display OR 3D simulation OR interactive virtual system)) OR TS=(remote rehabilitation OR telerehabilitation OR "virtual reality head-mounted display")) OR TS=(wearable OR "wearable device" OR "wearable sensor" OR body-worn OR tracker* OR monitor* OR wristband OR smartwatch OR pedometer* OR accelerom* OR actigraph* OR actiwatch OR actimeter OR posture sensor* OR posture monitor* OR fitbit* OR "Apple Watch" OR Withings OR Garmin OR Pebble OR Jawbone OR SenseWear OR GT3X OR "body media" OR Samsung)) OR TS=(“iCBT” OR "i-CBT" OR “cCBT” OR "computerized cognitive behavioural therapy" OR ePsych* OR "e-Psych*") and Preprint Citation Index (Exclude – Database) | 19984590 |
| #3 | TI=(randomized controlled trial OR controlled clinical trial OR randomized OR randomly OR trial OR clinical trial) and Preprint Citation Index (Exclude – Database) | 836148 |
| #4 | #1 AND #2 AND #3 and Preprint Citation Index (Exclude – Database) | 1518 |
| #5 | 2025-07-11/2026-01-29 (Publication Date) and Preprint Citation Index (Exclude – Database) | 4418993 |
| #6 | #9 AND #8 and Preprint Citation Index (Exclude – Database) | 96 |

| **SCOPUS（07/10/2025 - 02/02/2026）** | | |
| --- | --- | --- |
| Step | Search Strategy | Number of Records Retrieved |
| #1 | TITLE-ABS-KEY ( ( "musculoskeletal pain" OR "chronic pain" OR "chronic musculoskeletal pain" OR "neck pain" OR "back pain" OR "low back pain" OR "shoulder pain" OR "knee pain" OR fibromyalgia OR "myofascial pain" OR "muscle pain" OR "joint pain" OR arthralgia OR "musculoskeletal disorder*" OR "lower limb pain" OR "upper limb pain" OR "persistent pain" OR "long-term pain" OR "intractable pain" ) ) | 451,030 |
| #2 | TITLE-ABS-KEY ( ( "mobile applications" OR mhealth OR "mobile health" OR smartphone* OR app OR application* OR "mobile app" OR "digital health" OR "self-management app" OR "mobile-based intervention" OR "smartphone application" OR "virtual reality" OR "virtual reality exposure therapy" OR vr OR "virtual reality simulator" OR "virtual reality system" OR "immersive virtual reality" OR "VR headset" OR "virtual environment" OR "head-mounted display" OR "3D simulation" OR "interactive virtual system" OR "remote rehabilitation" OR telerehabilitation OR "virtual reality head-mounted display" OR wearable OR "wearable device" OR "wearable sensor" OR "body-worn" OR tracker* OR monitor* OR wristband OR smartwatch OR pedometer* OR accelerom* OR actigraph* OR actiwatch OR actimeter OR "posture sensor*" OR "posture monitor*" OR fitbit* OR "apple watch" OR withings OR garmin OR pebble OR jawbone OR sensewear OR gt3x OR "body media" OR samsung OR icbt OR i-cbt OR epsych* OR e-psych* OR ccbt OR c-cbt OR "electronic cognitive behavioural therapy" OR "computerized cognitive behavioural therapy" OR "electronic psychology" OR "electronic psychotherapy" ) ) | 13,335,938 |
| #3 | TITLE ( ( "randomized controlled trial" OR "controlled clinical trial" OR randomized OR randomly OR trial OR placebo OR "clinical trial" ) ) | 662,615 |
| #4 | (#1 AND #2 AND #3) AND ( LIMIT-TO ( PUBYEAR , 2025 ) OR LIMIT-TO ( PUBYEAR , 2026 ) ) | 375 |

| **Embase & MEDLINE（07/10/2025 - 30/01/2026）** | | |
| --- | --- | --- |
| Step | Search Strategy | Number of Records Retrieved |
| #1 | (((((((((((((((('musculoskeletal pain'/exp OR 'musculoskeletal pain' OR 'chronic pain'/exp OR 'chronic pain' OR 'backache'/exp OR 'backache' OR 'neck pain'/exp OR 'neck pain' OR 'shoulder pain'/exp OR 'shoulder pain' OR 'myofascial pain'/exp OR 'myofascial pain' OR 'fibromyalgia'/exp OR 'fibromyalgia' OR 'osteoarthritis'/exp OR 'osteoarthritis' OR 'arthralgia'/exp OR 'arthralgia' OR 'intractable pain'/exp OR 'intractable pain' OR musculoskeletal) AND pain:ti,ab OR chronic) AND pain:ti,ab OR chronic) AND musculoskeletal AND pain:ti,ab OR 'neck'/exp OR neck) AND pain:ti,ab OR 'back'/exp OR back) AND pain:ti,ab OR low) AND ('back'/exp OR back) AND pain:ti,ab OR 'shoulder'/exp OR shoulder) AND pain:ti,ab OR 'knee'/exp OR knee) AND pain:ti,ab OR fibromyalgia:ti,ab OR myofascial) AND pain:ti,ab OR 'muscle'/exp OR muscle) AND pain:ti,ab OR 'joint'/exp OR joint) AND pain:ti,ab OR arthralgia:ti,ab OR musculoskeletal) AND disorder*:ti,ab OR lower) AND ('limb'/exp OR limb) AND pain:ti,ab OR upper) AND ('limb'/exp OR limb) AND pain:ti,ab OR persistent) AND pain:ti,ab OR 'long term') AND pain:ti,ab AND [embase]/lim AND ([embase]/lim OR [medline]/lim) AND [11-07-2025]/sd NOT [30-01-2026]/sd AND [2025-2026]/py | 9498 |
| #2 | ((('mobile application'/exp OR 'mobile application' OR 'virtual reality'/exp OR 'virtual reality' OR 'virtual reality exposure therapy'/exp OR 'virtual reality exposure therapy' OR 'telerehabilitation'/exp OR 'telerehabilitation' OR 'wearable computer'/exp OR 'wearable computer' OR 'internet based cognitive behavioral therapy'/exp OR 'internet based cognitive behavioral therapy' OR mobile) AND application*:ti OR mhealth:ti OR 'mobile health':ti OR smartphone*:ti OR app:ti OR application*:ti OR 'mobile app':ti OR 'digital health':ti OR 'self-management app':ti OR 'mobile-based intervention':ti OR 'smartphone application':ti OR virtual) AND reality:ti OR vr:ti OR telerehabilitation:ti OR 'remote rehabilitation':ti OR wearable:ti OR 'wearable device':ti OR smartwatch:ti OR tracker*:ti OR icbt:ti OR ccbt:ti OR epsych*:ti) AND [embase]/lim AND ([embase]/lim OR [medline]/lim) AND [2025-2026]/py | 3605 |
| #3 | ((('randomized controlled trial'/exp OR 'randomized controlled trial' OR randomized) AND controlled AND trial:ti OR controlled) AND ('clinical'/exp OR clinical) AND trial:ti OR randomized:ti OR randomly:ti OR trial:ti OR 'clinical'/exp OR clinical) AND trial:ti AND [embase]/lim AND ([embase]/lim OR [medline]/lim) AND [2025-2026]/py | 34656 |
| #4 | #1 AND #2 AND #3 | 11 |
