# Supplementary material for: Effects of Digital Health Interventions in Chronic Musculoskeletal Pain: Systematic Review and Bayesian Network Meta-Analysis of Randomized Controlled Trials
Source: J Med Internet Res. 2026 Oct 7;28:e95903. doi: 10.2196/95903 (PMC13645338; doi:10.2196/95903)
Supplement: Supplementary file 3 [file jmir-v28-e95903-s002.docx]

# Multimedia Appendix 2. Classification, coding scheme, and operational definitions of digital health interventions and control conditions used for subgroup analyses and Bayesian network meta-analysis.

# eTable 1. Hierarchical classification and operational definitions of interventions and control conditions

| **Domain (delivery modality)** | **Code / category** | **Intervention or control category** | **Operational definition** |
| --- | --- | --- | --- |
| Mobile application (APP)–based interventions: DHIs delivered primarily via smartphone or tablet applications used as the principal platform for pain management content. | APP-Exercise | App-based exercise-only | Mobile application–based interventions in which structured physical exercise or functional training constituted the primary therapeutic component. |
|  | APP-Ex+Edu | App-based exercise plus education | Exercise combined with pain education or self-management guidance without psychological therapy. |
|  | APP-CBT | App-based CBT | Mobile-based cognitive behavioral therapy targeting pain-related cognition and coping. |
|  | APP-Ex+CBT+Edu | App-based multimodal | Exercise + CBT + education with no dominant component. |
|  | APP-MRF | Monitoring and feedback | Symptom tracking, reminders, or feedback-based APP interventions. |
|  | APP-Ex+CBT | Exercise plus CBT | Combined exercise and CBT without education module. |
|  | APP-Education | Education-only | Pain education or self-management information as primary content. |
| Virtual reality (VR)–based interventions: DHIs delivered through immersive or non-immersive virtual environments. | MF-VR | Motor/functional VR | VR-based motor or functional training interventions. |
|  | DA-VR | Distraction/analgesic VR | VR targeting pain relief via distraction or immersion. |
|  | CP-VR | Cognitive/psychological VR | VR targeting cognitive or emotional pain processes. |
| Wearable device (WD)–based interventions: DHIs using body-worn sensors for monitoring and feedback. | WD | Wearable device | Monitoring physical activity, posture, or movement with feedback. |
| Telerehabilitation interventions: remote rehabilitation via telecommunication technologies. | T-E | Exercise-only | Remote supervised exercise training. |
|  | T-EE | Exercise + education | Exercise plus self-management education. |
|  | T-CBT | Psychological therapy | Remote CBT or psychological interventions. |
| Multimodal digital interventions (MD): integration of ≥2 digital modalities. | MD | Multimodal digital | Integration of app, VR, wearable, or telehealth technologies. |
| Active control: non-digital clinical management. | AC | Active control | Usual care, rehab, education without digital components. |
| Non-active control: minimal or no structured intervention. | MI/WL | Minimal intervention / wait-list | Passive education, general advice, or no treatment. |
