# Supplementary material for: Effects of Digital Health Interventions in Chronic Musculoskeletal Pain: Systematic Review and Bayesian Network Meta-Analysis of Randomized Controlled Trials
Source: J Med Internet Res. 2026 Oct 7;28:e95903. doi: 10.2196/95903 (PMC13645338; doi:10.2196/95903)
Supplement: Supplementary file 4 [file jmir-v28-e95903-s003.docx]

# Multimedia Appendix 3. Definitions, measurement hierarchy, and effect measures for pain intensity, functional disability, and health-related quality-of-life outcomes.

# eTable 1. Definition of outcomes

| **Outcome** | **Definition** | **Effect measure** |
| --- | --- | --- |
| Pain intensity | Change in pain intensity from baseline to the closest post-intervention time point. When multiple follow-up time points were reported, the assessment closest to the end of the intervention was used. For crossover trials, only first-period data were included. When multiple measures were available, preference was given to validated self-report scales (VAS, NPRS, BPI, MPQ, WOMAC pain subscale, KOOS/HOOS pain subscale, Von Korff Pain Intensity scale). | Standardized mean difference (SMD) |
| Functional status / disability-related outcomes | Change in functional status or disability from baseline to follow-up. These outcomes reflected limitations in daily, occupational, or physical functioning due to musculoskeletal pain. When multiple instruments were reported, one was selected according to a predefined hierarchy prioritizing disease-specific disability measures, followed by condition-specific and generic functional measures. Instruments included RMDQ, ODI, NDI, HAQ, WOMAC physical function, KOOS/HOOS ADL, SPADI disability, FIQ/FIQR, WHODAS, PROMIS Physical Function, MSK-HQ, and Barthel Index. | Standardized mean difference (SMD) |
| Health-related quality of life (HRQoL) | Change in health-related quality of life from baseline to follow-up. When both generic and condition-specific instruments were reported, generic measures were prioritized for cross-study comparability. Measures included EQ-5D, EQ VAS, SF-36, SF-12, AQoL-6D, and WHOQOL. | Narrative synthesis |
