# Supplementary material for: Effects of Digital Health Interventions in Chronic Musculoskeletal Pain: Systematic Review and Bayesian Network Meta-Analysis of Randomized Controlled Trials
Source: J Med Internet Res. 2026 Oct 7;28:e95903. doi: 10.2196/95903 (PMC13645338; doi:10.2196/95903)
Supplement: Supplementary file 5 [file jmir-v28-e95903-s004.docx]

**Multimedia Appendix 4. Characteristics of the included randomized controlled trials.**

| **ID** | **Study** | **year** | **Country** | **Disease type** | **Pain duration** | **Intervention category** | **Sample size(M/F)** | **Age (mean+SD)** | **Intervention frequency** | **Duration** | **Pain Outcome measures** | **Functional disability outcome measures** | **Quality of life outcome measures** | **Follow-up time** |
| --- | --- | --- | --- | --- | --- | --- | --- | --- | --- | --- | --- | --- | --- | --- |
| 1. | Zhu et al.[1] | 2025 | China | Knee osteoarthritis (KOA) | ≥3 months | CV-based graded exercise APP | 32（M=13, F=19) | 68.59±7.16 | 3 times per week | Six weeks | WOMAC | WOMAC physical function | AQoL-6D | Baseline；6w |
|  |  |  |  |  |  | Active control | 28（M=13, F=15) | 67.64±7.85 |  |  |  |  |  |  |
| 2. | Östlind et al.[2] | 2025 | Sweden | Hip and knee osteoarthritis | NA | SOASP + wearable activity tracker | 74（M=9, F=65) | 56.7±5.3 | from morning until bedtime | 12 weeks | HOOS/KOOS | HOOS/KOOS ADL | EQ-5D | Baseline；3m；6m;12m |
|  |  |  |  |  |  | Active control | 50（M=12, F=38) | 54.5±6.2 |  |  |  |  |  |  |
| 3. | Lo, H. H. M. et al.[3] | 2025 | China (Hong Kong) | Chronic musculoskeletal pain in older adults | ≥3 months | VR-CAVE with Tai Chi | 40（M=7, F=33) | 65.7±5.5 | 1 times per week | 8 weeks | BPI | Not reported | EQ-5D | Baseline; 8 w(post-intervention)；12 w |
|  |  |  |  |  |  | Active control | 40（M=10, F=30) | 64.6±5.7 |  |  |  |  |  |  |
| 4. | Lindner et al.[4] | 2025 | Germany | Chronic back pain | ≥3 months | Digitally supported physical activity counselling (ExPa app) | 14（M=9, F=5) | Median age 59 (36–83) | 15-minute ExPa-guided consultation (1 time) + optional follow-up | 3 months | Von Korff pain | Not reported | SF-12 PCS; SF-12 MCS | Baseline; 3m |
|  |  |  |  |  |  | Non-active control | 17（M=4, F=13) | 57 (21–81) |  |  |  |  |  |  |
| 5. | Darcy et al.[5] | 2025 | USA | Knee osteoarthritis | NA | Medial off-loader brace + sensor-based platform | 30（M=9, F=21) | 63.9±7.15 | Brace wearing: Week1 (2h/d), Week2 (4h/d), Week3+(≥6h/d); Exercise: Daily (3 sets) | 12 weeks | KOOS Pain; KOOS-ADL, QoL | KOOS ADL | KOOS QoL | Baseline; 12w |
|  |  |  |  |  |  | Active control | 30（M=7, F=23) | 65.03±7.61 |  |  |  |  |  |  |
| 6. | Özden et al.[6] | 2024 | Turkey | Chronic low back pain | ≥3 months | PhysioAnalyst app + visual feedback | 22（M=7, F=15) | 43.5 ± 12.58 | Daily | 8 weeks | VAS; ODI | Oswestry Disability Index (ODI) | NHP First Section Total | Baseline; 4w; 8w |
|  |  |  |  |  |  | Active control | 22（M=12, F=10) | 46.22 ± 12.38 |  |  |  |  |  |  |
| 7. | Nishitha et al.[7] | 2024 | India | Post-TKA chronic pain | NA | total | 36(F=21, M=15) | 51.2±5.2 | 4 sessions/week | 12 weeks | NPRS | WOMAC total score | Not reported | 2W; 3W; 4W; 9W |
|  |  |  |  |  |  | VR-based rehabilitation(immersive + non-immersive gamified training) | 18 | NA |  |  |  |  |  |  |
|  |  |  |  |  |  | Active control | 18 | NA |  |  |  |  |  |  |
| 8. | Kim & Shin[8] | 2024 | South Korea | Non-specific chronic neck pain | ≥6 months | Video-based smartphone app | 17(F=12, M=5) | 41.53±12.67 | 3 times/week | 6 weeks | NPRS | Neck Disability Index (NDI) | Not reported | Baseline;6w |
|  |  |  |  |  |  | Active control | 17(F=11, M=6) | 37.15±10.92 |  |  |  |  |  |  |
| 9. | Dieter et al.[9] | 2024 | Germany | Moderate to severe unicondylar knee osteoarthritis | NA | Sensor-assisted app-based exercise (re.flex) | 30(each15)(F=12, M=18) | 61.5±7.5 | 3 times a week | 12 weeks, | KOOS | KOOS physical function (ADL) | KOOS QoL | Baseline；3m |
|  |  |  |  |  |  | Non-active control | 31(F=18, M=13) | 64.2±9.3 |  |  |  |  |  |  |
| 10. | Weber et al.[10] | 2023 | Germany | Hip/knee osteoarthritis | ≥3 months | Join2Move app (graded physical activity | 32(F=21, M=11) | 60 ± 6 | 3 times/week; | 12 weeks | HOOS/KOOS; | HOOS/KOOS ADL | KOOS QoL | Baseline; 12w |
|  |  |  |  |  |  | Non-active control | 28(F=16, M=12) | 64 ± 8 |  |  |  |  |  |  |
| 11. | Tore et al.[11] | 2023 | Turkey | Knee osteoarthritis (KOA) | ≥3 months | Telerehabilitation(real-time video guidance + exercise) | 24(F=21, M=3) | 55.87 ± 7.24 | 3 sessions/week | 8 weeks | NRS (pain) | KOOS ADL | KOOS QoL | Baseline; 12w |
|  |  |  |  |  |  | Active control | 24(F=12, M=2) | 55.79 ± 6.76 |  |  |  |  |  |  |
| 12. | Thiengwittayaporn et al.[12] | 2023 | Thailand | Knee osteoarthritis | ≥3 months | "Rak Kao" mobile app (disease assessment + personalized exercise guidance) | 42(F=36, M=6) | 62.2 ± 6.8 | Daily | 4 weeks | KOOS | KOOS ADL | KOOS QoL | Baseline; 4w |
|  |  |  |  |  |  | Non-active control | 40  (F=37, M=3) | 63.0 ± 9.7 |  |  |  |  |  |  |
| 13. | Rodríguez Sánchez-Laulhé et al.[13] | 2023 | Spain | Symptomatic hand osteoarthritis (HOA) | ≥6 months | CareHand mobile app (exercise + education + self-management) | 34 (F=25, M=9), 66 symptomatic hands | 62.2 ± 8.8 | 4 times/week | 12 weeks | NRS | AUSCAN physical function | Not reported | Baseline; 1m; 3m; 6m |
|  |  |  |  |  |  | Non-active control | 40 (F=25, M=15),78 symptomatic hands | 64.3 ± 7.7 |  |  |  |  |  |  |
| 14. | Özlü et al.[14] | 2023 | Turkey | Knee osteoarthritis (stage 2–3) | NA | VR-mediated gamified rehabilitation + conventional treatment | 35 (F=17, M=18) | 53.28±10.42 | 5 times/week | 3 weeks | VAS | WOMAC total score | Not reported | Baseline; 3w; 7w |
|  |  |  |  |  |  | Non-active control | 38 (F=26, M=12) | 53.71±9.65 |  |  |  |  |  |  |
| 15. | Lee et al.[15] | 2023 | South Korea | Knee osteoarthritis (KL grade 1–2) | NA | Wearable device–assisted remote rehabilitation | 15 (F=15, M=0) | 65.63±3.70 | 3 times/week | 8 weeks | VAS | Five Times Sit-to-Stand Test (FTsST) | Not reported | Baseline; 8w |
|  |  |  |  |  |  | Non-active control | 16 (F=16, M=0) | 68.27±4.78 |  |  |  |  |  |  |
| 16. | Groenveld et al.[16] | 2023 | Netherlands | Chronic low back pain | ≥3 months | Behavioral therapy-based Virtual Reality | 20(F=17, M=3) | 51±2.9 | daily | 4 weeks | VAS | Not reported | SF-12 PCS; SF-12 MCS | Baseline; 4w; 4m |
|  |  |  |  |  |  | Non-active control | 20(F=16, M=4) | 52±2.5 |  |  |  |  |  |  |
| 17. | Hernando-Garijo et al.[17] | 2021 | Spain | Fibromyalgia | ≥3 months | Video-guided telerehabilitation aerobic exercise | 17(F=25, M=15) | 51.81±9.05 | 2 sessions/week | 15 weeks | VAS | Revised Fibromyalgia Impact Questionnaire (FIQ-R) | FIQ-R total score | Baesline; 15w |
|  |  |  |  |  |  | Non-active control | 17(F=25, M=15) | 55.06±8.51 |  |  |  |  |  |  |
| 18. | Gohir et al.[18] | 2021 | United Kingdom | Knee osteoarthritis | ≥3 month | Internet-based exercise program (Joint Academy app) | 48(F=34, M=14) | 65.2±9.7 | daily | 6 weeks | NRS | WOMAC physical function | Not reported | Baseline; 6w |
|  |  |  |  |  |  | Non-active control | 57(F=37, M=20) | 68.0±8.6 |  |  |  |  |  |  |
| 19. | Li et al.[19] | 2020 | Canada | Knee osteoarthritis | NA | Wearable multifaceted program (Fitbit Flex-2 + FitViz + PT counseling) | 26(F=23, M=3) | 65.0±8.3 | Daily | 12 weeks | KOOS | KOOS ADL | KOOS QoL | Baseline, 13 weeks, 26 weeks, 39 weeks |
|  |  |  |  |  |  | Delayed intervention | 25(F=19, M=6) | 64.8±9.0 |  |  |  |  |  |  |
| 20. | Petrozzi et al.[20] | 2019 | Australia | Chronic non-specific low back pain | >3 months | MoodGYM （APP）internet-delivered CBT | 54(F=27, M=27) | 50.1±12.8 | one module per week | 8 weeks | PNRS | Roland-Morris Disability Questionnaire (RMDQ) | Not reported | Baseline, 8 weeks, 6 months, 12 months |
|  |  |  |  |  |  | Active control | 54(F=27, M=27) | 50.6±14.4 |  |  |  |  |  |  |
| 21. | Rezaei et al.[21] | 2019 | Iran | Non-specific chronic neck pain | >3 months; mean ~22 months | VR training (Cervigame®) | 21(F=9, M=12) | 36.19±9.80 | 2 times/week | 4 weeks | VAS | Neck Disability Index (NDI) | Not reported | Baseline, post-intervention, 5 weeks |
|  |  |  |  |  |  | Active control | 21(F=11, M=10) | 31.23±9.49 |  |  |  |  |  |  |
| 22. | Irvine et al.[22] | 2015 | USA | Nonspecific low back pain | ≥3 months | FitBack mobile-Web self-management app | 199(F=116, M=83) | NA | FitBack group: Weekly email reminder; Alternative care group: 8 emails (within 8 weeks) | 8 weeks | NRS | Functional interference score | Not reported | Baseline, 2 months, 4 months |
|  |  |  |  |  |  | Active control | 199(F=125, M=74) | NA |  |  |  |  |  |  |
| 23. | Bahat et al.[23] | 2015 | Australia | Chronic neck pain | ≥3 months | KT + Virtual Reality (KTVR) | 16（11F/5M） | 40.63 ± 14.18 | 4–6 times within 5 weeks (no fixed frequency per week) | 5 weeks | VAS | Neck Disability Index (NDI) | Not reported | Baseline, 5 weeks), 3-month |
|  |  |  |  |  |  | Active control | 16（11F/5M） | 41.13 ± 12.59 |  |  |  |  |  |  |
| 24. | Rini et al.[24] | 2015 | USA | Knee/hip Osteoarthritis (OA) | ≥3 months | Internet-based automated pain coping skills training program (PainCOACH) | 58 （12M/46F) | 67.6 ± 9.5 | One module per week (a total of 8 modules), to be completed independently. There is no fixed frequency for training; daily practice of these skills is encouraged. | 8w | AIMS2 Pain subscale | Pain-related functioning | Not reported | Baseline , 5W; (9–11 weeks |
|  |  |  |  |  |  | Active control | 55 （10M/45F) | 66.67±11.02 |  |  |  |  |  |  |
| 25. | Thomas et al.[25] | 2016 | USA | Chronic low back pain (CLBP) with high fear of movement (TSK ≥35) | >3 months | Virtual reality dodgeball (graded exposure VR game) | 26 （14M/12F) | 23.9±6.8 | 3 sessions, 15 min each, on 3 consecutive days | 7 Day | VAS (pain expectation, MPQ), | Not reported | Not reported | Baseline;7D |
|  |  |  |  |  |  | Active control | 26 人（13M/13F) | 26.7±8.5 |  |  |  |  |  |  |
| 26. | Bennell et al.[26] | 2017 | Australia | Knee osteoarthritis (OA) | ≥3 months | 5 physiotherapy consultations (education, exercise, activity advice) | 84（27M/57F) | 62.0 ± 7.6 | 6–12 calls (30–40 min each) over 6 months | 6 months | NRS pain | WOMAC physical function | AQoL | Baseline, 6 months, 12 months, 18 months |
|  |  |  |  |  |  | Non-active control | 84（35M/49F) | 64.2 ± 7.4 |  |  |  |  |  |  |
| 27. | Hagiwara et al.[27] | 2017 | Japan | Work-related low back pain in hospital staff | ≥3 months, weekly | Wearable lumbosacral support (posture-feedback “Spinal Underwear”) | 54 (2/52) | 44.7±10.0 | Wear it continuously. | 3 months | VAS | Roland-Morris Disability Questionnaire (RMDQ) | Not reported | Baseline, 3 months |
|  |  |  |  |  |  | Non-active control | 53 (1/52) | 44.7±9.6 |  |  |  |  |  |  |
| 28. | Heapy et al.[28] | 2017 | USA | Chronic low back pain | ≥3 months | IVR-CBT (interactive voice response CBT with manual + weekly feedback) | 62 (49M/13F) | 59.2±11.8 | 10 weeks, weekly sessions; daily IVR monitoring | 11W | NRS | Roland-Morris Disability Questionnaire (RMDQ) | SF-36V PCS; SF-36V MCS | Baseline, 3 months, 6 months, 9 months |
|  |  |  |  |  |  | Active control | 63 (48M/15F) | 56.7±11.5 |  |  |  |  |  |  |
| 29. | Lee et.al.[29] | 2017 | South Korea | Chronic neck pain | ≥6 months | App-based Exercise Group | 11 | 27.09±4.83 | 10–15 min/day, 2 days/week, | 8 weeks | VAS | Neck Disability Index (NDI) | SF-36 PCS; SF-36 MCS | Baseline, 8W |
|  |  |  |  |  |  | Non-active control | 9 | 27.56±4.67 |  |  |  |  |  |  |
| 30. | Bahat et al.[30] | 2018 | Australia | Chronic neck pain | >3 months | Exp1: VR-based kinematic training (home-based) | 30 | 48 (38.5, 57.5) | 20 min/day (5 min × 4), 4 days/week, | 4 weeks | VAS | Neck Disability Index (NDI) | EQ-5D | Baseline, 4 weeks; 3-month |
|  |  |  |  |  |  | Active control | 30 | 48 (35, 59) |  |  |  |  |  |  |
| 31. | Chhabra et al.[31] | 2018 | India | Chronic low back pain (CLBP) | ≥3 months | Smartphone App (Snapcare) | 45 | App: 41.4 ± 14.2; | NA | 12W | NPRS | Roland-Morris Disability Questionnaire (RMDQ) | Not reported | Baseline; 12 Week |
|  |  |  |  |  |  | Non-active control | 48 | 41.0±14.2 |  |  |  |  |  |  |
| 32. | Rutledge et al.[32] | 2018 | USA | Chronic low back pain (degenerative disc disease) | ≥ 6 months | Telephone-delivered CBT | 33 | 54.0 ± 14.8 | 11 telephone sessions of 30 minutes each. | 8 weeks | NRS | Roland-Morris Disability Questionnaire (RMDQ) | Not reported | Baseline; 8 Week |
|  |  |  |  |  |  | Active control | 33 | 52.6 ± 12.5 |  |  |  |  |  |  |
| 33. | Yuan, S. L. K.,et al.[33] | 2021 | Brazil | Fibromyalgia syndrome | Defined solely as chronic widespread pain associated with fibromyalgia. | Mobile app–based self-care (ProFibro) | 20 | 43.3 ± 8.4 | NA | 6 weeks; | VAS | FIQ-R function domain | FIQR total score | Baseline; 6W |
|  |  |  |  |  |  | Active control | 20 | 42.1 ± 11.8 |  |  |  |  |  |  |
| 34. | Anita B Amorim et al.[34] | 2019 | Australia | Low Back Pain | Over 12 weeks | Physical Activity + Health Coaching + Mobile Health | 34 （19M/15F） | 59.5±11.9 | 1 face-to-face (1-2h) + 12 phone calls (6 months) | 6 months | NRS | Roland-Morris Disability Questionnaire (RMDQ) | Not reported | Baseline; 6-month |
|  |  |  |  |  |  | Non-active control | 34 （15M/19F） | 57.1±14.9 |  |  |  |  |  |  |
| 35. | Raad Shebib et al.[35] | 2019 | UK | Chronic Non-Specific Low Back Pain (CNSLBP) | ≥3 months | Digital Care Plan (DCP) via an app, including sensor-guided exercise therapy, education, cognitive behavioral therapy, team/individual behavioral coaching, activity and symptom tracking. | 113 | 43±11 | 2 sessions/week | 12 weeks | VAS | Von Korff Disability Score; Oswestry Disability Index (ODI) | Not reported | Baseline ; 12-week |
|  |  |  |  |  |  | Non-active control | 64 | 43±12 |  |  |  |  |  |  |
| 36. | S. Villafaina et al.[36] | 2019 | Spain | Fibromyalgia | Chronic (>3 months) | Intervention based on the “VirtualEx-FM” fitness game | 28 | 54.04 ± 8.45 | 2 sessions/week; 60 min/session | 24 weeks | VAS | Not reported | EQ-5D-5L VAS; EQ-5D-5L utility index | Baseline;24W |
|  |  |  |  |  |  | Non-active control | 27 | 52.72 ± 9.98 |  |  |  |  |  |  |
| 37. | Almhdawi et al.[37] | 2020 | Jordan | Chronic non-specific low back pain | ≥ 3 months | Smartphone app (“Relieve my back”, full version | 21 (7M/14F) | 40.5 ± 7.2 | 1 session per week | 6 weeks, | VAS | Oswestry Disability Index (ODI) | SF-12 | Baseline;  6W |
|  |  |  |  |  |  | Non-active control | 20 (12M/8F) | 41.7 ± 6.4 |  |  |  |  |  |  |
| 38. | Ditchburn JL et al.[38] | 2020 | United Kingdom(UK) | Chronic musculoskeletal pain | ≥12 weeks | Exergaming (IREX®) | 27(7M/20F) | 69.78 ± 4.48 | Twice weekly, 40 min per session | 6 weeks | NPRS | Not reported | Not reported | Baseline;  6W |
|  |  |  |  |  |  | Active control | 27 (5M/22F) | 71.78 ± 6.10 |  |  |  |  |  |  |
| 39. | Greenberg J et al.[39] | 2020 | USA | Chronic musculoskeletal pain | > 3 months | Active control | 41 (18M/23F) | 54.46 ± 14.5 | 10 weekly group sessions, 90 min each | NA | NRS | WHO Disability Assessment Schedule (WHODAS) | Not reported | Baseline;Post |
|  |  |  |  |  |  | GetActive-Fitbit  digital monitoring device | 41 (10M/31F) | 49.07 ± 14.2 |  |  |  |  |  |  |
| 40. | Hinman RS et al.[40] | 2020 | Australia | Knee osteoarthritis | ≥3 months (mean ~9y) | Telephone-delivered physiotherapist-led exercise advice & support | 65 | 62.5 ± 8.1 / | 5–10 telephone consultations with physiotherapists | over 6 months | NRS | WOMAC physical function | AQoL-8D | Baseline;post |
|  |  |  |  |  |  | Non-active control | 110 | 62.4 ± 9.1 |  |  |  |  |  |  |
| 41. | Lam J et al.[41] | 2020 | Sweden | Chronic temporomandibular disorder (TMD) pain | ≥3 months | Internet-based multimodal pain program | 20 (5M/15F); | Median 27 [23–37] years (iMPP); 27 [23–38] years (Control) | 7 modules over ~7 weeks | 7W | GCPS CPI, 0–10 | Not reported | Not reported | Baseline;6M;12M |
|  |  |  |  |  |  | Active control | 23 (4M/19F) |  |  |  |  |  |  |  |
| 42. | Malliaras P et al.[42] | 2020 | Australia | RCRSP (rotator cuff–related shoulder pain) | ≥3 months | telerehabilitation | 36 (4/32) | 53.9 ± 12.0 yrs | 3×/week (two exercises per session; individualized) | 12W | VAS | Shoulder Pain and Disability Index (SPADI) | EQ-5D | Baseline; 6W;12W |
|  |  |  |  |  |  | Non-active control |  |  |  |  |  |  |  |  |
| 43. | Tejera DM et al.[43] | 2020 | Spain | Non-specific chronic neck pain (NS-CNP) | ≥3 months | Virtual Reality+Exercise | 44 (21M / 23F) | 29.7 ± 10.81 | 2 sessions/week | 4 weeks | VAS | Neck Disability Index (NDI) | Not reported | Baseline; 3M |
|  |  |  |  |  |  | Active control |  |  |  |  |  |  |  |  |
| 44. | Sander LB et al.[44] | 2020 | Germany | Chronic low back pain | >= 12 weeks | a Guided Web-Based Self-help Intervention | 933 | 47.8 ± 14.6; | 47min/Once | NA | NRS | Oswestry Disability Index (ODI) | AQoL-6D total score | Baseline;9w;6M;12M |
|  |  |  |  |  |  | Active control | 312 | 48.2 ± 15.1 |  |  |  |  |  |  |
| 45. | Schlicker S et al.[45] | 2020 | Germany | Chronic back pain (CBP) | ≥3 months | App/Web-based guided CBT intervention | N=76 (21 M / 55 F) | 50.8 ± 7.9 years | 1 module/week (45–60 min) +daily SMS reminders optional | 9 weeks, | NRS | ODI functional disability subscale | AQoL-6D | Baseline; post;6M |
|  |  |  |  |  |  | Non-active control |  |  |  |  |  |  |  |  |
| 46. | Suso-Ribera et al.[46] | 2020 | Spain | Chronic musculoskeletal pain | ≥6 months | App-based monitoring (EMA); node A: App + clinical alarms (telemonitoring) | 43 | 52.1 ± 11.2 | 2×/day prompts; alarms flagged to clinician next working day; | App groups: for 30 days; | NRS | Not reported | Not reported | Baseline; post |
|  |  |  |  |  |  | App without alarm | 45 |  |  |  |  |  |  |  |
|  |  |  |  |  |  | Non-active control | 44 |  |  |  |  |  |  |  |
| 47. | Abadiyan F et al.[47] | 2021 | Iran | Chronic nonspecific neck pain with forward head posture (CVA<46°) | ≥3 months | App-assisted GPR (reminder+exercise images; Android) | 19 | 38.5 ± 9.1 | 4×/week, ~50 min/session; App gives acoustic prompts & images | 8 weeks | VAS | Neck Disability Index (NDI) | SF-36 | Baseline; post  ; 8W |
|  |  |  |  |  |  | Non-active control | 20 |  |  |  |  |  |  |  |
| 48. | Dadarkhah A et al.[48] | 2021 | Iran | Chronic nonspecific low back pain (CNLBP) | ≥12 weeks | Remote exercise instruction | 56 (24M / 32F) | 49 ± 8.9 | 2×/day home exercise + phone follow-up (3×/week) | 4 weeks | VAS | Oswestry Disability Index (ODI) | Not reported | Baseline;1M;3M |
|  |  |  |  |  |  | Active control |  |  |  |  |  |  |  |  |
| 49. | Juhlin S et al.[49] | 2021 | Sweden | Chronic widespread pain (CWP) | NA | Person-centred PA plan + eHealth platform | 69 (61 F/8 M) | 48.0 ± 10.2 | 2 in-person meetings then on-demand digital support for 6 months; | 6 months | FIQ-Pain | Not reported | SF-36 PCS | Baseline;6M |
|  |  |  |  |  |  | Active control | 70 (62 F/8 M) | 47.2 ± 10.0 |  |  |  |  |  |  |
| 50. | Nambi G et al.[50] | 2021 | Saudi Arabia / Egypt | Chronic low back pain (cLBP) | ≥ 3 months | Virtual reality training (VRT), | 20 | 23.2 ± 1.5; | 5 days/week | 4 weeks | VAS | Not reported | Not reported | baseline; 4W; 6M |
|  |  |  |  |  |  | Active control | 20 | 23.3 ± 1.5 |  |  |  |  |  |  |
| 51. | Sandal LF et al.[51] | 2021 | Denmark & Norway | Non-specific low back pain (LBP) with disability | ≥ 12 weeks | App-based AI-tailored self-management support (selfBACK app) | 461 (M=206 / F=255) | 47.5 ± 14.7 | weekly tailored advice on physical activity, strength/flexibility, education, plus daily feedback/reminders | 9 months | NRS | Roland-Morris Disability Questionnaire (RMDQ) | EQ-5D weighted | baseline,3M;9M |
|  |  |  |  |  |  | Non-active control |  |  |  |  |  |  |  |  |
| 52. | Lara-Palomo IC et al.[52] | 2022 | Spain | Non-specific chronic low back pain | ≥3 months | E-health program (McKenzie + TENS, video guidance) | 39:17M/22F | 41.9 ± 9.4 | 3 sessions/week | 8 weeks | VAS | Oswestry Disability Index (ODI) | SF-36 Physical function | baseline;post |
|  |  |  |  |  |  | Active control | 35:14M/21F | 54.6 ± 12.9 |  |  |  |  |  |  |
| 53. | Cetin H et al.[53] | 2022 | Turkey | Chronic neck pain (CNP) | ≥6 months | Immersive VR (Oculus Go) added to motor control (MC) exercises | 21(12F/5M) | 40.0 ± 11.9 | 3 sessions/week; each session 40 min | 6 weeks | VAS | ProFitMap-Neck functional limitation index | SF-36 Physical function | baseline;post |
|  |  |  |  |  |  | Active control | 20(11F/6M) | 41.9 ± 10.8 |  |  |  |  |  |  |
| 54. | Gulsen C PT et al.[54] | 2022 | Turkey | Fibromyalgia (FM) | ≥3 months | Fully immersive VR + Exercise | 8 | 38.50 (29.50-50.00) | 2 sessions/week; each session: 30 min aerobic + 10 min rest + 30 min Pilates | 8 weeks; | VAS | Fibromyalgia Impact Questionnaire (FIQ) | SF-36 Mental; SF-36 Physical | baseline;4w；8w |
|  |  |  |  |  |  | Active control | 8 | 46.50 (36.50-49.50) |  |  |  |  |  |  |
| 55. | Eccleston C et al.[55] | 2022 | Finland | Chronic Low Back Pain (CLBP) | ≥3 months | Virtual reality–based psychological intervention (Digital Therapeutics for Pain, DTxP) | 42 (5/37) | 54.69 ± 10.20 | 5 sessions/week; each session 15–60 min; | 6–8 weeks (≈30 sessions) | NRS | Oswestry Disability Index (ODI) | EQ-5D VAS | baseline;5M |
|  |  |  |  |  |  | Non-active control |  |  |  |  |  |  |  |  |
| 56. | Pach D et al.[56] | 2022 | Germany | Chronic neck pain (non-specific) | ≥12 weeks | App-based relaxation (autogenic training, mindfulness meditation, guided imagery) | 220 (M=67 / F=153) | 38.9 ± 11.3 | Daily or ≥5 days/week, 15 min per session | 6 months | NRS | Not reported | Not reported | baseline;3M;6M |
|  |  |  |  |  |  | Non-active control |  |  |  |  |  |  |  |  |
| 57. | Shaygan M et al.[57] | 2022 | Iran | Chronic nonspecific low back pain | ≥3 months | Remote Pain Management Education | 30(25M/5F) | 50.5 ± 10.3 | 7 weekly sessions | 7W | NRS | Not reported | Not reported | baseline;7W;11W |
|  |  |  |  |  |  | Non-active control | 30(20M/10F) |  |  |  |  |  |  |  |
| 58. | Stamm O et al.[58] | 2022 | Germany | Chronic back pain | ≥ 6 months | VR exergame multimodal therapy | 11 | 75.0 ± 5.8 | 3×/week, 30 min | 4 weeks (12 sessions) | NRS | Hannover Functional Ability Questionnaire (FFbH-R) | SF-12 | baseline;post |
|  |  |  |  |  |  | Active control | 11 | 75.5 ± 4.4 |  |  |  |  |  |  |
| 59. | Weise, H. et al.[59] | 2022 | Germany | Nonspecific & degenerative back pain | NA | App-based digital home exercise (ViViRA) | 108 | 57.4 ± 13.8 | daily, 4 exercises/day | 12 weeks | NRS | Not reported | Not reported | baseline;2W;6W;12W |
|  |  |  |  |  |  | Active control | 105 | 57.3 ± 13.5 y |  |  |  |  |  |  |
| 60. | Yeh, C. H.et al.[60] | 2022 | USA | Chronic musculoskeletal pain (CMP) | ≥3 months | Smartphone-based auricular point acupressure (mAPA) | 14 (3M/11F) | 50.4 ± 20.70 | 4 cycles × 5 days on, 2 days off | 4 weeks | NRS | Roland-Morris Disability Questionnaire (RMDQ) | Not reported | baseline;post;1M |
|  |  |  |  |  |  | Non-active control | 11 (3M/8F) | 50.1 ± 13.95 |  |  |  |  |  |  |
| 61. | Cui, D. et al.[61] | 2023 | USA | Chronic low back pain (CLBP) | ≥ 3 months | Multimodal digital care program | 70(24M / 46F) | 50.5 (IQR 22.0) | Exercise 3 times a week, and have a video/phone follow-up once every 4 weeks. | 8 weeks | NRS | Oswestry Disability Index (ODI) | Not reported | baseline;4W;8W |
|  |  |  |  |  |  | Active control | 70(21M / 49F) | 54.5 (IQR 20.0) |  |  |  |  |  |  |
| 62. | Emedoli,D.et al.[62] | 2024 | Italy | Persistent nonspecific neck pain (NP) | ≥12 weeks | VRRS screen projection | 21 | 49.38 ± 10.53 | 12 sessions (45 min, 2×/week | 6weeks | NRS | Neck Disability Index (NDI) | Not reported | baseline;post;6W |
|  |  |  |  |  |  | Active control | 19 | 46.05 ± 10.49 |  |  |  |  |  |  |
| 63. | Maddox, T. et al.[63] | 2023 | USA | Chronic low back pain (cLBP) | ≥3 months | VR behavioral skills program (RelieVRx) | 1067 (772F / 293M / 2 other) | 50.8 ± 13.2 | Once a day (recommended), seven times a week. | 8 weeks, | BPI | Oswestry Disability Index (ODI) | Not reported | baseline;8W |
|  |  |  |  |  |  | Non-active control |  |  |  |  |  |  |  |  |
| 64. | Marcuzzi, A et al.[64] | 2023 | Norway | Neck and/or low back pain (mixed) | Chronic ≥3 months | AI-based personalized self-management smartphone app (SELFBACK) | 294 (121/173) | 50.6 ± 14.9 y | App/e-Help access ad lib with weekly plans; primary evaluation at 3 months (follow-ups at 6 weeks & 6 months) | NA | NRS | Musculoskeletal Health Questionnaire (MSK-HQ) | EQ-5D | baseline;6W;3M;6M |
|  |  |  |  |  |  | Non-active control |  |  |  |  |  |  |  |  |
| 65. | Özden, F. et al.[65] | 2023 | Turkey | Chronic non-radicular neck pain | ≥3 months | Telerehab (TR): Video-guided family sports + Education (online push/message) | 40 (11/29)；TR=20，SR=20 | 44.82 ± 11.87 y | Once a day (for both groups, it means “performing 10 repetitions of the specified exercise daily”); there is no fixed frequency requirement per week. | 8 weeks | VAS | Neck Disability Index (NDI) | SF-36 | baseline;Post;8W |
|  |  |  |  |  |  | Active control |  |  |  |  |  |  |  |  |
| 66. | Park, C.et al.[66] | 2023 | South Korea | chronic low back pain（LBP） | ≥3 M | DPT (AI Digital Application) | 50(30M / 20F) | 37.11±8.33 | 30 minutes each time, 3 times a week, | 4 weeks | NPRS | Oswestry Disability Index (ODI) | SF-12 | baseline;4W |
|  |  |  |  |  |  | Active control | 50(30M / 20F) | 33.21±6.11 |  |  |  |  |  |  |
| 67. | Villatoro-Luque, F. J. et al.[67] | 2023 | Spain | Non-specific low back pain (NLBP) | ≥12 weeks | telerehabilitation (home video-guided exercise + pain neurophysiological education) | 71 (35/36) | 43.4 ± 10.7 | 2×/week | 8 weeks | VAS | Oswestry Disability Index (ODI) | Not reported | baseline;8W;3M |
|  |  |  |  |  |  | Active control |  |  |  |  |  |  |  |  |
| 68. | Guerra-Arencibia, L.et al.[68] | 2024 | Spain | Non-specific chronic neck pain (NCNP) | ≥3 months | Telerehabilitation-based exercise (TRG) | 36 (13M / 23F) | 42.31 ± 13.68 | Three times a week | 8 weeks | VAS | Neck Disability Index (NDI) | SF-12 Physical factor | baseline;8W |
|  |  |  |  |  |  | Active control |  |  |  |  |  |  |  |  |
| 69. | Guo, Q. et al.[69] | 2024 | China | Chronic non-specific neck pain | ≥3 months | VR (iVR)+conventional rehab | 32(7M / 25F) | 35.94 ± 11.02 | 2-3 times per week (a total of 10 sessions, to be completed within 4 weeks) | 4 weeks; | NRS | Neck Disability Index (NDI) | Not reported | baseline;4W;3M |
|  |  |  |  |  |  | Active control | 32(10M / 22F) | 40.09 ± 11.97 |  |  |  |  |  |  |
| 70. | Hinman, R. S.et al.[70] | 2024 | Australia | Chronic knee pain consistent with OA | ≥3 months | Telerehabilitation (Zoom video consultation) | 190 (68/122/0) | 60.5 ± 8.6 | A total of 5 consultations within 3 months. | 3 months | NRS | WOMAC physical function | AQoL-6D | baseline;3M;9M |
|  |  |  |  |  |  | Active control | 204 (56/147/1), | 62.2 ± 8.5; |  |  |  |  |  |  |
| 71. | Kang, D. H. et al.[71] | 2024 | South Korea | Chronic low back pain (CLBP) | ≥12 weeks | Digital therapeutics (MORA Cure LBP, integrating CBT + exercise via smartphone app) | 20 | 38.1 ± 10.0 | 1 session of CBT per week, plus daily exercise (a total of 6 days per week). | 8 weeks | NRS | Oswestry Disability Index (ODI) | EQ-5D | baseline;4W;8W;12W |
|  |  |  |  |  |  | Active control | 23 | 38.5 ± 7.4 |  |  |  |  |  |  |
| 72. | Maddox, T.et al.[72] | 2024 | USA | Chronic low back pain (CLBP) | >3 months | VR-assisted multimodal behavioral skill intervention | 546 | 50.8±13.2 | Daily 1 session, 2–16 min, | for 56 days (8 weeks) | Pain intensity (BPI) | Oswestry Disability Index (ODI) | Not reported | baseline;1, 2, 3, 6, 12 months |
|  |  |  |  |  |  | Non-active control | 547 |  |  |  |  |  |  |  |
| 73. | Mbada et al.[73] | 2024 | Nigeria | Chronic non-specific low back pain (NSLBP) | ≥3 months | （Tele-rehabilitation-based McKenzie Therapy） | 28 (M: 10, F: 18) | 47.6 ± 11.5 | 3 sessions/week × | 8 weeks | VAS | Roland-Morris Disability Questionnaire (RMDQ) | SF-12 PHD / Physical Health Domain | baseline;4W;8W |
|  |  |  |  |  |  | Active control | 21 (M: 6, F: 15) | 43.4 ± 8.2 |  |  |  |  |  |  |
| 74. | Moutzouri, M. et al.[74] | 2024 | Greece | Knee osteoarthritis (KOA) | ≥3 months | BWR-OPA (Online guidance) | 22(3M / 19F) | 65.1 ± 5.3 | 5 times a week (2 video training sessions + 1 consultation session + 3 outdoor walks). | 6 weeks | NPRS | KOOS ADL | KOOS QoL | baseline;6W;12W |
|  |  |  |  |  |  | Active control | 22(7M / 15F) | 63.5 ± 5.6 |  |  |  |  |  |  |
| 75. | Nilmart, P.et al.[75] | 2024 | Thailand | Patellofemoral Pain | ≥3 months | Telehealth-based therapeutic exercise (TTE, real-time video supervised) | 21 | 20.4 ± 1.2 | 3×/week,(12 sessions, ~60 min each) | 4 weeks | VAS | Kujala Patellofemoral Questionnaire (KPQ) | Not reported | baseline;4W |
|  |  |  |  |  |  | Non-active control | 21 | 20.1 ± 1.1 |  |  |  |  |  |  |
| 76. | Okudan, B.et al.[76] | 2024 | Turkey | Facet Joint Arthrosis (FJA) | ≥3 months | Telerehabilitation interventions | 15 | 52.00 ± 4.86 | twice a week | 6 weeks | NPRS | Oswestry Disability Index (ODI) | SF-12 | baseline;6W |
|  |  |  |  |  |  | Non-active control | 15 |  |  |  |  |  |  |  |
| 77. | Onan, D., et al.[77] | 2024 | Turkey | Nonspecific Chronic Neck Pain | ≥3 months | telerehabilitation Spinal Stabilization Exercises (SSE) | 26(13 M / 13 F) | 36.19 ± 9.98 | 45 minutes/session, 3 times/Weeks | 8 weeks | VAS | Neck Disability Index (NDI) | Not reported | baseline;8W |
|  |  |  |  |  |  | Active control |  |  |  |  |  |  |  |  |
| 78. | Shi, W. et al.[78] | 2024 | China | Nonspecific Low Back Pain (NLBP) | ≥3 months | Telerehabilitation-based exercise (TBEG) vs Outpatient-based exercise (OBEG) | 27 | 38.23 (SD 11.55) | 3 times/week, 30 minutes/session | 8 weeks, | NPRS | Oswestry Disability Index (ODI) | SF-36 Physical functioning | Baseline: Week 0, Week 2, Week 4, Week 8 |
|  |  |  |  |  |  | Active control | 27 |  |  |  |  |  |  |  |
| 79. | Barbosa, J. C.et al.[79] | 2025 | Brazil | Chronic non-specific neck pain | ≥12 weeks | Telerehab exercise (video calls) + digital booklet | 35 | 29 ± 8.8 | Twice a week. | 6W | NRS | Neck Disability Index (NDI) | SF-12 | Baseline, 6 weeks; 3 months |
|  |  |  |  |  |  | Non-active control | 35 |  |  |  |  |  |  |  |
| 80. | Cetin, H.et al.[80] | 2025 | Turkey | Chronic neck pain | ≥3 months | VR + traditional sports | 15 (11/4); | 26.9±7.9 | 2 to 3 times per week, for a total of 10 sessions | 4 weeks | VAS | Neck Disability Index (NDI) | SF-36 physical functioning | baseline;4W |
|  |  |  |  |  |  | Active control | 13 | 27.7±6.3 |  |  |  |  |  |  |
| 81. | Chittaro, L.et al.[81] | 2025 | Italy | Fibromyalgia（FM） | Chronic； | Immersive VR + multisensor biofeedback（IVR-BF） vs wait-list control | 10 | 44.00（41.75-47.75） | Complete 5 tasks within 10 days, with a maximum of 1 task per day. | 10 days | VAS | Fibromyalgia Impact Questionnaire (FIQ) | Not reported | Baseline, immediately after the intervention (after completion of the 5 sessions), and 15-day follow-up. |
|  |  |  |  |  |  | Non-active control | 10 | 49.00（47.00-49.00） |  |  |  |  |  |  |
| 82. | Hayashi, K.et al.[82] | 2025 | Japan | Chronic musculoskeletal pain | ≥6 months | Wearable + telemedicine (therapist guidance) : Fitbit Inspire HR continuous wearing + weekly remote follow-up/education | 37 (8/29) | 56.5 ± 14.7 | 1 remote supervision session per week, | 6 months | NRS | Pain Disability Assessment Scale (PDAS) | EQ-5D-3L | Baseline, 1 month, 3 months, 6 months |
|  |  |  |  |  |  | Non-active control | 34 (8/26) | 56.6 ± 14.3 |  |  |  |  |  |  |
| 83. | Sakuma, S.et al.[83] | 2025 | Japan | Chronic musculoskeletal pain | ≥3 months; | Immersive VR (Oculus Quest) | 32 (18/14) | 52.6 ± 10.9 | Once a week. | 3 months | NRS | Not reported | EQ-5D-5L | Baseline, 1 month, 3 months |
|  |  |  |  |  |  | Active control | 34 (14/20) | 56.8 ± 14.1 |  |  |  |  |  |  |
| 84. | Yu Jin Im et al.[84] | 2025 | Republic of Korea | Chronic knee osteoarthritis | ≥3 months | DHI – Mobile App | 29 (5M/24F) | 70.4±6.6 | Daily, 30 min/session | 6 weeks | NRS | WOMAC physical function | SF-36 total score | 6 weeks |
|  |  |  |  |  |  | Non-active control |  |  |  |  |  |  |  |  |
| 85. | Úbeda-D’Ocasar et al.[85] | 2026 | Spain | Fibromyalgia + neck pain | ≥3 months | VR | 54 (0M/54F) | 54.26±7.7 | 2×/week | 4 weeks | VAS | Roland-Morris Disability Questionnaire (RMDQ) | SF-36V PCS | 1 month |
|  |  |  |  |  |  | Non-active control |  |  |  |  |  |  |  |  |
| 86. | Valerie Dieter et al.[86] | 2025 | Germany | Knee osteoarthritis | Chronic | DHI – App + Wearable | 194 (62M/132F) | 61.9±7.7 | 3×/week, 30 min | 12 weeks | KOOS | KOOS ADL | VR-12 PCS | 12 weeks |
|  |  |  |  |  |  | Non-active control |  |  |  |  |  |  |  |  |
| 87. | Shibili Nuhmani et al.[87] | 2025 | Saudi Arabia | WRMDs | Chronic | DHI – Telerehab | 32 (0M/32F) | 28.53±4.31 | 3×/week | 6 weeks | NPRS, SF-36, JSS | Not reported | SF-36 (Total Score) | 6 weeks |
|  |  |  |  |  |  | Non-active control |  |  |  |  |  |  |  |  |
| 88. | Emrullah Alkan et al.[88] | 2025 | Turkey | Chronic non-specific neck pain (CNP) in dentists | ≥3 months (chronic) | TG: Digital Health Intervention (DHI) – Remote video rehabilitation | TG: 19 (3M/14F) | 35.20±4.16 (overall) | 3 times/week (Mon/Wed/Fri) | 12 weeks | VAS | Neck Disability Index (NDI) | Not reported | Post-intervention (12 weeks, no long-term follow-up) |
|  |  |  |  |  |  | Non-active control | CG: 17 (4M/13F) |  |  |  |  |  |  |  |
| 89. | Patcharin .et al[89] | 2025 | Thailand | Chronic non-specific neck pain (CNP) | ≥12 weeks (chronic) | Telerehabilitation group: Digital Health Intervention (DHI) – Remote rehabilitation | 25 (4M/21F) | 19.80±1.29 | 3 times/week | 6 weeks | VAS | Neck Disability Index (NDI) | Not reported | 2 weeks (post-intervention follow-up) |
|  |  |  |  |  |  | Active control | 25 (4M/21F) | 19.76±1.27 |  |  |  |  |  |  |
| 90. | Menekseoglu, A. K.et al.[90] | 2025 | Turkey | Chronic non-specific low back pain (CNSLBP) | ≥12 weeks (chronic) | VR group: Digital Health Intervention (DHI) – VR + hypnotherapy | 30 (9M/21F) | 48.83±11.77 | 5 times/week | 3 weeks | VAS | Roland-Morris Disability Questionnaire (RMDQ) | EQ-5D-5L | 6 weeks |
|  |  |  |  |  |  | Non-active control | 30 (11M/19F) | 46.40±9.57 |  |  |  |  |  |  |
| 91. | LynnL.DeBar et al.[91] | 2025 | USA | High-impact chronic musculoskeletal pain (CMP) | ≥3 months (chronic, median 7 years, IQR 2-25 years) | T-CBT | 1800(NA) | 58.8±14.3 | ~1 session/week (8 sessions total) | 12 weeks | BPI | PROMIS Physical Function Short Form 6b | Not reported | 3 months (primary), 6 months, 12 months |
|  |  |  |  |  |  | APP-CBT |  |  |  |  |  |  |  |  |
|  |  |  |  |  |  | Non-active control |  |  |  |  |  |  |  |  |
| 92. | Fulden Sari et al.[92] | 2025 | Turkey | Fibromyalgia Syndrome (FMS) | Median 5-8 years (chronic, ≥3 months) | Tele-yoga group | 17 (0M/17F) | 37.59±5.71 | 1 session/week (60 min/session) | 4 weeks | (NRS) | Fibromyalgia Impact Questionnaire (FIQ) | Not reported | 4 weeks |
|  |  |  |  |  |  | Non-active control | 14 (0M/14F) | 42.21±7.62 |  |  |  |  |  |  |

References

1.	Zhu D, Zhao J, Wu T, Zhu B, Wang M, Han T. Effects of a Computer Vision-Based Exercise Application for People With Knee Osteoarthritis: Randomized Controlled Trial. JMIR Mhealth Uhealth. 2025 2025/05/12/;13:e63022. doi: 10.2196/63022.

2.	Östlind E, Eek F, Stigmar K, Ekvall Hansson E. Effects of self-monitoring physical activity with wearable activity trackers on perceived joint function and health-related quality of life in people with hip and knee osteoarthritis: a secondary analysis of a cluster-randomised clinical trial. BMC Musculoskelet Disord. 2025 2025/01/09/;26(1):33. doi: 10.1186/s12891-024-08238-8.

3.	Lo HHM, Fong PYH, Wang B, Fung CL, Wong SY, Sit RWS. Clinical Efficacy of Virtual Reality Cave Automatic Virtual Environments (CAVE) for Chronic Musculoskeletal Pain in Older Adults: A Randomized Controlled Trial. J Am Med Dir Assoc. 2025 2025/01//;26(1):105344. doi: 10.1016/j.jamda.2024.105344.

4.	Lindner N, Kornder N, Heisig J, Becker A, der Wardt VV, Viniol A. Digitally supported physical activity counselling for people with chronic back pain: a randomised controlled parallel feasibility study. BMC Prim Care. 2025 2025/02/27/;26(1):58. doi: 10.1186/s12875-025-02742-z.

5.	Darcy R, Couri J, Newkirk K, Neagu R, Darbhe V, Jayabalan P. Impact of a Sensor-Based Platform on the Outcome of Medial Off-Loader Bracing in Individuals With Knee Osteoarthritis: A Pilot Randomized Clinical Trial. Am J Phys Med Rehabil. 2025 2025/02/01/;104(2):155-62. doi: 10.1097/phm.0000000000002560.

6.	Özden F, Güçlü B, Tümtürk İ, Doğrukök Ö N, İmerci A, Tuğay BU. The effect of visual feedback-based clinical monitoring application in patients with chronic low back pain: a randomized controlled trial. Eur Spine J. 2024 2024/02//;33(2):505-16. doi: 10.1007/s00586-023-08098-1.

7.	Nishitha K, Anitha A, Thaheera D. Effectiveness of Virtual Reality-based Rehabilitation and High-intensity Exercise Program for Total Knee Arthroplasty Patients: A Randomised Controlled Trial. Journal of Clinical and Diagnostic Research. 2024 2024;18(11):YC01-YC8. doi: 10.7860/JCDR/2024/71122.20263.

8.	Kim WD, Shin D. Comparison of Outcomes of Physical Therapy Exercises Combined with Either a Video-Based Smartphone Application System or a Written Exercise Program Handout in 34 Patients with Non-Specific Neck Pain. Med Sci Monit. 2024 2024/08/31/;30:e945349. doi: 10.12659/msm.945349.

9.	Dieter V, Janssen P, Krauss I. Efficacy of the mHealth-Based Exercise Intervention re.flex for Patients With Knee Osteoarthritis: Pilot Randomized Controlled Trial. JMIR Mhealth Uhealth. 2024 2024/09/09/;12:e54356. doi: 10.2196/54356.

10.	Weber F, Stuhrmann S, Blum Y, Kloek C, Veenhof C, Grüneberg C. The Usability And Preliminary Effectiveness Of An App-Based Training And Education Program (Join2move) For People With Hip Or Knee Osteoarthritis: A Pilot Randomized Controlled Trial. Osteoarthritis and Cartilage. 2023 2023;31:S79. doi: 10.1016/j.joca.2023.01.021.

11.	Tore NG, Oskay D, Haznedaroglu S. The quality of physiotherapy and rehabilitation program and the effect of telerehabilitation on patients with knee osteoarthritis. Clin Rheumatol. 2023 2023/03//;42(3):903-15. doi: 10.1007/s10067-022-06417-3.

12.	Thiengwittayaporn S, Wattanapreechanon P, Sakon P, Peethong A, Ratisoontorn N, Charoenphandhu N, et al. Development of a mobile application to improve exercise accuracy and quality of life in knee osteoarthritis patients: a randomized controlled trial. Arch Orthop Trauma Surg. 2023 2023/02//;143(2):729-38. doi: 10.1007/s00402-021-04149-8.

13.	Rodríguez Sánchez-Laulhé P, Biscarri-Carbonero Á, Suero-Pineda A, Luque-Romero LG, Barrero García FJ, Blanquero J, et al. The effects of a mobile app-delivered intervention in people with symptomatic hand osteoarthritis: a pragmatic randomized controlled trial. Eur J Phys Rehabil Med. 2023 2023/02//;59(1):54-64. doi: 10.23736/s1973-9087.22.07744-9.

14.	Özlü A, Ünver G, Tuna H, Menekşeoğlu AK. The Effect of a Virtual Reality-Mediated Gamified Rehabilitation Program on Pain, Disability, Function, and Balance in Knee Osteoarthritis: A Prospective Randomized Controlled Study. Games Health J. 2023 2023/04//;12(2):118-24. doi: 10.1089/g4h.2022.0130.

15.	Lee EL, Jang MH, Lee BJ, Han SH, Lee HM, Choi SU, et al. Home-Based Remote Rehabilitation Leads to Superior Outcomes for Older Women With Knee Osteoarthritis: A Randomized Controlled Trial. J Am Med Dir Assoc. 2023 2023/10//;24(10):1555-61. doi: 10.1016/j.jamda.2023.08.013.

16.	Groenveld TD, Smits MLM, Knoop J, Kallewaard JW, Staal JB, de Vries M, et al. Effect of a Behavioral Therapy-Based Virtual Reality Application on Quality of Life in Chronic Low Back Pain. Clin J Pain. 2023 2023/06/01/;39(6):278-85. doi: 10.1097/ajp.0000000000001110.

17.	Hernando-Garijo I, Ceballos-Laita L, Mingo-Gómez MT, Medrano-de-la-Fuente R, Estébanez-de-Miguel E, Martínez-Pérez MN, et al. Immediate Effects of a Telerehabilitation Program Based on Aerobic Exercise in Women with Fibromyalgia. Int J Environ Res Public Health. 2021 2021/02/20/;18(4). doi: 10.3390/ijerph18042075.

18.	Gohir SA, Eek F, Kelly A, Abhishek A, Valdes AM. Effectiveness of Internet-Based Exercises Aimed at Treating Knee Osteoarthritis: The iBEAT-OA Randomized Clinical Trial. JAMA Network Open. 2021 2021;4(2). doi: 10.1001/jamanetworkopen.2021.0012.

19.	Li LC, Feehan LM, Xie H, Lu N, Shaw CD, Gromala D, et al. Effects of a 12-Week Multifaceted Wearable-Based Program for People With Knee Osteoarthritis: Randomized Controlled Trial. JMIR Mhealth Uhealth. 2020 2020/07/03/;8(7):e19116. doi: 10.2196/19116.

20.	Petrozzi MJ, Leaver A, Ferreira PH, Rubinstein SM, Jones MK, Mackey MG. Addition of MoodGYM to physical treatments for chronic low back pain: A randomized controlled trial. Chiropr Man Therap. 2019 2019;27:54. doi: 10.1186/s12998-019-0277-4.

21.	I R, M R, S E, S K, A RZ. A Novel Virtual Reality Technique (Cervigame®) Compared to Conventional Proprioceptive Training to Treat Neck Pain: A Randomized Controlled Trial. J Biomed Phys Eng. 2019 2019/06//;9(3):355-66. doi: 10.31661/jbpe.v0i0.556.

22.	Irvine AB, Russell H, Manocchia M, Mino DE, Cox Glassen T, Morgan R, et al. Mobile-Web app to self-manage low back pain: randomized controlled trial. J Med Internet Res. 2015 2015/01/02/;17(1):e1. doi: 10.2196/jmir.3130.

23.	Sarig Bahat H, Takasaki H, Chen X, Bet-Or Y, Treleaven J. Cervical kinematic training with and without interactive VR training for chronic neck pain - a randomized clinical trial. Man Ther. 2015 2015/02//;20(1):68-78. doi: 10.1016/j.math.2014.06.008.

24.	Rini C, Porter LS, Somers TJ, McKee DC, DeVellis RF, Smith M, et al. Automated Internet-based pain coping skills training to manage osteoarthritis pain: a randomized controlled trial. Pain. 2015 May;156(5):837-48. PMID: 25734997. doi: 10.1097/j.pain.0000000000000121.

25.	Thomas JS, France CR, Applegate ME, Leitkam ST, Walkowski S. Feasibility and Safety of a Virtual Reality Dodgeball Intervention for Chronic Low Back Pain: A Randomized Clinical Trial. J Pain. 2016 Dec;17(12):1302-17. PMID: 27616607. doi: 10.1016/j.jpain.2016.08.011.

26.	Bennell KL, Campbell PK, Egerton T, Metcalf B, Kasza J, Forbes A, et al. Telephone Coaching to Enhance a Home-Based Physical Activity Program for Knee Osteoarthritis: A Randomized Clinical Trial. Arthritis Care Res (Hoboken). 2017 Jan;69(1):84-94. PMID: 27111441. doi: 10.1002/acr.22915.

27.	Hagiwara Y, Yabe Y, Yamada H, Watanabe T, Kanazawa K, Koide M, et al. Effects of a wearable type lumbosacral support for low back pain among hospital workers: A randomized controlled trial. J Occup Health. 2017 Mar 28;59(2):201-9. PMID: 28132971. doi: 10.1539/joh.16-0203-OA.

28.	Heapy AA, Higgins DM, Goulet JL, LaChappelle KM, Driscoll MA, Czlapinski RA, et al. Interactive Voice Response-Based Self-management for Chronic Back Pain: The COPES Noninferiority Randomized Trial. JAMA Intern Med. 2017 Jun 1;177(6):765-73. PMID: 28384682. doi: 10.1001/jamainternmed.2017.0223.

29.	Lee J, Lee M, Lim T, Kim T, Kim S, Suh D, et al. Effectiveness of an application-based neck exercise as a pain management tool for office workers with chronic neck pain and functional disability: A pilot randomized trial. Eur J Integr Med. 2017 Jun;12:87-92. PMID: WOS:000414230000012. doi: 10.1016/j.eujim.2017.04.012.

30.	Sarig Bahat H, Croft K, Carter C, Hoddinott A, Sprecher E, Treleaven J. Remote kinematic training for patients with chronic neck pain: a randomised controlled trial. Eur Spine J. 2018 Jun;27(6):1309-23. PMID: 29018956. doi: 10.1007/s00586-017-5323-0.

31.	Chhabra HS, Sharma S, Verma S. Smartphone app in self-management of chronic low back pain: a randomized controlled trial. Eur Spine J. 2018 Nov;27(11):2862-74. PMID: 30324496. doi: 10.1007/s00586-018-5788-5.

32.	Rutledge T, Atkinson JH, Chircop-Rollick T, D'Andrea J, Garfin S, Patel S, et al. Randomized Controlled Trial of Telephone-delivered Cognitive Behavioral Therapy Versus Supportive Care for Chronic Back Pain. Clin J Pain. 2018 Apr;34(4):322-7. PMID: 28877139. doi: 10.1097/ajp.0000000000000555.

33.	Yuan SLK, Couto LA, Marques AP. Effects of a six-week mobile app versus paper book intervention on quality of life, symptoms, and self-care in patients with fibromyalgia: a randomized parallel trial. Braz J Phys Ther. 2021 Jul-Aug;25(4):428-36. PMID: 33248904. doi: 10.1016/j.bjpt.2020.10.003.

34.	Amorim AB, Pappas E, Simic M, Ferreira ML, Jennings M, Tiedemann A, et al. Integrating Mobile-health, health coaching, and physical activity to reduce the burden of chronic low back pain trial (IMPACT): a pilot randomised controlled trial. BMC Musculoskelet Disord. 2019 Feb 11;20(1):71. PMID: 30744606. doi: 10.1186/s12891-019-2454-y.

35.	Shebib R, Bailey JF, Smittenaar P, Perez DA, Mecklenburg G, Hunter S. Randomized controlled trial of a 12-week digital care program in improving low back pain. NPJ Digit Med. 2019;2:1. PMID: 31304351. doi: 10.1038/s41746-018-0076-7.

36.	Villafaina S, Collado-Mateo D, Domínguez-Muñoz FJ, Fuentes-García JP, Gusi N. Benefits of 24-Week Exergame Intervention on Health-Related Quality of Life and Pain in Women with Fibromyalgia: A Single-Blind, Randomized Controlled Trial. Games Health J. 2019 Dec;8(6):380-6. PMID: 31259617. doi: 10.1089/g4h.2019.0023.

37.	Almhdawi KA, Obeidat DS, Kanaan SF, Oteir AO, Mansour ZM, Alrabbaei H. Efficacy of an innovative smartphone application for office workers with chronic non-specific low back pain: a pilot randomized controlled trial. Clin Rehabil. 2020 Oct;34(10):1282-91. PMID: 32602362. doi: 10.1177/0269215520937757.

38.	Ditchburn JL, van Schaik P, Dixon J, MacSween A, Martin D. The effects of exergaming on pain, postural control, technology acceptance and flow experience in older people with chronic musculoskeletal pain: a randomised controlled trial. BMC Sports Sci Med Rehabil. 2020;12:63. PMID: 33062284. doi: 10.1186/s13102-020-00211-x.

39.	Greenberg J, Popok PJ, Lin A, Kulich RJ, James P, Macklin EA, et al. A Mind-Body Physical Activity Program for Chronic Pain With or Without a Digital Monitoring Device: Proof-of-Concept Feasibility Randomized Controlled Trial. JMIR Form Res. 2020 Jun 8;4(6):e18703. PMID: 32348281. doi: 10.2196/18703.

40.	Hinman RS, Campbell PK, Lawford BJ, Briggs AM, Gale J, Bills C, et al. Does telephone-delivered exercise advice and support by physiotherapists improve pain and/or function in people with knee osteoarthritis? Telecare randomised controlled trial. Br J Sports Med. 2020 Jul;54(13):790-7. PMID: 31748198. doi: 10.1136/bjsports-2019-101183.

41.	Lam J, Svensson P, Alstergren P. Internet-Based Multimodal Pain Program With Telephone Support for Adults With Chronic Temporomandibular Disorder Pain: Randomized Controlled Pilot Trial. J Med Internet Res. 2020 Oct 13;22(10):e22326. PMID: 33048053. doi: 10.2196/22326.

42.	Malliaras P, Cridland K, Hopmans R, Ashton S, Littlewood C, Page R, et al. Internet and Telerehabilitation-Delivered Management of Rotator Cuff-Related Shoulder Pain (INTEL Trial): Randomized Controlled Pilot and Feasibility Trial. JMIR Mhealth Uhealth. 2020 Nov 18;8(11):e24311. PMID: 33206059. doi: 10.2196/24311.

43.	Tejera DM, Beltran-Alacreu H, Cano-de-la-Cuerda R, Leon Hernández JV, Martín-Pintado-Zugasti A, Calvo-Lobo C, et al. Effects of Virtual Reality versus Exercise on Pain, Functional, Somatosensory and Psychosocial Outcomes in Patients with Non-specific Chronic Neck Pain: A Randomized Clinical Trial. Int J Environ Res Public Health. 2020 2020/08/16/;17(16). doi: 10.3390/ijerph17165950.

44.	Sander LB, Paganini S, Terhorst Y, Schlicker S, Lin JX, Spanhel K, et al. Effectiveness of a Guided Web-Based Self-help Intervention to Prevent Depression in Patients With Persistent Back Pain The PROD-BP Randomized Clinical Trial. JAMA Psychiatry. 2020 Oct;77(10):1001-11. PMID: WOS:000582383300008. doi: 10.1001/jamapsychiatry.2020.1021.

45.	Schlicker S, Baumeister H, Buntrock C, Sander L, Paganini S, Lin J, et al. A Web- and Mobile-Based Intervention for Comorbid, Recurrent Depression in Patients With Chronic Back Pain on Sick Leave (Get.Back): Pilot Randomized Controlled Trial on Feasibility, User Satisfaction, and Effectiveness. JMIR Ment Health. 2020 Apr 15;7(4):e16398. PMID: 32293577. doi: 10.2196/16398.

46.	Suso-Ribera C, Castilla D, Zaragozá I, Mesas Á, Server A, Medel J, et al. Telemonitoring in Chronic Pain Management Using Smartphone Apps: A Randomized Controlled Trial Comparing Usual Assessment against App-Based Monitoring with and without Clinical Alarms. Int J Environ Res Public Health. 2020 Sep 9;17(18). PMID: 32916983. doi: 10.3390/ijerph17186568.

47.	Abadiyan F, Hadadnezhad M, Khosrokiani Z, Letafatkar A, Akhshik H. Adding a smartphone app to global postural re-education to improve neck pain, posture, quality of life, and endurance in people with nonspecific neck pain: a randomized controlled trial. Trials. 2021 Apr 12;22(1):274. PMID: 33845880. doi: 10.1186/s13063-021-05214-8.

48.	Dadarkhah A, Rezaimoghadam F, Najafi S, Mohebi B, Azarakhsh A, Rezasoltani Z. Remote Versus in-Person Exercise Instruction for Chronic Nonspecific Low Back Pain Lasting 12 Weeks or Longer: A Randomized Clinical Trial. J Natl Med Assoc. 2021 Jun;113(3):278-84. PMID: 33349469. doi: 10.1016/j.jnma.2020.11.016.

49.	Juhlin S, Bergenheim A, Gjertsson I, Larsson A, Mannerkorpi K. Physical activity with person-centred guidance supported by a digital platform for persons with chronic widespread pain: A randomized controlled trial. J Rehabil Med. 2021 Apr 1;53(4):jrm00175. PMID: 33576434. doi: 10.2340/16501977-2796.

50.	Nambi G, Abdelbasset WK, Alrawaili SM, Alsubaie SF, Abodonya AM, Saleh AK. Virtual reality or isokinetic training; its effect on pain, kinesiophobia and serum stress hormones in chronic low back pain: A randomized controlled trial. Technol Health Care. 2021;29(1):155-66. PMID: 32831210. doi: 10.3233/thc-202301.

51.	Sandal LF, Bach K, Øverås CK, Svendsen MJ, Dalager T, Stejnicher Drongstrup Jensen J, et al. Effectiveness of App-Delivered, Tailored Self-management Support for Adults With Lower Back Pain-Related Disability: A selfBACK Randomized Clinical Trial. JAMA Intern Med. 2021 Oct 1;181(10):1288-96. PMID: 34338710. doi: 10.1001/jamainternmed.2021.4097.

52.	Lara-Palomo IC, Antequera-Soler E, Matarán-Peñarrocha GA, Fernández-Sánchez M, García-López H, Castro-Sánchez AM, et al. Comparison of the effectiveness of an e-health program versus a home rehabilitation program in patients with chronic low back pain: A double blind randomized controlled trial. Digit Health. 2022 Jan-Dec;8:20552076221074482. PMID: 35111332. doi: 10.1177/20552076221074482.

53.	Cetin H, Kose N, Oge HK. Virtual reality and motor control exercises to treat chronic neck pain: A randomized controlled trial. Musculoskelet Sci Pract. 2022 Dec;62:102636. PMID: 35952621. doi: 10.1016/j.msksp.2022.102636.

54.	Gulsen C Pt M, Soke F Pt P, Eldemir K Pt M, Apaydin Y Pt M, Ozkul C Pt P, Guclu-Gunduz A Pt P, et al. Effect of fully immersive virtual reality treatment combined with exercise in fibromyalgia patients: a randomized controlled trial. Assist Technol. 2022 May 4;34(3):256-63. PMID: 32543290. doi: 10.1080/10400435.2020.1772900.

55.	Eccleston C, Fisher E, Liikkanen S, Sarapohja T, Stenfors C, Jääskeläinen SK, et al. A prospective, double-blind, pilot, randomized, controlled trial of an "embodied" virtual reality intervention for adults with low back pain. Pain. 2022 Sep 1;163(9):1700-15. PMID: 35324507. doi: 10.1097/j.pain.0000000000002617.

56.	Pach D, Blödt S, Wang J, Keller T, Bergmann B, Rogge AA, et al. App-Based Relaxation Exercises for Patients With Chronic Neck Pain: Pragmatic Randomized Trial. JMIR Mhealth Uhealth. 2022 Jan 7;10(1):e31482. PMID: 34994708. doi: 10.2196/31482.

57.	Shaygan M, Jaberi A, Firozian R, Yazdani Z. Comparing the effects of multimedia and face-to-face pain management education on pain intensity and pain catastrophizing among patients with chronic low back pain: A randomized clinical trial. PLoS One. 2022;17(6):e0269785. PMID: 35709207. doi: 10.1371/journal.pone.0269785.

58.	Stamm O, Dahms R, Reithinger N, Ruß A, Müller-Werdan U. Virtual reality exergame for supplementing multimodal pain therapy in older adults with chronic back pain: a randomized controlled pilot study. Virtual Real. 2022;26(4):1291-305. PMID: 35194374. doi: 10.1007/s10055-022-00629-3.

59.	Weise H, Zenner B, Schmiedchen B, Benning L, Bulitta M, Schmitz D, et al. The Effect of an App-Based Home Exercise Program on Self-reported Pain Intensity in Unspecific and Degenerative Back Pain: Pragmatic Open-label Randomized Controlled Trial. J Med Internet Res. 2022 Oct 28;24(10):e41899. PMID: 36215327. doi: 10.2196/41899.

60.	Yeh CH, Kawi J, Grant L, Huang X, Wu H, Hardwicke RL, et al. Self-Guided Smartphone Application to Manage Chronic Musculoskeletal Pain: A Randomized, Controlled Pilot Trial. Int J Environ Res Public Health. 2022 Nov 11;19(22). PMID: 36429591. doi: 10.3390/ijerph192214875.

61.	Cui D, Janela D, Costa F, Molinos M, Areias AC, Moulder RG, et al. Randomized-controlled trial assessing a digital care program versus conventional physiotherapy for chronic low back pain. NPJ Digit Med. 2023 Jul 7;6(1):121. PMID: 37420107. doi: 10.1038/s41746-023-00870-3.

62.	Emedoli D, Alemanno F, Iannaccone S, Houdayer E, Castellazzi P, Zangrillo F, et al. Sensory-motor training with virtual reality as a complementary intervention to manual therapy for persistent non-specific neck pain: a randomized controlled trial. Eur J Phys Rehabil Med. 2024 Aug;60(4):680-90. PMID: 38922315. doi: 10.23736/s1973-9087.24.08115-2.

63.	Maddox T, Oldstone L, Sparks CY, Sackman J, Oyao A, Garcia L, et al. In-Home Virtual Reality Program for Chronic Lower Back Pain: A Randomized Sham-Controlled Effectiveness Trial in a Clinically Severe and Diverse Sample. Mayo Clin Proc Digit Health. 2023 Dec;1(4):563-73. PMID: 40206316. doi: 10.1016/j.mcpdig.2023.09.003.

64.	Marcuzzi A, Nordstoga AL, Bach K, Aasdahl L, Nilsen TIL, Bardal EM, et al. Effect of an Artificial Intelligence-Based Self-Management App on Musculoskeletal Health in Patients With Neck and/or Low Back Pain Referred to Specialist Care: A Randomized Clinical Trial. JAMA Netw Open. 2023 Jun 1;6(6):e2320400. PMID: 37368401. doi: 10.1001/jamanetworkopen.2023.20400.

65.	Özden F, Özkeskin M, Tümtürk İ, Yalın Kılınç C. The effect of exercise and education combination via telerehabilitation in patients with chronic neck pain: A randomized controlled trial. Int J Med Inform. 2023 Dec;180:105281. PMID: 37924590. doi: 10.1016/j.ijmedinf.2023.105281.

66.	Park C, Yi C, Choi WJ, Lim HS, Yoon HU, You SJH. Long-term effects of deep-learning digital therapeutics on pain, movement control, and preliminary cost-effectiveness in low back pain: A randomized controlled trial. Digit Health. 2023 Jan-Dec;9:20552076231217817. PMID: 38053732. doi: 10.1177/20552076231217817.

67.	Villatoro-Luque FJ, Rodríguez-Almagro D, Aibar-Almazán A, Fernández-Carnero S, Pecos-Martín D, Ibáñez-Vera AJ, et al. In non-specific low back pain, is an exercise program carried out through telerehabilitation as effective as one carried out in a physiotherapy center? A controlled randomized trial. Musculoskelet Sci Pract. 2023 Jun;65:102765. PMID: 37141771. doi: 10.1016/j.msksp.2023.102765.

68.	Guerra-Arencibia L, Santana-Déniz C, Pecos-Martín D, Fernández-Carnero S, de Miguel-Hernando N, Achalandabaso-Ochoa A, et al. Effectiveness of a Telerehabilitation-Based Exercise Program in Patients with Chronic Neck Pain-A Randomized Clinical Trial. Sensors (Basel). 2024 Dec 18;24(24). PMID: 39771803. doi: 10.3390/s24248069.

69.	Guo Q, Zhang L, Han LL, Gui C, Chen G, Ling C, et al. Effects of Virtual Reality Therapy Combined With Conventional Rehabilitation on Pain, Kinematic Function, and Disability in Patients With Chronic Neck Pain: Randomized Controlled Trial. JMIR Serious Games. 2024 Apr 24;12:e42829. PMID: 38656775. doi: 10.2196/42829.

70.	Hinman RS, Campbell PK, Kimp AJ, Russell T, Foster NE, Kasza J, et al. Telerehabilitation consultations with a physiotherapist for chronic knee pain versus in-person consultations in Australia: the PEAK non-inferiority randomised controlled trial. Lancet. 2024 Mar 30;403(10433):1267-78. PMID: 38461844. doi: 10.1016/s0140-6736(23)02630-2.

71.	Kang DH, Park JH, Yoon C, Choi CH, Lee S, Park TH, et al. Multidisciplinary Digital Therapeutics for Chronic Low Back Pain Versus In-Person Therapeutic Exercise with Education: A Randomized Controlled Pilot Study. J Clin Med. 2024 Dec 4;13(23). PMID: 39685835. doi: 10.3390/jcm13237377.

72.	Maddox T, Oldstone L, Sackman J, Maddox R, Adair T, Ffrench K, et al. Twelve-month results for a randomized sham-controlled effectiveness trial of an in-home skills-based virtual reality program for chronic low back pain. Pain Rep. 2024 Oct;9(5):e1182. PMID: 39239633. doi: 10.1097/pr9.0000000000001182.

73.	Mbada C, Olanipekun OO, Ademoyegun A, Egwu MO, Makinde M, Gebrye T, et al. Effectiveness of telerehabilitation-based mckenzie method versus manual therapy in low-back pain management: a randomised controlled trial. Bulletin of Faculty of Physical Therapy. 2024 2024/11/06;29(1):50. doi: 10.1186/s43161-024-00218-2.

74.	Moutzouri M, Koumantakis GA, Hurley M, Kladouchou AG, Gioftsos G. Effectiveness of a Web-Guided Self-Managed Telerehabilitation Program Enhanced with Outdoor Physical Activity on Physical Function, Physical Activity Levels and Pain in Patients with Knee Osteoarthritis: A Randomized Controlled Trial. J Clin Med. 2024 Feb 6;13(4). PMID: 38398248. doi: 10.3390/jcm13040934.

75.	Nilmart P, Vongsirinavarat M, Khawsuwan P, Chumthong K, Tadein R, Komalasari DR. Impact of telehealth-based therapeutic exercise on pain, functional performance and dynamic knee valgus in young adult females with patellofemoral pain: a randomised controlled trial. BMJ Open Sport Exerc Med. 2024;10(4):e001939. PMID: 39720152. doi: 10.1136/bmjsem-2024-001939.

76.	Okudan B, Çelik D, Koban O. The effectiveness of telerehabilitation-based exercise combined with pain neuroscience education for patients with facet joint arthrosis: A randomized controlled study. Eur J Pain. 2024 Mar;28(3):382-95. PMID: 37747343. doi: 10.1002/ejp.2187.

77.	Onan D, Demirci E, Turhan E, Ülger Ö. Spinal stabilization exercises for transversus abdominis and lumbar multifidus thickness via telerehabilitation and face-to-face approaches in patients with nonspecific chronic neck pain: a randomized controlled trial. Turk J Med Sci. 2024 2024;54(4):811-21. doi: 10.55730/1300-0144.5853.

78.	Shi W, Zhang Y, Bian Y, Chen L, Yuan W, Zhang H, et al. The Physical and Psychological Effects of Telerehabilitation-Based Exercise for Patients With Nonspecific Low Back Pain: Prospective Randomized Controlled Trial. JMIR Mhealth Uhealth. 2024 Sep 6;12:e56580. PMID: 39240210. doi: 10.2196/56580.

79.	Barbosa JC, da Luz BV, da Silva BFP, Marques AP, Saragiotto BT, Comachio J, et al. Effectiveness of Telerehabilitation Exercise Programme on Disability and Pain in Patients With Chronic Non-Specific Neck Pain: Randomised Controlled Trial Assessor-Blinded. Musculoskeletal Care. 2025 Jun;23(2):e70119. PMID: 40374317. doi: 10.1002/msc.70119.

80.	Cetin H, Aydogdu S, Onal B, Dulger E, Bilgin S, Oge HK, et al. Immersive virtual reality versus cervical mobilization: The effects on joint position sense and pain for chronic neck pain - a randomized controlled trial. Braz J Phys Ther. 2025 Sep-Oct;29(5):101228. PMID: 40435919. doi: 10.1016/j.bjpt.2025.101228.

81.	Chittaro L, Longhino S, Serafini M, Cacioppo S, Quartuccio L. Efficacy of Immersive Virtual Reality Combined With Multisensor Biofeedback on Chronic Pain in Fibromyalgia: A Pilot Randomized Controlled Trial. ACR Open Rheumatol. 2025 May;7(5):e70048. PMID: 40372182. doi: 10.1002/acr2.70048.

82.	Hayashi K, Miki K, Shiro Y, Tetsunaga T, Takasusuki T, Hosoi M, et al. Utilization of telemedicine in conjunction with wearable devices for patients with chronic musculoskeletal pain: a randomized controlled clinical trial. Sci Rep. 2025 Jan 9;15(1):11. PMID: WOS:001394990600039. doi: 10.1038/s41598-024-85056-x.

83.	Sakuma S, Kimpara K, Kawai Y, Yanagita Y, Tanaka N, Tawara Y, et al. Integrating Physical Therapy and Virtual Reality to Manage Pain-Related Fear of Movement in Patients With Chronic Pain: A Randomized Controlled Trial. Cureus. 2025 Feb;17(2):e79551. PMID: 40151697. doi: 10.7759/cureus.79551.

84.	Im YJ, Choi J, Choi SA, Kwon JY, Do JG. Feasibility and Preliminary Effectiveness of a Mobile App-Based Personalized Exercise Program in Older Patients With Chronic Knee Osteoarthritis: Pilot Randomized Controlled Trial. JMIR Mhealth Uhealth. 2025 Dec 16;13:e71073. PMID: 41401375. doi: 10.2196/71073.

85.	Úbeda-D'Ocasar E, Moreno-Crespo Y, Cimadevilla-Fernández-Pola E, Hernández-Lougedo J, Navas-Mosqueda Á, Caballero-Corella M, et al. Therapeutic Use of Virtual Reality for Patients With Fibromyalgia and Chronic Neck Pain: Randomized Controlled Trial. JMIR Rehabil Assist Technol. 2026 Jan 23;13:e81158. PMID: 41575862. doi: 10.2196/81158.

86.	Dieter V, Martus P, Seißler D, Serna-Higuita LM, Janssen P, Krauss I. Effectiveness of the Self-Directed mHealth Exercise Intervention re.flex in Patients With Knee Osteoarthritis: Randomized Controlled Trial. J Med Internet Res. 2025 Oct 9;27:e71558. PMID: 41066658. doi: 10.2196/71558.

87.	Nuhmani S, AlBakheet A, AlQahtani G, AlDulaijan R, AlHomiyn M, Al Saikhan L. Effectiveness of Telerehabilitation in Reducing Pain and Improving Quality of Life and Job Satisfaction Among Cardiac Sonographers with Work-Related Musculoskeletal Disorders: A Randomized Controlled Trial. J Clin Med. 2025 Sep 18;14(18). PMID: 41010780. doi: 10.3390/jcm14186576.

88.	Alkan E, Gelecek N, Oz İ K, Akcalı A. Effects of combined supervised and telerehabilitation exercise programs on pain and disability in dentists with chronic neck pain: a randomized controlled trial. BMC Musculoskelet Disord. 2025 Aug 7;26(1):759. PMID: 40775626. doi: 10.1186/s12891-025-09010-2.

89.	Nilmart P, Sichuai A, Chedang A, Goontharo C, Janjamsai N. Internet-Based Telerehabilitation Versus in-Person Therapeutic Exercises in Young Adult Females With Chronic Neck Pain and Forward Head Posture: Randomized Controlled Trial. JMIR Rehabil Assist Technol. 2025 Jul 25;12:e74979. PMID: 40712139. doi: 10.2196/74979.

90.	Menekseoglu AK, Asik HK, Sahbaz T, İs EE. Mind over pain: VR-based hypnotherapy for chronic non-specific low back pain - a randomised controlled trial. Ir J Med Sci. 2025 Nov 10. PMID: 41212507. doi: 10.1007/s11845-025-04158-1.

91.	DeBar LL, Mayhew M, Wellman RD, Balderson BH, Dickerson JF, Elder CR, et al. Telehealth and Online Cognitive Behavioral Therapy-Based Treatments for High-Impact Chronic Pain: A Randomized Clinical Trial. Jama. 2025 Jul 23;334(7):592-605. PMID: 40699570. doi: 10.1001/jama.2025.11178.

92.	Sari F, Aran AS, Alp G, Bayram A, Kurt S. The effects of tele-yoga on symptom management and patient perceptions in fibromyalgia syndrome: a randomized controlled trial. J Bodyw Mov Ther. 2025 Dec;45:878-85. PMID: 41316664. doi: 10.1016/j.jbmt.2025.10.010.
