# Supplementary material for: Effects of Digital Health Interventions in Chronic Musculoskeletal Pain: Systematic Review and Bayesian Network Meta-Analysis of Randomized Controlled Trials
Source: J Med Internet Res. 2026 Oct 7;28:e95903. doi: 10.2196/95903 (PMC13645338; doi:10.2196/95903)
Supplement: Supplementary file 6 [file jmir-v28-e95903-s005.docx]

**Multimedia Appendix 5. Descriptive Synthesis of Health-Related Quality-of-Life Outcomes**

**Overview:** Health-related quality-of-life outcomes were synthesized descriptively because the included studies used heterogeneous instruments, domains, scoring systems, and reporting formats. Values are reproduced from the supplied extraction sheet; no pooled effect estimate was calculated in this table.A total of 51 studies reported outcomes related to quality of life, involving 60 study-outcome entries. Due to significant differences in measurement tools, evaluation dimensions, scoring directions, and data reporting methods across studies, quantitative synthesis was not performed; instead, a descriptive summary was used. The most commonly used instruments included EQ-5D and its various versions, KOOS Quality of Life subscale, SF-12, SF-36, and AQoL series scales; some studies also employed FIQ-R/FIQR, Nottingham Health Profile, and VR-12. Some studies reported only physical or mental health subscales without providing overall quality of life scores.

| **Study** | **HRQoL measure** | **Intervention group and reported data** | **Comparator group and reported data** | **Descriptive finding** |
| --- | --- | --- | --- | --- |
| 01. Zhu et al.[1] | AQoL-6D | CV-based graded exercise APP baseline 0.45 ± 0.18; post 0.48 ± 0.19; change +0.03 ± 0.42; n=24 | Active control baseline 0.55 ± 0.19; post 0.52 ± 0.21; change -0.03 ± 0.45; n=17 | The intervention group showed greater numerical improvement than the comparator |
| 02. Östlind et al.[2] | EQ-5D | SOASP + wearable activity tracker baseline 0.78 ± 0.09; post 0.79 ± 0.08; change +0.01 ± 0.75; n=73 | Active control baseline 0.77 ± 0.07; post 0.78 ± 0.09; change +0.01 ± 0.74; n=46 | Both groups showed the same numerical change. |
| 03. Lo et al.[3] | EQ-5D | VR-CAVE with Tai Chi baseline 0.72 ± 0.21; post 0.76 ± 0.19; change +0.04 ± 0.69; n=32 | Active control baseline 0.75 ± 0.19; post 0.76 ± 0.19; change +0.01 ± 0.69; n=33 | The intervention group showed greater numerical improvement than the comparator |
| 04. Lindner et al.[4] | SF-12 PCS | Digitally supported physical activity counselling (ExPa app) baseline 40 ± 4.44; post 33.72 ± 5.19; change -6.28 ± 31.45; n=8 | Non-active control baseline 30 ± 5.93; post 33.6 ± 10.07; change +3.6 ± 29.87; n=12 | The comparator showed greater numerical improvement than the intervention |
|  | SF-12 MCS | Digitally supported physical activity counselling (ExPa app) baseline 39.4 ± 6.03; post 46.2 ± 17.2; change +6.8 ± 40.44; n=14 | Non-active control baseline 44.8 ± 18; post 42.9 ± 17; change -1.9 ± 37.42; n=17 | The intervention group showed greater numerical improvement than the comparator |
| 05. Darcy et al.[5] | KOOS QoL | Medial off-loader brace + sensor-based platform baseline 37.71 ± 18.25; change +9.51 ± 17.09; n=24 | Active control baseline 36.67 ± 17.73; change +6.25 ± 15.88; n=22 | The intervention group showed greater numerical improvement than the comparator |
| 06.Ozden et al.[6] | NHP First Section Total | PhysioAnalyst app + visual feedback baseline 200.19 ± 115.15; change -140.12 ± 121.86; n=22 | Active control baseline 129.98 ± 103.97; change -31.9 ± 73.62; n=22 | The intervention group showed greater numerical improvement than the comparator |
| 09.Dieter et al. [7] | KOOS QoL | Sensor-assisted app-based exercise (re.flex) baseline 38.7 ± 15.7; post 47.6 ± 10.2; change +8.9 ± 43.41; n=26 | Non-active control baseline 39.2 ± 15; post 35.1 ± 10.23; change -4.1 ± 31.27; n=29 | The intervention group showed greater numerical improvement than the comparator |
| 10.Weber et al.[8] | KOOS QoL | Join2Move app (graded physical activity baseline 34.9 ± 19; post 40.4 ± 13; change +5.5 ± 35.72; n=32 | Non-active control baseline 35.5 ± 20.5; post 38.9 ± 23.4; change +3.4 ± 33.92; n=28 | The intervention group showed greater numerical improvement than the comparator |
| 11.Tore et al. (2023)[9] | KOOS QoL | Telerehabilitation(real-time video guidance + exercise) baseline 39.87 ± 16.55; post 69.41 ± 18.9; change +29.54 ± 62.15; n=24 | Active control baseline 40.75 ± 23.61; post 45.45 ± 24.38; change +4.7 ± 39.4; n=24 | The intervention group showed greater numerical improvement than the comparator |
| 12.Thiengwittayaporn et al. [10] | KOOS QoL | "Rak Kao" mobile app (disease assessment + personalized exercise guidance) baseline 69.5 ± 6.2; post 79.6 ± 10.7; change +10.1 ± 74.83; n=42 | Non-active control baseline 64.7 ± 8.7; post 71.2 ± 7.8; change +6.5 ± 67.64; n=40 | The intervention group showed greater numerical improvement than the comparator |
| 16. Groenveld et al.[11] | SF-12 PCS | Behavioral therapy based VR application baseline NR; post 37.6 ± 6.73*; n=20 | Standard care waiting list baseline NR; post 38.4 ± 7.42*; n=20 | Insufficient numerical data for a between-group descriptive comparison. |
|  | SF-12 MCS | Behavioral therapy based VR application baseline NR; post 46.4 ± 7.87*; n=20 | Standard care waiting list baseline NR; post 46 ± 8.67*; n=20 | The study reported no statistically significant change; numerical results are presented descriptively. |
| 17. Hernando-Garijo et al.[12] | FIQ-R total score | Video guided telerehabilitation aerobic exercise baseline 59.44 ± 9.04; post 44 ± 15.21; change -15.44 ± 38.71; n=17 | No additional intervention baseline 55.36 ± 16.46; post 46.9 ± 20.47; change -8.46 ± 40.73; n=17 | The intervention group showed greater numerical improvement than the comparator |
| 19.Li et al. (2020)[13] | KOOS QoL | Wearable multifaceted program (Fitbit Flex-2 + FitViz + PT counseling) baseline 44 ± 16; post 49.3 ± 19.1; change +5.3 ± 43.05; n=19 | Delayed intervention baseline 47.5 ± 16; post 54.4 ± 14.6; change +6.9 ± 48.77; n=24 | The comparator showed greater numerical improvement than the intervention |
| 26.Bennell et al.[14] | AQoL | 5 physiotherapy consultations (education, exercise, activity advice) baseline 0.7 ± 0.1; post 0.8 ± 0.1; change +0.1 ± 0.75; n=66 | Non-active control baseline 0.7 ± 0.1; post 0.8 ± 0.2; change +0.1 ± 0.72; n=62 | Both groups showed the same numerical change. |
| 28. Heapy et al.[15] | SF-36V PCS | Interactive voice response based CBT baseline 36.9 ± 6.7; post 38.4*; change +1.5 ± 6.47; n=45 | In person CBT baseline 33.5 ± 7.8; post 35.59*; change +2.09 ± 6.51; n=41 | The comparator showed greater numerical improvement than the intervention |
|  | SF-36V MCS | Interactive voice response based CBT baseline 48.9 ± 9; post 49.86*; change +0.96 ± 7.89; n=45 | In person CBT baseline 49 ± 8.3; post 47.53*; change -1.47 ± 7.89; n=41 | The intervention group showed greater numerical improvement than the comparator |
| 29. Lee. et al[16] | SF-36 PCS | App-based Exercise Group baseline 43.18 ± 8.58; post 48.4 ± 7.22; change +5.22 ± 45.22; n=11 | Non-active control baseline 49.56 ± 7.42; post 50.88 ± 5.03; change +1.32 ± 48.56; n=9 | The intervention group showed greater numerical improvement than the comparator |
|  | SF-36 MCS | App-based Exercise Group baseline 45.21 ± 7.49; post 45.72 ± 12.37; change +0.51 ± 40.96; n=11 | Non-active control baseline 42.91 ± 13.69; post 44.39 ± 10.1; change +1.48 ± 40.3; n=9 | The comparator showed greater numerical improvement than the intervention |
| 30. Sarig et al[17] | EQ-5D | Exp1: VR-based kinematic training (home-based) baseline 68.19 ± 19.6; post 83.28 ± 14.8; change +15.09 ± 76.95; n=48 | Active control baseline 69.95 ± 17.28; post 75.85 ± 12.6; change +5.9 ± 70.4; n=44 | The intervention group showed greater numerical improvement than the comparator |
| 33. Yuan et al.[18] | FIQR total score | Mobile app–based self-care (ProFibro) baseline 62.6 ± 16.8; post 53.7 ± 17.1; change -8.9 ± 47.52; n=20 | Active control baseline 65.4 ± 18; post 55.5 ± 18.2; change -9.9 ± 49; n=20 | The comparator showed greater numerical improvement than the intervention |
| 36. Villafaina et al.[19] | EQ-5D-5L VAS | VirtualEx-FM exergame baseline 51.61 ± 18.61; post 52.3 ± 17.34; change +0.69 ± 46.14; n=28 | Usual care baseline 59.23 ± 14.74; post 45.88 ± 17.64; change -13.35 ± 40.09; n=26 | The intervention group showed greater numerical improvement than the comparator |
|  | EQ-5D-5L utility index | VirtualEx-FM exergame baseline 0.51 ± 0.25; post 0.56 ± 0.18; change +0.05 ± 0.5; n=28 | Usual care baseline 0.57 ± 0.28; post 0.52 ± 0.24; change -0.05 ± 0.45; n=27 | The intervention group showed greater numerical improvement than the comparator |
| 37.Almhdawi[20] | SF-12 | Smartphone app (“Relieve my back”, full version baseline 61.05 ± 17.54; post 71.35 ± 15.85; change +10.3 ± 64.89; n=20 | Non-active control baseline 64.21 ± 17.84; post 65.53 ± 17.55; change +1.32 ± 58.75; n=19 | The intervention group showed greater numerical improvement than the comparator |
| 40.Hinman[21] | AQoL-8D | Telephone-delivered physiotherapist-led exercise advice & support baseline 0.7 ± 0.2; post 0.7 ± 0.2; change 0 ± 0.62; n=82 | Existing nurse led telephone service baseline 0.7 ± 0.2; post 0.7 ± 0.2; change 0 ± 0.62; n=75 | Both groups showed the same numerical change. |
| 42.Malliaras[22] | EQ-5D | telerehabilitation baseline 0.76 ± 0.11; post 0.73 ± 0.09; change -0.03 ± 0.69; n=10 | baseline 0.74 ± 0.13; post 0.77 ± 0.13; change +0.03 ± 0.71; n=12 | The comparator showed greater numerical improvement than the intervention |
|  | EQ-5D | Non-active control baseline 0.74 ± 0.12; post 0.78 ± 0.07; change +0.04 ± 0.75; n=12 | NR | Comparator data were not available for descriptive comparison. |
| 44. Sander et al.[23] | AQoL-6D total score | Guided web based self help intervention plus treatment as usual baseline 46.15 ± 7.8; post 40.96 ± 7.49; change -5.19 ± 37.78; n=149 | Treatment as usual baseline 45.96 ± 7.46; post 44.39 ± 8.68; change -1.57 ± 40.75; n=146 | The comparator showed greater numerical improvement than the intervention |
| 45.Schlicker[24] | AQoL-6D | App/Web-based guided CBT intervention baseline 0.48 ± 0.16; post 0.6 ± 0.18; change +0.12 ± 0.53; n=40 | Non-active control baseline 0.47 ± 0.16; post 0.55 ± 0.12; change +0.08 ± 0.5; n=36 | The intervention group showed greater numerical improvement than the comparator |
| 47. Abadiyan F et al.[25] | SF-36 | App-assisted GPR (reminder+exercise images; Android) baseline 69.1 ± 4; post 78.5 ± 3.6; change +9.4 ± 76.76; n=19 | Non-active control baseline 69.8 ± 6; post 71.6 ± 7.7; change +1.8 ± 68.08; n=20 | The intervention group showed greater numerical improvement than the comparator |
| 49. Juhlin S et al.[26] | SF-36 PCS | Person-centred PA plan + eHealth platform baseline 21.19 ± 3.73; change +1.09 ± 19.15; n=52 | Active control baseline 21.09 ± 4.52; change +2.54 ± 17.06; n=57 | The comparator showed greater numerical improvement than the intervention |
| 51. Sandal LF et al[27] | EQ-5D weighted | App-based AI-tailored self-management support (selfBACK app) baseline 0.71 ± 0.11; post 0.78 ± 0.13; change +0.07 ± 0.72; n=170 | Non-active control baseline 0.7 ± 0.14; post 0.76 ± 0.14; change +0.06 ± 0.7; n=182 | The intervention group showed greater numerical improvement than the comparator |
| 52. Lara-Palomo IC et al[28] | SF-36 Physical function | E-health program (McKenzie + TENS, video guidance) baseline 76.41 ± 16.58; post 93.08 ± 8.71; change +16.67 ± 89.05; n=39 | Active control baseline 72.14 ± 11.39; post 81.43 ± 12.28; change +9.29 ± 76.04; n=35 | The intervention group showed greater numerical improvement than the comparator |
| 53. Cetin H et al[29] | SF-36 Physical function | Immersive VR (Oculus Go) added to motor control (MC) exercises baseline 73.19 ± 20.21; change +12.29 ± 10.1; n=17 | Active control baseline 75 ± 24.25; change +7.71 ± 14.15; n=17 | The intervention group showed greater numerical improvement than the comparator |
| 54. Gulsen C PT et al.[30] | SF-36 Mental | Fully immersive VR + Exercise baseline 84.73 ± 36.86; post 222.36 ± 56.51; change +137.05 ± 61.88; n=8 | NR | Comparator data were not available for descriptive comparison. |
|  | SF-36 Physical | Active control baseline 71.9 ± 36.41; post 208.04 ± 87.56; change +137.32 ± 93.82 | NR | Comparator data were not available for descriptive comparison. |
|  | SF-36 Mental | Fully immersive VR + Exercise baseline 102.98 ± 133.58; post 172.68 ± 55.51; change +65.94 ± 81.31; n=8 | NR | Comparator data were not available for descriptive comparison. |
|  | SF-36 Physical | Active control baseline 89.21 ± 56.07; post 177.03 ± 85.78; change +91.65 ± 51.15 | NR | Comparator data were not available for descriptive comparison. |
| 55. Eccleston C et al.[31] | EQ-5D VAS | Virtual reality–based psychological intervention (Digital Therapeutics for Pain, DTxP) baseline 47.7 ± 16.8; post 66.1 ± 22.2; change +18.4 ± 58.26; n=9 | Non-active control baseline 55.7 ± 19.6; post 62 ± 23.9; change +6.3 ± 54.16; n=10 | The intervention group showed greater numerical improvement than the comparator |
| 58.Stamm O et al.[32] | SF-12 | VR exergame multimodal therapy baseline 59.03 ± 7.83; post 60.7 ± 8.01; change +1.67 ± 57.12; n=11 | Active control baseline 64.15 ± 7.91; post 62.24 ± 7.27; change -1.91 ± 62.66; n=11 | The intervention group showed greater numerical improvement than the comparator |
| 64.Marcuzzi, A et al.[33] | EQ-5D | AI-based personalized self-management smartphone app (SELFBACK) baseline 0.65 ± 0.17; post 0.7 ± 0.15; change +0.05 ± 0.72; n=99 | Non-active control baseline 0.63 ± 0.14; post 0.66 ± 0.2; change +0.03 ± 0.69; n=98 | The intervention group showed greater numerical improvement than the comparator |
| 65.Özden, F. et al.[34] | SF-36 | Telerehab (TR): Video-guided family sports + Education (online push/message) baseline 42.62 ± 18.76; post 65.12 ± 14.63; change +22.5 ± 66.74; n=20 | Active control baseline 55 ± 15.38; post 61 ± 16.16; change +6 ± 54.74 | The intervention group showed greater numerical improvement than the comparator |
| 66.Park, C.et al.[35] | SF-12 | DPT (AI Digital Application) baseline 61.24 ± 6.44; post 75.2 ± 6.18; change +13.96 ± 75.45 | Active control baseline 48.83 ± 8.46; post 35.38 ± 4.78; change -13.45 ± 35.7 | The intervention group showed greater numerical improvement than the comparator |
| 68.Guerra-Arencibia et al., [36] | SF-12 Physical factor | Telerehabilitation-based exercise (TRG) baseline 42.86 ± 9.31; post 48.14 ± 10.51; change +5.28 ± 7.59; n=18 | Active control baseline 41.8 ± 9.78; post 49.06 ± 7.52; change +7.26 ± 9.13; n=18 | The comparator showed greater numerical improvement than the intervention |
| 70. Hinman et al. [37] | AQoL-6D | Telerehabilitation (Zoom video consultation) change +0.03 ± 0.11; n=190 | Active control change +0.04 ± 0.14; n=182 | The comparator showed greater numerical improvement than the intervention |
| 71. Kang et al.[38] | EQ-5D | Digital therapeutics (MORA Cure LBP, integrating CBT + exercise via smartphone app) baseline 8 ± 2.2; post 7 ± 1.7; change -1 ± 6.32; n=20 | Active control baseline 7.8 ± 2.5; post 7.5 ± 2.2; change -0.3 ± 6.68; n=23 | The comparator showed greater numerical improvement than the intervention |
| 73.Mbada et al.,[39] | SF-12 PHD / Physical Health Domain | （Tele-rehabilitation-based McKenzie Therapy） baseline 57.81 ± 8.99; post 84.12 ± 8.84; change +26.2 ± 9; n=28 | Active control baseline 53.65 ± 13.91; post 77.34 ± 9.31; change +23.6 ± 16.8; n=21 | The intervention group showed greater numerical improvement than the comparator |
| 74. Moutzouri et al.[40] | KOOS QoL | BWR-OPA (Online guidance) baseline 42.1 ± 18.7; post 58.8 ± 14.6; change +16.7 ± 53.03; n=22 | Active control baseline 42.8 ± 19.1; post 53.7 ± 17.3; change +10.9 ± 47.48; n=22 | The intervention group showed greater numerical improvement than the comparator |
| 76.Okudan et al., [41] | SF-12 | Telerehabilitation interventions baseline 40.08 ± 4.87; post 41.09 ± 5.2; change +1.01 ± 6.46; n=15 | Non-active control baseline 39.84 ± 5.36; post 39.72 ± 4.97; change -0.12 ± 5.12; n=15 | The intervention group showed greater numerical improvement than the comparator |
| 78. Shi et al.[42] | SF-36 Physical functioning | Telerehabilitation-based exercise (TBEG) vs Outpatient-based exercise (OBEG) baseline 60.96 ± 2.54; post 76.34; change +15.38 ± 27.81; n=27 | Active control baseline 61.85 ± 12.57; post 78.2; change +16.35 ± 16.62; n=27 | The comparator showed greater numerical improvement than the intervention |
| 79. Barbosa et al.[43] | SF-12 | Telerehab exercise (video calls) + digital booklet change +1.8 ± 4.65; n=33 | Non-active control change +0.4 ± 5.36; n=33 | The intervention group showed greater numerical improvement than the comparator |
| 80. Cetin et al.[44] | SF-36 physical functioning | VR + traditional sports baseline 83.46 ± 11.62; post 86.15 ± 9.82; change +2.69 ± 11.11; n=13 | Active control baseline 92.08 ± 7.82; post 90 ± 7.39; change -2.08 ± 5.24; n=13 | The intervention group showed greater numerical improvement than the comparator |
| 82. Hayashi et al.[45] | EQ-5D-3L | Wearable + telemedicine (therapist guidance) : Fitbit Inspire HR continuous wearing + weekly remote follow-up/education baseline 0.6 ± 0.16; post 0.64 ± 0.16; change +0.04 ± 0.58; n=37 | Non-active control baseline 0.57 ± 0.16; post 0.57 ± 0.19; change 0 ± 0.5; n=34 | The intervention group showed greater numerical improvement than the comparator |
| 83. Sakuma et al.[46] | EQ-5D-5L | Immersive VR (Oculus Quest) baseline 0.76 ± 0.12; post 0.8 ± 0.15; change +0.04 ± 0.01; n=32 | Active control baseline 0.73 ± 0.21; post 0.82 ± 0.31; change +0.09 ± 0.02; n=34 | The comparator showed greater numerical improvement than the intervention |
| 84. Im et al.[47] | SF-36 total score | DHI – Mobile App baseline 73.21 ± 17.92; post 82.19 ± 22.45; change +8.98 ± 73.58; n=16 | Non-active control baseline 68.5 ± 20.44; post 72.4 ± 22.09; change +3.9 ± 64.27; n=10 | The intervention group showed greater numerical improvement than the comparator |
| 85. Ocasar et al.[48] | SF-36V PCS | VR baseline 36.9 ± 6.7; post 38.4; change +1.5 ± 6.65; n=45 | Non-active control baseline 33.5 ± 7.8; post 35.59; change +2.09 ± 6.72; n=41 | The comparator showed greater numerical improvement than the intervention |
| 86. Dieter et al.[49] | VR-12 PCS | DHI – App + Wearable change +1.7 ± 8.6 | Non-active control change -0.4 ± 6.6 | The intervention group showed greater numerical improvement than the comparator |
| 87. Nuhmani et al.[50] | SF-36 (Total Score) | DHI – Telerehab baseline 97.31 ± 9.23; post 98 ± 9.22; change +0.69 ± 9.23; n=16 | Non-active control baseline 95.68 ± 9.23; post 98.75 ± 9.22; change +3.07 ± 9.23; n=16 | The comparator showed greater numerical improvement than the intervention |
| 90.Menekseoglu et al.[51] | EQ-5D-5L | VR group: Digital Health Intervention (DHI) – VR + hypnotherapy baseline 0.6 ± 0.17; post 0.75 ± 0.13; change +0.15 ± 0.15; n=30 | Non-active control baseline 0.59 ± 0.14; post 0.7 ± 0.13; change +0.11 ± 0.14; n=30 | The intervention group showed greater numerical improvement than the comparator |

**Notes:** Data are shown as mean ± SD when available. “Change” denotes the value reported or derivable from postintervention minus baseline scores. Higher scores indicate better health status for AQoL, EQ-5D, KOOS QoL, and SF-12/SF-36 measures; lower scores indicate better health status for NHP and FIQ-R/FIQR. Descriptive statements reflect numerical direction only and should not be interpreted as evidence of statistical significance unless explicitly reported by the original study. NR, not reported; HRQoL, health-related quality of life; PCS, Physical Component Summary; MCS, Mental Component Summary; VAS, visual analogue scale.

1.	Zhu D, Zhao J, Wu T, Zhu B, Wang M, Han T. Effects of a Computer Vision-Based Exercise Application for People With Knee Osteoarthritis: Randomized Controlled Trial. JMIR Mhealth Uhealth. 2025 2025/05/12/;13:e63022. doi: 10.2196/63022.

2.	Östlind E, Eek F, Stigmar K, Ekvall Hansson E. Effects of self-monitoring physical activity with wearable activity trackers on perceived joint function and health-related quality of life in people with hip and knee osteoarthritis: a secondary analysis of a cluster-randomised clinical trial. BMC Musculoskelet Disord. 2025 2025/01/09/;26(1):33. doi: 10.1186/s12891-024-08238-8.

3.	Lo HHM, Fong PYH, Wang B, Fung CL, Wong SY, Sit RWS. Clinical Efficacy of Virtual Reality Cave Automatic Virtual Environments (CAVE) for Chronic Musculoskeletal Pain in Older Adults: A Randomized Controlled Trial. J Am Med Dir Assoc. 2025 2025/01//;26(1):105344. doi: 10.1016/j.jamda.2024.105344.

4.	Lindner N, Kornder N, Heisig J, Becker A, der Wardt VV, Viniol A. Digitally supported physical activity counselling for people with chronic back pain: a randomised controlled parallel feasibility study. BMC Prim Care. 2025 2025/02/27/;26(1):58. doi: 10.1186/s12875-025-02742-z.

5.	Darcy R, Couri J, Newkirk K, Neagu R, Darbhe V, Jayabalan P. Impact of a Sensor-Based Platform on the Outcome of Medial Off-Loader Bracing in Individuals With Knee Osteoarthritis: A Pilot Randomized Clinical Trial. Am J Phys Med Rehabil. 2025 2025/02/01/;104(2):155-62. doi: 10.1097/phm.0000000000002560.

6.	Özden F, Güçlü B, Tümtürk İ, Doğrukök Ö N, İmerci A, Tuğay BU. The effect of visual feedback-based clinical monitoring application in patients with chronic low back pain: a randomized controlled trial. Eur Spine J. 2024 2024/02//;33(2):505-16. doi: 10.1007/s00586-023-08098-1.

7.	Dieter V, Janssen P, Krauss I. Efficacy of the mHealth-Based Exercise Intervention re.flex for Patients With Knee Osteoarthritis: Pilot Randomized Controlled Trial. JMIR Mhealth Uhealth. 2024 2024/09/09/;12:e54356. doi: 10.2196/54356.

8.	Weber F, Stuhrmann S, Blum Y, Kloek C, Veenhof C, Grüneberg C. The Usability And Preliminary Effectiveness Of An App-Based Training And Education Program (Join2move) For People With Hip Or Knee Osteoarthritis: A Pilot Randomized Controlled Trial. Osteoarthritis and Cartilage. 2023 2023;31:S79. doi: 10.1016/j.joca.2023.01.021.

9.	Tore NG, Oskay D, Haznedaroglu S. The quality of physiotherapy and rehabilitation program and the effect of telerehabilitation on patients with knee osteoarthritis. Clin Rheumatol. 2023 2023/03//;42(3):903-15. doi: 10.1007/s10067-022-06417-3.

10.	Thiengwittayaporn S, Wattanapreechanon P, Sakon P, Peethong A, Ratisoontorn N, Charoenphandhu N, et al. Development of a mobile application to improve exercise accuracy and quality of life in knee osteoarthritis patients: a randomized controlled trial. Arch Orthop Trauma Surg. 2023 2023/02//;143(2):729-38. doi: 10.1007/s00402-021-04149-8.

11.	Groenveld TD, Smits MLM, Knoop J, Kallewaard JW, Staal JB, de Vries M, et al. Effect of a Behavioral Therapy-Based Virtual Reality Application on Quality of Life in Chronic Low Back Pain. Clin J Pain. 2023 2023/06/01/;39(6):278-85. doi: 10.1097/ajp.0000000000001110.

12.	Hernando-Garijo I, Ceballos-Laita L, Mingo-Gómez MT, Medrano-de-la-Fuente R, Estébanez-de-Miguel E, Martínez-Pérez MN, et al. Immediate Effects of a Telerehabilitation Program Based on Aerobic Exercise in Women with Fibromyalgia. Int J Environ Res Public Health. 2021 2021/02/20/;18(4). doi: 10.3390/ijerph18042075.

13.	Li LC, Feehan LM, Xie H, Lu N, Shaw CD, Gromala D, et al. Effects of a 12-Week Multifaceted Wearable-Based Program for People With Knee Osteoarthritis: Randomized Controlled Trial. JMIR Mhealth Uhealth. 2020 2020/07/03/;8(7):e19116. doi: 10.2196/19116.

14.	Bennell KL, Campbell PK, Egerton T, Metcalf B, Kasza J, Forbes A, et al. Telephone Coaching to Enhance a Home-Based Physical Activity Program for Knee Osteoarthritis: A Randomized Clinical Trial. Arthritis Care Res (Hoboken). 2017 Jan;69(1):84-94. PMID: 27111441. doi: 10.1002/acr.22915.

15.	Heapy AA, Higgins DM, Goulet JL, LaChappelle KM, Driscoll MA, Czlapinski RA, et al. Interactive Voice Response-Based Self-management for Chronic Back Pain: The COPES Noninferiority Randomized Trial. JAMA Intern Med. 2017 Jun 1;177(6):765-73. PMID: 28384682. doi: 10.1001/jamainternmed.2017.0223.

16.	Lee J, Lee M, Lim T, Kim T, Kim S, Suh D, et al. Effectiveness of an application-based neck exercise as a pain management tool for office workers with chronic neck pain and functional disability: A pilot randomized trial. Eur J Integr Med. 2017 Jun;12:87-92. PMID: WOS:000414230000012. doi: 10.1016/j.eujim.2017.04.012.

17.	Sarig Bahat H, Croft K, Carter C, Hoddinott A, Sprecher E, Treleaven J. Remote kinematic training for patients with chronic neck pain: a randomised controlled trial. Eur Spine J. 2018 Jun;27(6):1309-23. PMID: 29018956. doi: 10.1007/s00586-017-5323-0.

18.	Yuan SLK, Couto LA, Marques AP. Effects of a six-week mobile app versus paper book intervention on quality of life, symptoms, and self-care in patients with fibromyalgia: a randomized parallel trial. Braz J Phys Ther. 2021 Jul-Aug;25(4):428-36. PMID: 33248904. doi: 10.1016/j.bjpt.2020.10.003.

19.	Villafaina S, Collado-Mateo D, Domínguez-Muñoz FJ, Fuentes-García JP, Gusi N. Benefits of 24-Week Exergame Intervention on Health-Related Quality of Life and Pain in Women with Fibromyalgia: A Single-Blind, Randomized Controlled Trial. Games Health J. 2019 Dec;8(6):380-6. PMID: 31259617. doi: 10.1089/g4h.2019.0023.

20.	Almhdawi KA, Obeidat DS, Kanaan SF, Oteir AO, Mansour ZM, Alrabbaei H. Efficacy of an innovative smartphone application for office workers with chronic non-specific low back pain: a pilot randomized controlled trial. Clin Rehabil. 2020 Oct;34(10):1282-91. PMID: 32602362. doi: 10.1177/0269215520937757.

21.	Hinman RS, Campbell PK, Lawford BJ, Briggs AM, Gale J, Bills C, et al. Does telephone-delivered exercise advice and support by physiotherapists improve pain and/or function in people with knee osteoarthritis? Telecare randomised controlled trial. Br J Sports Med. 2020 Jul;54(13):790-7. PMID: 31748198. doi: 10.1136/bjsports-2019-101183.

22.	Malliaras P, Cridland K, Hopmans R, Ashton S, Littlewood C, Page R, et al. Internet and Telerehabilitation-Delivered Management of Rotator Cuff-Related Shoulder Pain (INTEL Trial): Randomized Controlled Pilot and Feasibility Trial. JMIR Mhealth Uhealth. 2020 Nov 18;8(11):e24311. PMID: 33206059. doi: 10.2196/24311.

23.	Sander LB, Paganini S, Terhorst Y, Schlicker S, Lin JX, Spanhel K, et al. Effectiveness of a Guided Web-Based Self-help Intervention to Prevent Depression in Patients With Persistent Back Pain The PROD-BP Randomized Clinical Trial. JAMA Psychiatry. 2020 Oct;77(10):1001-11. PMID: WOS:000582383300008. doi: 10.1001/jamapsychiatry.2020.1021.

24.	Schlicker S, Baumeister H, Buntrock C, Sander L, Paganini S, Lin J, et al. A Web- and Mobile-Based Intervention for Comorbid, Recurrent Depression in Patients With Chronic Back Pain on Sick Leave (Get.Back): Pilot Randomized Controlled Trial on Feasibility, User Satisfaction, and Effectiveness. JMIR Ment Health. 2020 Apr 15;7(4):e16398. PMID: 32293577. doi: 10.2196/16398.

25.	Abadiyan F, Hadadnezhad M, Khosrokiani Z, Letafatkar A, Akhshik H. Adding a smartphone app to global postural re-education to improve neck pain, posture, quality of life, and endurance in people with nonspecific neck pain: a randomized controlled trial. Trials. 2021 Apr 12;22(1):274. PMID: 33845880. doi: 10.1186/s13063-021-05214-8.

26.	Juhlin S, Bergenheim A, Gjertsson I, Larsson A, Mannerkorpi K. Physical activity with person-centred guidance supported by a digital platform for persons with chronic widespread pain: A randomized controlled trial. J Rehabil Med. 2021 Apr 1;53(4):jrm00175. PMID: 33576434. doi: 10.2340/16501977-2796.

27.	Sandal LF, Bach K, Øverås CK, Svendsen MJ, Dalager T, Stejnicher Drongstrup Jensen J, et al. Effectiveness of App-Delivered, Tailored Self-management Support for Adults With Lower Back Pain-Related Disability: A selfBACK Randomized Clinical Trial. JAMA Intern Med. 2021 Oct 1;181(10):1288-96. PMID: 34338710. doi: 10.1001/jamainternmed.2021.4097.

28.	Lara-Palomo IC, Antequera-Soler E, Matarán-Peñarrocha GA, Fernández-Sánchez M, García-López H, Castro-Sánchez AM, et al. Comparison of the effectiveness of an e-health program versus a home rehabilitation program in patients with chronic low back pain: A double blind randomized controlled trial. Digit Health. 2022 Jan-Dec;8:20552076221074482. PMID: 35111332. doi: 10.1177/20552076221074482.

29.	Cetin H, Kose N, Oge HK. Virtual reality and motor control exercises to treat chronic neck pain: A randomized controlled trial. Musculoskelet Sci Pract. 2022 Dec;62:102636. PMID: 35952621. doi: 10.1016/j.msksp.2022.102636.

30.	Gulsen C Pt M, Soke F Pt P, Eldemir K Pt M, Apaydin Y Pt M, Ozkul C Pt P, Guclu-Gunduz A Pt P, et al. Effect of fully immersive virtual reality treatment combined with exercise in fibromyalgia patients: a randomized controlled trial. Assist Technol. 2022 May 4;34(3):256-63. PMID: 32543290. doi: 10.1080/10400435.2020.1772900.

31.	Eccleston C, Fisher E, Liikkanen S, Sarapohja T, Stenfors C, Jääskeläinen SK, et al. A prospective, double-blind, pilot, randomized, controlled trial of an "embodied" virtual reality intervention for adults with low back pain. Pain. 2022 Sep 1;163(9):1700-15. PMID: 35324507. doi: 10.1097/j.pain.0000000000002617.

32.	Stamm O, Dahms R, Reithinger N, Ruß A, Müller-Werdan U. Virtual reality exergame for supplementing multimodal pain therapy in older adults with chronic back pain: a randomized controlled pilot study. Virtual Real. 2022;26(4):1291-305. PMID: 35194374. doi: 10.1007/s10055-022-00629-3.

33.	Marcuzzi A, Nordstoga AL, Bach K, Aasdahl L, Nilsen TIL, Bardal EM, et al. Effect of an Artificial Intelligence-Based Self-Management App on Musculoskeletal Health in Patients With Neck and/or Low Back Pain Referred to Specialist Care: A Randomized Clinical Trial. JAMA Netw Open. 2023 Jun 1;6(6):e2320400. PMID: 37368401. doi: 10.1001/jamanetworkopen.2023.20400.

34.	Özden F, Özkeskin M, Tümtürk İ, Yalın Kılınç C. The effect of exercise and education combination via telerehabilitation in patients with chronic neck pain: A randomized controlled trial. Int J Med Inform. 2023 Dec;180:105281. PMID: 37924590. doi: 10.1016/j.ijmedinf.2023.105281.

35.	Park C, Yi C, Choi WJ, Lim HS, Yoon HU, You SJH. Long-term effects of deep-learning digital therapeutics on pain, movement control, and preliminary cost-effectiveness in low back pain: A randomized controlled trial. Digit Health. 2023 Jan-Dec;9:20552076231217817. PMID: 38053732. doi: 10.1177/20552076231217817.

36.	Guerra-Arencibia L, Santana-Déniz C, Pecos-Martín D, Fernández-Carnero S, de Miguel-Hernando N, Achalandabaso-Ochoa A, et al. Effectiveness of a Telerehabilitation-Based Exercise Program in Patients with Chronic Neck Pain-A Randomized Clinical Trial. Sensors (Basel). 2024 Dec 18;24(24). PMID: 39771803. doi: 10.3390/s24248069.

37.	Hinman RS, Campbell PK, Kimp AJ, Russell T, Foster NE, Kasza J, et al. Telerehabilitation consultations with a physiotherapist for chronic knee pain versus in-person consultations in Australia: the PEAK non-inferiority randomised controlled trial. Lancet. 2024 Mar 30;403(10433):1267-78. PMID: 38461844. doi: 10.1016/s0140-6736(23)02630-2.

38.	Kang DH, Park JH, Yoon C, Choi CH, Lee S, Park TH, et al. Multidisciplinary Digital Therapeutics for Chronic Low Back Pain Versus In-Person Therapeutic Exercise with Education: A Randomized Controlled Pilot Study. J Clin Med. 2024 Dec 4;13(23). PMID: 39685835. doi: 10.3390/jcm13237377.

39.	Mbada C, Olanipekun OO, Ademoyegun A, Egwu MO, Makinde M, Gebrye T, et al. Effectiveness of telerehabilitation-based mckenzie method versus manual therapy in low-back pain management: a randomised controlled trial. Bulletin of Faculty of Physical Therapy. 2024 2024/11/06;29(1):50. doi: 10.1186/s43161-024-00218-2.

40.	Moutzouri M, Koumantakis GA, Hurley M, Kladouchou AG, Gioftsos G. Effectiveness of a Web-Guided Self-Managed Telerehabilitation Program Enhanced with Outdoor Physical Activity on Physical Function, Physical Activity Levels and Pain in Patients with Knee Osteoarthritis: A Randomized Controlled Trial. J Clin Med. 2024 Feb 6;13(4). PMID: 38398248. doi: 10.3390/jcm13040934.

41.	Okudan B, Çelik D, Koban O. The effectiveness of telerehabilitation-based exercise combined with pain neuroscience education for patients with facet joint arthrosis: A randomized controlled study. Eur J Pain. 2024 Mar;28(3):382-95. PMID: 37747343. doi: 10.1002/ejp.2187.

42.	Shi W, Zhang Y, Bian Y, Chen L, Yuan W, Zhang H, et al. The Physical and Psychological Effects of Telerehabilitation-Based Exercise for Patients With Nonspecific Low Back Pain: Prospective Randomized Controlled Trial. JMIR Mhealth Uhealth. 2024 Sep 6;12:e56580. PMID: 39240210. doi: 10.2196/56580.

43.	Barbosa JC, da Luz BV, da Silva BFP, Marques AP, Saragiotto BT, Comachio J, et al. Effectiveness of Telerehabilitation Exercise Programme on Disability and Pain in Patients With Chronic Non-Specific Neck Pain: Randomised Controlled Trial Assessor-Blinded. Musculoskeletal Care. 2025 Jun;23(2):e70119. PMID: 40374317. doi: 10.1002/msc.70119.

44.	Cetin H, Aydogdu S, Onal B, Dulger E, Bilgin S, Oge HK, et al. Immersive virtual reality versus cervical mobilization: The effects on joint position sense and pain for chronic neck pain - a randomized controlled trial. Braz J Phys Ther. 2025 Sep-Oct;29(5):101228. PMID: 40435919. doi: 10.1016/j.bjpt.2025.101228.

45.	Hayashi K, Miki K, Shiro Y, Tetsunaga T, Takasusuki T, Hosoi M, et al. Utilization of telemedicine in conjunction with wearable devices for patients with chronic musculoskeletal pain: a randomized controlled clinical trial. Sci Rep. 2025 Jan 9;15(1):11. PMID: WOS:001394990600039. doi: 10.1038/s41598-024-85056-x.

46.	Sakuma S, Kimpara K, Kawai Y, Yanagita Y, Tanaka N, Tawara Y, et al. Integrating Physical Therapy and Virtual Reality to Manage Pain-Related Fear of Movement in Patients With Chronic Pain: A Randomized Controlled Trial. Cureus. 2025 Feb;17(2):e79551. PMID: 40151697. doi: 10.7759/cureus.79551.

47.	Im YJ, Choi J, Choi SA, Kwon JY, Do JG. Feasibility and Preliminary Effectiveness of a Mobile App-Based Personalized Exercise Program in Older Patients With Chronic Knee Osteoarthritis: Pilot Randomized Controlled Trial. JMIR Mhealth Uhealth. 2025 Dec 16;13:e71073. PMID: 41401375. doi: 10.2196/71073.

48.	Úbeda-D'Ocasar E, Moreno-Crespo Y, Cimadevilla-Fernández-Pola E, Hernández-Lougedo J, Navas-Mosqueda Á, Caballero-Corella M, et al. Therapeutic Use of Virtual Reality for Patients With Fibromyalgia and Chronic Neck Pain: Randomized Controlled Trial. JMIR Rehabil Assist Technol. 2026 Jan 23;13:e81158. PMID: 41575862. doi: 10.2196/81158.

49.	Dieter V, Martus P, Seißler D, Serna-Higuita LM, Janssen P, Krauss I. Effectiveness of the Self-Directed mHealth Exercise Intervention re.flex in Patients With Knee Osteoarthritis: Randomized Controlled Trial. J Med Internet Res. 2025 Oct 9;27:e71558. PMID: 41066658. doi: 10.2196/71558.

50.	Nuhmani S, AlBakheet A, AlQahtani G, AlDulaijan R, AlHomiyn M, Al Saikhan L. Effectiveness of Telerehabilitation in Reducing Pain and Improving Quality of Life and Job Satisfaction Among Cardiac Sonographers with Work-Related Musculoskeletal Disorders: A Randomized Controlled Trial. J Clin Med. 2025 Sep 18;14(18). PMID: 41010780. doi: 10.3390/jcm14186576.

51.	Menekseoglu AK, Asik HK, Sahbaz T, İs EE. Mind over pain: VR-based hypnotherapy for chronic non-specific low back pain - a randomised controlled trial. Ir J Med Sci. 2025 Nov 10. PMID: 41212507. doi: 10.1007/s11845-025-04158-1.
