# Supplementary material for: Effects of Digital Health Interventions in Chronic Musculoskeletal Pain: Systematic Review and Bayesian Network Meta-Analysis of Randomized Controlled Trials
Source: J Med Internet Res. 2026 Oct 7;28:e95903. doi: 10.2196/95903 (PMC13645338; doi:10.2196/95903)
Supplement: Supplementary file 7 [file jmir-v28-e95903-s006.docx]

**Multimedia Appendix 6. Risk of bias assessment for included trials, including individually randomized trials and cluster randomized trials.**

**eTable 1. Assessment of risk of bias using the Cochrane Risk of Bias Tool.**

| **Study ID** | **Randomization process** | **Deviations from intended interventions** | **Missing outcome data** | **Measurement of the outcome** | **Selection of the reported result** | **Overall Bias** |
| --- | --- | --- | --- | --- | --- | --- |
| Zhu et al. (2025) | Low | Some concerns | Low | Low | Low | Some concerns |
| Östlind et al. (2025) | Low | Some concerns | Some concerns | Some concerns | Low | Some concerns |
| Lo et al. (2025) | Low | Low | Some concerns | Low | Low | Some concerns |
| Lindner et al. (2025) | Low | Some concerns | High | High | Low | High |
| Darcy et al. (2025) | Some concerns | Low | Low | Low | Low | Some concerns |
| Özden et al. (2024) | Some concerns | Some concerns | Low | Some concerns | Low | Some concerns |
| Nishitha et al. (2024) | Low | Low | Some concerns | Low | Low | Some concerns |
| Kim & Shin (2024) | Some concerns | Low | Low | Some concerns | Low | Some concerns |
| Dieter et al. (2024) | Low | Low | Low | Low | Low | Low |
| Weber et al. (2023) | Low | Low | Low | Some concerns | Low | Some concerns |
| Tore et al. (2023) | Some concerns | Low | Low | Low | Low | Some concerns |
| Thiengwittayaporn et al. (2023) | Low | Low | Low | Low | Low | Low |
| Rodríguez et al. (2023) | Low | Low | Low | Low | Low | Low |
| Özlü et al. (2023) | Some concerns | Low | Low | Low | Low | Some concerns |
| Lee et al. (2023) | Some concerns | Low | Low | Low | Low | Some concerns |
| Groenveld et al. (2023) | Some concerns | Low | Low | Low | Low | Some concerns |
| Hernando-Garijo et al. (2021) | Low | Low | Low | Low | Low | Low |
| Gohir et al. (2021) | Low | Low | Low | Low | Low | Low |
| Li et al. (2020) | Low | Low | Low | Low | Low | Low |
| Petrozzi et al. (2019) | Low | Low | Low | Low | Low | Low |
| Rezaei et al. (2019) | Low | Low | Low | Low | Low | Low |
| Irvine et al. (2015) | Low | Low | Low | Low | Low | Low |
| Bahat et al. (2015) | Low | High | Some concerns | Low | Low | High |
| Rini et al. (2015) | Low | Some concerns | Some concerns | Some concerns | Low | Some concerns |
| Thomas et al. (2016) | Low | Some concerns | Low | Some concerns | Low | Some concerns |
| Bennell et al. (2017) | Low | Some concerns | Low | Some concerns | Low | Some concerns |
| Hagiwara et al. (2017) | Low | Some concerns | Some concerns | Some concerns | Low | Some concerns |
| Heapy et al.（2017） | Low | Low | Low | Low | Low | Low |
| Lee et al. (2017) | Low | Some concerns | Low | Some concerns | Low | Some concerns |
| Bahat et al. (2018) | Low | Low | Low | Low | Low | Low |
| Chhabra et al. (2018) | Low | Low | Low | Low | Low | Low |
| Rutledge et al. (2018) | Low | Low | Low | Low | Low | Low |
| Yuan SLK et al. (2021) | Low | Low | Low | Low | Low | Low |
| Amorim AB et al. (2019) | Low | Low | Low | Low | Some concerns | Some concerns |
| Raad Shebib et al. (2019) | Low | Low | Low | Low | Low | Low |
| Villafaina S et al. (2019) | Low | Low | Low | Low | Low | Low |
| Almhdawi et al. (2020) | Low | Some concerns | Low | Low | Low | Some concerns |
| Ditchburn JL et al. (2020) | Low | Low | Low | Low | Low | Low |
| Greenberg J et al. (2020) | Low | Some concerns | Some concerns | Low | Low | Some concerns |
| Hinman RS et al. (2020) | Low | Low | Low | Some concerns | Low | Some concerns |
| Lam J et al. (2020) | Some concerns | High | High | Some concerns | Some concerns | High |
| Malliaras P et al. (2020) | Low | Low | Low | Some concerns | Low | Low |
| Tejera DM et al. (2020) | Low | Low | Low | Some concerns | Low | Some concerns |
| Sander LB et al. (2020) | Low | Low | Low | Low | Low | Low |
| Schlicker S et al. (2020) | Low | Low | Some concerns | Some concerns | Low | Some concerns |
| Suso-Ribera C et al (2020) | Low | Low | Some concerns | Some concerns | Low | Some concerns |
| Abadiyan F et al (2021) | Low | Some concerns | Low | Low | Low | Some concerns |
| Dadarkhah A et al. (2021) | Low | Some concerns | Low | Some concerns | Low | Some concerns |
| Juhlin S et al. (2021) | Low | Some concerns | Some concerns | Some concerns | Low | Some concerns |
| Nambi G et al. (2021) | Low | Low | Low | Some concerns | Low | Some concerns |
| Sandal LF et al. (2021) | Low | Low | Low | Some concerns | Low | Some concerns |
| Lara-Palomo IC et al. (2022) | Some concerns | Some concerns | High | Some concerns | Some concerns | High |
| Cetin H et al. (2022) | Low | Some concerns | Low | Some concerns | Low | Some concerns |
| Gulsen C PT et al. (2022) | Some concerns | Some concerns | Some concerns | Some concerns | Low | Some concerns |
| Eccleston C et al (2022) | Low | Low | Low | Low | Some concerns | Some concerns |
| Pach D et al. (2022) | Low | Low | Some concerns | Some concerns | Low | Some concerns |
| Shaygan M et al. (2022) | Low | Some concerns | Some concerns | Some concerns | Low | Some concerns |
| Stamm O et al. (2022) | Some concerns | Low | Some concerns | Some concerns | Low | Some concerns |
| Weise, H. et al. (2022) | Low | Low | Low | Some concerns | Low | Some concerns |
| Yeh, C. H.et al. (2022) | Low | Low | Some concerns | Some concerns | Low | Some concerns |
| Cui, D. et al. (2023) | Some concerns | Low | Some concerns | Some concerns | Low | Some concerns |
| Emedoli, D. et al. (2024) | Low | Low | Low | Some concerns | Low | Some concerns |
| Maddox, T.et al. (2023) | Low | Low | Low | Low | Low | Low |
| Marcuzzi, A et al. (2023) | Low | Low | Low | Some concerns | Low | Some concerns |
| Özden, F. et al. (2023) | Some concerns | Some concerns | Low | Some concerns | Low | Some concerns |
| Park, C.et al. (2023) | Some concerns | Some concerns | Some concerns | Low | Low | Some concerns |
| Villatoro-Luque, F. J. et al. (2023) | Low | Low | Low | Some concerns | Low | Some concerns |
| Guerra-Arencibia, L.et al. (2024) | Low | Some concerns | Low | Some concerns | Low | Some concerns |
| Guo, Q. et al. (2024) | Low | Low | Low | Some concerns | Low | Some concerns |
| Hinman, R. S.et al. (2024) | Low | Low | Low | Low | Low | Low |
| Kang, D. H. et al. (2024) | Low | Some concerns | Low | Some concerns | Low | Some concerns |
| Maddox, T.et al. (2024) | Low | Some concerns | Low | Low | Low | Some concerns |
| Mbada et al. (2024) | Low | Some concerns | Low | Some concerns | Low | Some concerns |
| Moutzouri, M. et al. (2024) | Low | Some concerns | Some concerns | Some concerns | Low | Some concerns |
| Nilmart, P.et al. (2024) | Low | Low | Low | Some concerns | Low | Some concerns |
| Okudan, B.et al. (2024) | Low | Some concerns | Low | Some concerns | Low | Some concerns |
| Onan, D. et al. (2024) | Low | Low | Low | Some concerns | Low | Some concerns |
| Shi, W. et al. (2024) | Low | Low | Some concerns | Some concerns | Low | Some concerns |
| Barbosa, J. C.et al. (2025) | Low | Low | Some concerns | Some concerns | Low | Some concerns |
| Cetin, H.et al. (2025) | Low | Low | Low | Some concerns | Low | Some concerns |
| Chittaro, L.et al. (2025) | Low | Low | Low | Some concerns | Low | Some concerns |
| Hayashi, K.et al. (2025) | Low | Low | Low | Some concerns | Low | Some concerns |
| Sakuma, S.et al. (2025) | Low | Some concerns | Low | Some concerns | Low | Some concerns |
| Yu Jin Im .et al. (2025) | Low | Some concerns | Low | Some concerns | Low | Some concerns |
| Ocasar.et al. (2026) | Some concerns | Some concerns | Low | Some concerns | Low | Some concerns |
| Valerie Dieter.et al. (2025) | Low | Some concerns | Low | Some concerns | Low | Some concerns |
| Shibili Nuhmani.et al. (2025) | Some concerns | Some concerns | Low | Some concerns | Low | Some concerns |
| Emrullah Alkan.et al. (2025) | Low | Some concerns | Low | Some concerns | Low | Some concerns |
| Patcharin Nilmart.et al. (2025) | Low | Some concerns | Low | Some concerns | Low | Some concerns |
| Menekseoglu, A. K.et al. (2025) | Low | Some concerns | Low | Some concerns | Low | Some concerns |
| LynnL.DeBar.et al. (2025) | Low | Some concerns | Low | Some concerns | Low | Some concerns |
| Fulden Sari.et al. (2025) | Low | Some concerns | Low | Some concerns | Low | Some concerns |

**eTable 2(A). Assessment of the risk of bias in all included studies(A) Graph of Risk of Bias; (B) Summary of Risk of Bias.**

| **Study ID** | **D1** | **D2** | **D3** | **D4** | **D5** | **Overall** |  |  |
| --- | --- | --- | --- | --- | --- | --- | --- | --- |
| Zhu et al. (2025) | **+** | **!** | **+** | **+** | **+** | **!** | Low risk  **+**  **!**  Some concerns  **-**  High risk  D5 Randomization process  D2 Deviations from intended interventions  D3 Mising outcome data  D4 Measurement of the outcome  D5 Selection of the reported result | |
| Östlind et al. (2025) | **+** | **!** | **!** | **!** | **+** | **!** |  |  |
| Lo et al. (2025) | **+** | **+** | **!** | **+** | **+** | **!** |  |  |
| Lindner et al. (2025) | **+** | **!** | **-** | **-** | **+** | **-** |  |  |
| Darcy et al. (2025) | **!** | **+** | **+** | **+** | **+** | **!** |  |  |
| Özden et al. (2024) | **!** | **!** | **+** | **!** | **+** | **!** |  |  |
| Nishitha et al. (2024) | **+** | **+** | **!** | **+** | **+** | **!** |  |  |
| Kim & Shin (2024) | **!** | **+** | **+** | **!** | **+** | **!** |  |  |
| Dieter et al. (2024) | **+** | **+** | **+** | **+** | **+** | **+** |  |  |
| Weber et al. (2023) | **+** | **+** | **+** | **!** | **+** | **!** |  |  |
| Tore et al. (2023) | **!** | **+** | **+** | **+** | **+** | **!** |  |  |
| Thiengwittayaporn et al. (2023) | **+** | **+** | **+** | **+** | **+** | **+** |  |  |
| Rodríguez et al. (2023) | **+** | **+** | **+** | **+** | **+** | **+** |  |  |
| Özlü et al. (2023) | **!** | **+** | **+** | **+** | **+** | **!** |  |  |
| Lee et al. (2023) | **!** | **+** | **+** | **+** | **+** | **!** |  |  |
| Groenveld et al. (2023) | **!** | **+** | **+** | **+** | **+** | **!** |  |  |
| Hernando-Garijo et al. (2021) | **+** | **+** | **+** | **+** | **+** | **+** |  |  |
| Gohir et al. (2021) | **+** | **+** | **+** | **+** | **+** | **+** |  |  |
| Li et al. (2020) | **+** | **+** | **+** | **+** | **+** | **+** |  |  |
| Petrozzi et al. (2019) | **+** | **+** | **+** | **+** | **+** | **+** |  |  |
| Rezaei et al. (2019) | **+** | **+** | **+** | **+** | **+** | **+** |  |  |
| Irvine et al. (2015) | **+** | **+** | **+** | **+** | **+** | **+** |  |  |
| Bahat et al. (2015) | **+** | **-** | **!** | **+** | **+** | **-** |  |  |
| Rini et al. (2015) | **+** | **!** | **!** | **!** | **+** | **!** |  |  |
| Thomas et al. (2016) | **+** | **!** | **+** | **!** | **+** | **!** |  |  |
| Bennell et al. (2017) | **+** | **!** | **+** | **!** | **+** | **!** |  |  |
| Hagiwara et al. (2017) | **+** | **!** | **!** | **!** | **+** | **!** |  |  |
| Heapy et al.（2017） | **+** | **+** | **+** | **+** | **+** | **+** |  |  |
| Lee et al. (2017) | **+** | **!** | **+** | **!** | **+** | **!** |  |  |
| Bahat et al. (2018) | **+** | **+** | **+** | **+** | **+** | **+** |  |  |
| Chhabra et al. (2018) | **+** | **+** | **+** | **+** | **+** | **+** |  |  |
| Rutledge et al. (2018) | **+** | **+** | **+** | **+** | **+** | **+** |  |  |
| Yuan SLK et al. (2021) | **+** | **+** | **+** | **+** | **+** | **+** |  |  |
| Amorim AB et al. (2019) | **+** | **+** | **+** | **+** | **!** | **!** |  |  |
| Raad Shebib et al. (2019) | **+** | **+** | **+** | **+** | **+** | **+** |  |  |
| Villafaina S et al. (2019) | **+** | **+** | **+** | **+** | **+** | **+** |  |  |
| Almhdawi et al. (2020) | **+** | **!** | **+** | **+** | **+** | **!** |  |  |
| Ditchburn JL et al. (2020) | **+** | **+** | **+** | **+** | **+** | **+** |  |  |
| Greenberg J et al. (2020) | **+** | **!** | **!** | **+** | **+** | **!** |  |  |
| Hinman RS et al. (2020) | **+** | **+** | **+** | **!** | **+** | **!** |  |  |
| Lam J et al. (2020) | **!** | **-** | **-** | **!** | **!** | **-** |  |  |
| Malliaras P et al. (2020) | **+** | **+** | **+** | **!** | **+** | **+** |  |  |
| Tejera DM et al. (2020) | **+** | **+** | **+** | **!** | **+** | **!** |  |  |
| Sander LB et al. (2020) | **+** | **+** | **+** | **+** | **+** | **+** |  |  |
| Schlicker S et al. (2020) | **+** | **+** | **!** | **!** | **+** | **!** |  |  |
| Suso-Ribera C et al (2020) | **+** | **+** | **!** | **!** | **+** | **!** |  |  |
| Abadiyan F et al (2021) | **+** | **!** | **+** | **+** | **+** | **!** |  |  |
| Dadarkhah A et al. (2021) | **+** | **!** | **+** | **!** | **+** | **!** |  |  |
| Juhlin S et al. (2021) | **+** | **!** | **!** | **!** | **+** | **!** |  |  |
| Nambi G et al. (2021) | **+** | **+** | **+** | **!** | **+** | **!** |  |  |
| Sandal LF et al. (2021) | **+** | **+** | **+** | **!** | **+** | **!** |  |  |
| Lara-Palomo IC et al. (2022) | **!** | **!** | **-** | **!** | **!** | **-** |  |  |
| Cetin H et al. (2022) | **+** | **!** | **+** | **!** | **+** | **!** |  |  |
| Gulsen C PT et al. (2022) | **!** | **!** | **!** | **!** | **+** | **!** |  |  |
| Eccleston C et al (2022) | **+** | **+** | **+** | **+** | **!** | **!** |  |  |
| Pach D et al. (2022) | **+** | **+** | **!** | **!** | **+** | **!** |  |  |
| Shaygan M et al. (2022) | **+** | **!** | **!** | **!** | **+** | **!** |  |  |
| Stamm O et al. (2022) | **!** | **+** | **!** | **!** | **+** | **!** |  |  |
| Weise, H. et al. (2022) | **+** | **+** | **+** | **!** | **+** | **!** |  |  |
| Yeh, C. H.et al. (2022) | **+** | **+** | **!** | **!** | **+** | **!** |  |  |
| Cui, D. et al. (2023) | **!** | **+** | **!** | **!** | **+** | **!** |  |  |
| Emedoli, D. et al. (2024) | **+** | **+** | **+** | **!** | **+** | **!** |  |  |
| Maddox, T.et al. (2023) | **+** | **+** | **+** | **+** | **+** | **+** |  |  |
| Marcuzzi, A et al. (2023) | **+** | **+** | **+** | **!** | **+** | **!** |  |  |
| Özden, F. et al. (2023) | **!** | **!** | **+** | **!** | **+** | **!** |  |  |
| Park, C.et al. (2023) | **!** | **!** | **!** | **+** | **+** | **!** |  |  |
| Villatoro-Luque, F. J. et al. (2023) | **+** | **+** | **+** | **!** | **+** | **!** |  |  |
| Guerra-Arencibia, L.et al. (2024) | **+** | **!** | **+** | **!** | **+** | **!** |  |  |
| Guo, Q. et al. (2024) | **+** | **+** | **+** | **!** | **+** | **!** |  |  |
| Hinman, R. S.et al. (2024) | **+** | **+** | **+** | **+** | **+** | **+** |  |  |
| Kang, D. H. et al. (2024) | **+** | **!** | **+** | **!** | **+** | **!** |  |  |
| Maddox, T.et al. (2024) | **+** | **!** | **+** | **+** | **+** | **!** |  |  |
| Mbada et al. (2024) | **+** | **!** | **+** | **!** | **+** | **!** |  |  |
| Moutzouri, M. et al. (2024) | **+** | **!** | **!** | **!** | **+** | **!** |  |  |
| Nilmart, P.et al. (2024) | **+** | **+** | **+** | **!** | **+** | **!** |  |  |
| Okudan, B.et al. (2024) | **+** | **!** | **+** | **!** | **+** | **!** |  |  |
| Onan, D. et al. (2024) | **+** | **+** | **+** | **!** | **+** | **!** |  |  |
| Shi, W. et al. (2024) | **+** | **+** | **!** | **!** | **+** | **!** |  |  |
| Barbosa, J. C.et al. (2025) | **+** | **+** | **!** | **!** | **+** | **!** |  |  |
| Cetin, H.et al. (2025) | **+** | **+** | **+** | **!** | **+** | **!** |  |  |
| Chittaro, L.et al. (2025) | **+** | **+** | **+** | **!** | **+** | **!** |  |  |
| Hayashi, K.et al. (2025) | **+** | **+** | **+** | **!** | **+** | **!** |  |  |
| Sakuma, S.et al. (2025) | **+** | **!** | **+** | **!** | **+** | **!** |  |  |
| Yu Jin Im .et al. (2025) | **+** | **!** | **+** | **!** | **+** | **!** |  |  |
| Ocasar.et al. (2026) | **!** | **!** | **+** | **!** | **+** | **!** |  |  |
| Valerie Dieter.et al. (2025) | **+** | **!** | **+** | **!** | **+** | **!** |  |  |
| Shibili Nuhmani.et al. (2025) | **!** | **!** | **+** | **!** | **+** | **!** |  |  |
| Emrullah Alkan.et al. (2025) | **+** | **!** | **+** | **!** | **+** | **!** |  |  |
| Patcharin Nilmart.et al. (2025) | **+** | **!** | **+** | **!** | **+** | **!** |  |  |
| Menekseoglu, A. K.et al. (2025) | **+** | **!** | **+** | **!** | **+** | **!** |  |  |
| LynnL.DeBar.et al. (2025) | **+** | **!** | **+** | **!** | **+** | **!** |  |  |
| Fulden Sari.et al. (2025) | **+** | **!** | **+** | **!** | **+** | **!** |  |  |

**eTable 2(B). Summary of Risk of Bias**

As percentage (intention-to-treat)

|  | Low risk | Some concerns | High risk |
| --- | --- | --- | --- |
| Randomization process | 83 | 17 | 0 |
| Deviations from intended interventions | 58.5 | 39.4 | 2.1 |
| Mising outcome data | 75.5 | 21.3 | 3.2 |
| Measurement of the outcome | 38.3 | 60.6 | 1.1 |
| Selection of the reported result | 95.7 | 4.3 | 0 |
| Overall Bias | 22.3 | 73.4 | 4.3 |
