# Supplementary material for: Effects of Digital Health Interventions in Chronic Musculoskeletal Pain: Systematic Review and Bayesian Network Meta-Analysis of Randomized Controlled Trials
Source: J Med Internet Res. 2026 Oct 7;28:e95903. doi: 10.2196/95903 (PMC13645338; doi:10.2196/95903)
Supplement: Supplementary file 8 [file jmir-v28-e95903-s007.docx]

**Multimedia Appendix 7. Subgroup analyses of digital health interventions according to their core therapeutic components.**

Figure S1. Forest plot of the subgroup analysis of app-based interventions according to core therapeutic components for pain intensity.

Figure S2. Forest plot of the subgroup analysis of telehealth interventions according to core therapeutic components for pain intensity.

Figure S3. Forest plot of the subgroup analysis of virtual reality interventions according to core therapeutic components for pain intensity.

Figure S4. Forest plot of the subgroup analysis of app-based interventions according to core therapeutic components for functional disability.

Figure S5. Forest plot of the subgroup analysis of telehealth interventions according to core therapeutic components for functional disability.

Figure S6. Forest plot of the subgroup analysis of virtual reality interventions according to core therapeutic components for functional disability.
