# Supplementary material for: Effects of Digital Health Interventions in Chronic Musculoskeletal Pain: Systematic Review and Bayesian Network Meta-Analysis of Randomized Controlled Trials
Source: J Med Internet Res. 2026 Oct 7;28:e95903. doi: 10.2196/95903 (PMC13645338; doi:10.2196/95903)
Supplement: Supplementary file 9 [file jmir-v28-e95903-s008.docx]

**Multimedia Appendix 8. Funnel plots assessing publication bias across the included studies**

**eFigure 1. Funnel plots assessing publication bias in pairwise meta-analyses of pain outcomes.**

**Figure 1A. Funnel plot of app based interventions for pain outcomes.**

**Figure 1B. Funnel plots of virtual reality based interventions for pain outcomes before and after trim and fill adjustment.**

**Figure 1C. Funnel plot of telerehabilitation interventions for pain outcomes.**

**eFigure 2. Funnel plots for publication bias in pairwise meta-analyses of digital health interventions for functional disability.**

**Figure 2A. Funnel plot of app based interventions for functional disability.**

**Figure 2B. Funnel plots of virtual reality based interventions for functional disability before and after trim and fill adjustment.**

**Figure 2C. Funnel plot of telerehabilitation interventions for functional disability.**
