# Supplementary material for: Effects of Digital Health Interventions in Chronic Musculoskeletal Pain: Systematic Review and Bayesian Network Meta-Analysis of Randomized Controlled Trials
Source: J Med Internet Res. 2026 Oct 7;28:e95903. doi: 10.2196/95903 (PMC13645338; doi:10.2196/95903)
Supplement: Supplementary file 10 [file jmir-v28-e95903-s009.docx]

**Multimedia Appendix 9. Sensitivity analyses for pain and Functional disability outcomes, showing Hedges g with 95% confidence intervals and prediction intervals.**

**Figure S1. Sensitivity analysis of virtual reality–based interventions for pain outcomes, part 1**

**Figure S2. Sensitivity analysis of app interventions for pain outcomes, part 2.**

**Figure S3. Sensitivity analysis of telerehabilitation interventions for pain outcomes, part 3.**

**Figure S4. Sensitivity analysis of app interventions for Functional disability outcomes, part 1.**

**Figure S5. Sensitivity analysis of telerehabilitation interventions for Functional disability outcomes, part 2.**

**Figure S6. Sensitivity analysis of virtual reality–based interventions for Functional disability outcomes, part 3.**
