# Supplementary material for: Effects of Digital Health Interventions in Chronic Musculoskeletal Pain: Systematic Review and Bayesian Network Meta-Analysis of Randomized Controlled Trials
Source: J Med Internet Res. 2026 Oct 7;28:e95903. doi: 10.2196/95903 (PMC13645338; doi:10.2196/95903)
Supplement: Supplementary file 11 [file jmir-v28-e95903-s010.docx]

**Multimedia Appendix 10. Convergence diagnostics of the Bayesian network meta-analysis model, including trace plots, posterior density plots, and Gelman–Rubin statistics.**

**Figure S1.** Trace plots and posterior density plots for the Bayesian network meta-analysis of pain outcomes, part 1 of 4.

**Figure S2.** Trace plots and posterior density plots for the Bayesian network meta-analysis of pain outcomes, part 2 of 4.

**Figure S3.** Trace plots and posterior density plots for the Bayesian network meta-analysis of pain outcomes, part 3 of 4.

**Figure S4.** Trace plots and posterior density plots for the Bayesian network meta-analysis of pain outcomes, part 4 of 4.

**Figure S5.** Gelman-Rubin convergence diagnostic plots for the Bayesian network meta-analysis of pain outcomes, part 1 of 2.

**Figure S6.** Gelman-Rubin convergence diagnostic plots for the Bayesian network meta-analysis of pain outcomes, part 2 of 2.

**Figure S7.** Trace plots and posterior density plots for the Bayesian network meta-analysis of functional disability outcomes, part 1 of 4.

**Figure S8.** Trace plots and posterior density plots for the Bayesian network meta-analysis of functional disability outcomes, part 2 of 4.

**Figure S9.** Trace plots and posterior density plots for the Bayesian network meta-analysis of functional disability outcomes, part 3 of 4.

**Figure S10.** Trace plots and posterior density plots for the Bayesian network meta-analysis of functional disability outcomes, part 4 of 4.

**Figure S11.** Gelman-Rubin convergence diagnostic plots for the Bayesian network meta-analysis of functional disability outcomes, part 1 of 2.

**Figure S12.** Gelman-Rubin convergence diagnostic plots for the Bayesian network meta-analysis of functional disability outcomes, part 2 of 2.
