# Supplementary material for: Effects of Digital Health Interventions in Chronic Musculoskeletal Pain: Systematic Review and Bayesian Network Meta-Analysis of Randomized Controlled Trials
Source: J Med Internet Res. 2026 Oct 7;28:e95903. doi: 10.2196/95903 (PMC13645338; doi:10.2196/95903)
Supplement: Supplementary file 12 [file jmir-v28-e95903-s011.docx]

**Multimedia Appendix 11. Forest plots of the Bayesian network meta-analysis, showing standardized mean differences with 95% credible intervals.**

These forest plots present study-level estimates, pooled direct estimates, back-calculated indirect estimates, and consistency-model network estimates for pain intensity and functional disability. Effect estimates are expressed as standardized mean differences with 95% credible intervals. Comparison-specific I² values are shown for the pairwise and network estimates.

Forest plots of the Bayesian network meta-analysis for pain outcomes, showing standardized mean differences with 95% intervalscredible intervals.

Forest plots of the Bayesian network meta-analysis for disability index outcomes, showing mean differences with 95% credible intervals.
