# Supplementary material for: Effects of Digital Health Interventions in Chronic Musculoskeletal Pain: Systematic Review and Bayesian Network Meta-Analysis of Randomized Controlled Trials
Source: J Med Internet Res. 2026 Oct 7;28:e95903. doi: 10.2196/95903 (PMC13645338; doi:10.2196/95903)
Supplement: Supplementary file 13 [file jmir-v28-e95903-s012.docx]

**Multimedia Appendix 12. Node-splitting analyses assessing inconsistency between direct and indirect evidence in the network meta-analysis for pain and functional disability outcomes.**

Multimedia Appendix 10a. Node-splitting analyses assessing inconsistency between direct and indirect evidence in the network meta-analysis for pain outcomes.

Multimedia Appendix 10b. Node-splitting analyses assessing inconsistency between direct and indirect evidence in the network meta-analysis for disability outcomes.
